# Human herpesvirus infections and dementia or mild cognitive impairment: a systematic review and meta-analysis

**DOI:** 10.1038/s41598-019-41218-w

**Published:** 2019-03-18

**Authors:** Charlotte Warren-Gash, Harriet J. Forbes, Elizabeth Williamson, Judith Breuer, Andrew C. Hayward, Angelique Mavrodaris, Basil H. Ridha, Martin N. Rossor, Sara L. Thomas, Liam Smeeth

**Affiliations:** 1 Faculty of Epidemiology & Population Health, London School of Hygiene and Tropical Medicine, London, WC1E 7HT United Kingdom; 2 Division of Infection & Immunity, University College London, Gower Street, London, WC1E 6BT United Kingdom; 3 Institute of Epidemiology and Healthcare, University College London, Gower Street, London, WC1E 6BT United Kingdom; 4 Cambridge Institute of Public Health, University of Cambridge, Forvie site, Robinson Way, Cambridge, CB2 0SR United Kingdom; 5 NIHR University College London Hospitals Biomedical Research Centre, Maple House, Tottenham Court Road, London, W1T 7DN United Kingdom; 6 Dementia Research Centre, Institute of Neurology, University College London, Queen Square, London, WC1N 3BG United Kingdom

## Abstract

Interest is growing in the role of infectious agents in the pathogenesis of dementia, but current evidence is limited. We conducted a systematic review and meta-analysis to investigate the effect of any of eight human herpesviruses on development of dementia or mild cognitive impairment (MCI). We searched the Cochrane Library, Embase, Global Health, Medline, PsycINFO, Scopus, Web of Science, clinical trials registers and grey literature sources from inception to December 2017 for observational studies with cohort, case control or self-controlled designs, or randomised controlled trials of interventions against herpesviruses. Pooled effect estimates and 95% confidence intervals (CIs) were generated through random effects meta-analyses across studies with the same design, outcome, and virus type, method and site of measurement. We included 57 studies across various geographic settings. Past infection with herpesviruses, measured by IgG seropositivity, was generally not associated with dementia risk. A single cohort study rated moderate quality showed an association between varicella zoster virus reactivation (ophthalmic zoster) and incident dementia (HR 2.97; 95%CI, 1.89 to 4.66). Recent infection with, or reactivation of, herpes simplex virus type 1 or type 1/2 unspecified, cytomegalovirus and human herpes virus-6 measured by serum IgM, high titre IgG or clinical disease may be associated with dementia or MCI, though results were inconsistent across studies and overall evidence rated very low quality. Longitudinal population studies with robust repeated virus measurements taken sufficiently proximal to dementia onset are needed to establish whether, when and among whom herpesviruses affect dementia risk.

## Introduction

Whether chronic viral infections increase the risk of dementia remains controversial: despite decades of research, high-quality population studies are lacking. Nevertheless, only around one third of dementia cases are due to modifiable risk factors^1^. This, combined with the projected 2.8-fold increase in the global burden of dementia to 131.5 million cases by 2050^2^, and the lack of effective treatments, has added impetus to the search for novel modifiable risk factors.

Infections with a systemic inflammatory component can trigger brain responses through microglial activation and release of pro-inflammatory mediators^3^. Chronic antigenic stimulation may also contribute to age-associated immune system remodelling and accelerate neurodegeneration in disorders such as dementia^4,5^. In neuronal and glial cell cultures, herpes simplex virus type 1 (HSV-1) – one of eight herpesviruses that routinely infects humans – induces molecular changes similar to those seen in Alzheimer’s disease (AD), e.g. β-amyloid accumulation^6^, generation of amyloid precursor protein fragments with neurotoxic potential^7^, and tau hyperphosphorylation^8^. It remains unclear, however, whether HSV-1 has similar effects *in vivo*.

A previous meta-analysis of small case-control studies of varying quality, with heterogeneous methods of herpesvirus detection and dementia diagnosis, suggested tentative associations between HSV-1 and AD (pooled OR 1.38; 95%CI, 1.03–1.84) and Epstein-Barr virus (EBV) and AD (pooled OR 1.55; 95%CI, 1.12–2.13)^9^. However, interpretation is challenging as data were pooled across studies detecting herpesviruses from different body sites; past infections were not differentiated from recent infections, nor primary infection from virus reactivation. Here, we systematically reviewed literature on infection with, or reactivation of, any of the eight human herpesviruses and risk of developing dementia or mild cognitive impairment (MCI).

## Methods

This systematic review and meta-analysis was performed in accordance with PRISMA guidelines^10^, and registered with the International Prospective Register of Systematic Reviews on 7 January 2017 (Registration no: CRD42017054684).

### Search strategy

We searched the Cochrane Library, Embase, Global Health, Medline, PsycINFO, Scopus, Web of Science and grey literature sources for articles in any language reporting associations between any human herpesvirus and dementia or MCI in adults in any setting. Searches were conducted from database inception to 7 March 2017. Upon identifying a key paper published after this date, we repeated searches in 7 December 2017 limited to the years 2016 and 2017 (see data supplement for full search strategy).

### Study selection

We included studies of infection with, reactivation of, and where relevant, vaccination against or treatment of, HSV-1, HSV-2, varicella zoster virus (VZV), EBV, cytomegalovirus (CMV) and human herpesviruses types 6, 7 and 8 (HHV-6,-7,-8), defined by clinical or laboratory criteria. Primary outcomes were dementia (all types) and MCI. We included randomised controlled trials (RCTs), cohort, case-control, case crossover and self-controlled case series studies with sufficient data to calculate effect estimates. A detailed protocol is published elsewhere^11^. Two reviewers (HJF, CWG) scanned all titles and abstracts in parallel to select full-text manuscripts for review. Full-text manuscripts were then reviewed in parallel and reasons for exclusion recorded. A third reviewer (LS) resolved any discrepancies. Where relevant, we emailed authors to obtain missing information.

### Data extraction

Two reviewers (HJF, CWG) extracted data according to a standardised data extraction template in parallel for the first three studies, then one reviewer (HJF) extracted data for remaining studies. We included information on author, year, design, setting, study population, and definition and ascertainment of exposures, outcomes and comparators. We also extracted population size, follow up time, number of subjects with the outcome (or exposure for case-control studies), statistical methods, main results and sub-group analyses. Where only raw data were reported, we calculated unadjusted effect estimates and 95% confidence intervals using appropriate online calculators.

### Risk of bias and quality assessment

Risk of bias was assessed in line with the Cochrane collaboration approach^12,13^. For observational studies, we considered biases due to confounding, participant selection, misclassification of variables, missing data and reverse causation. For the intervention study, we included domains relevant to RCTs (selection bias, performance bias, detection bias, attrition bias and reporting bias). Two reviewers assessed risk of bias in parallel for the first three studies, and then one reviewer assessed the remaining studies. We classified each domain as ‘High’, ‘Moderate’, ‘Low’ or ‘Unclear’ risk and, in a sensitivity analysis, removed studies with >one domain rated ‘High risk’. Publication bias was investigated when there were at least ten studies, using a funnel plot and Begg’s test^14^. Finally, we used Grading of Recommendations, Assessment, Development and Evaluation (GRADE)^15^, to estimate the quality of cumulative evidence by virus exposure and outcome (see data supplement).

### Statistical analysis

Results were presented by exposure (virus type) and outcome (dementia or MCI). Exposures were subdivided into recent infection/reactivation (measured by viral nucleic acid, serum IgM, intrathecal antibody synthesis, high serum IgG titre or a recent history of clinical disease) and past infection (measured by IgG seropositivity). We meta-analysed data if there were at least two studies with the same outcome, design and detailed exposure criteria (virus type, method and site of exposure measurement). We conducted meta-analyses using both fixed and random effects models but presented final results from random effects models only for consistency: random effects models were judged more appropriate when there was at least moderate heterogeneity (I^2^ > 25%). Sub-group analyses e.g. by age, APOE4 status and underlying cause of dementia were considered where appropriate.

## Results

### Study characteristics

**Figure 1:**
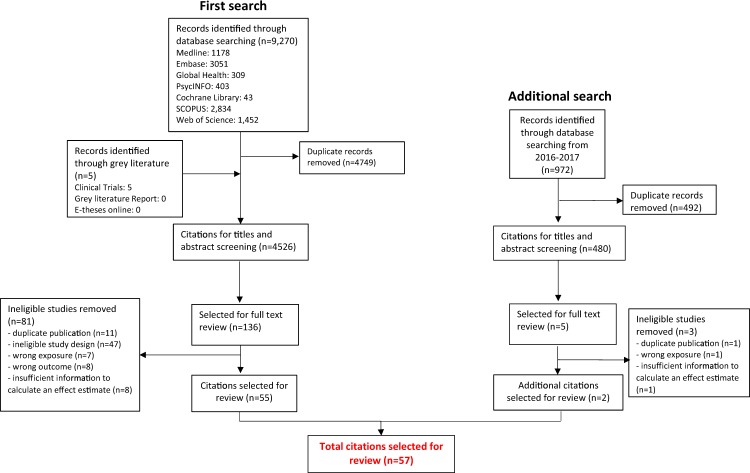
Flow diagram of study selection.

Initial searches retrieved 4,526 citations, of which 136 were selected for full-text review and 55 included. Updated searches retrieved 480 citations, of which five full texts were reviewed and two additional studies included (Fig. 1). The 57 studies (43 case-control, 13 cohort, one RCT) were published between 1974 and 2017 in various geographic settings (UK (n = 14), rest of Europe (n = 22), Far East (n = 8), USA (n = 7) and elsewhere/not reported (n = 6)). For 46 studies, the outcome was dementia alone (AD (n = 35), AIDS-related dementia (n = 5), vascular dementia (n = 2), mixed or unspecified dementia (n = 4)), five studies investigated AD and/or MCI, while the remaining six studies investigated MCI alone (e-table 1). Studies presented 163 estimates for effects of herpesviruses on dementia or MCI (119 main effects; 44 subgroup analyses: e-table 2).

### Effects of individual herpesviruses

**Figure 2:**
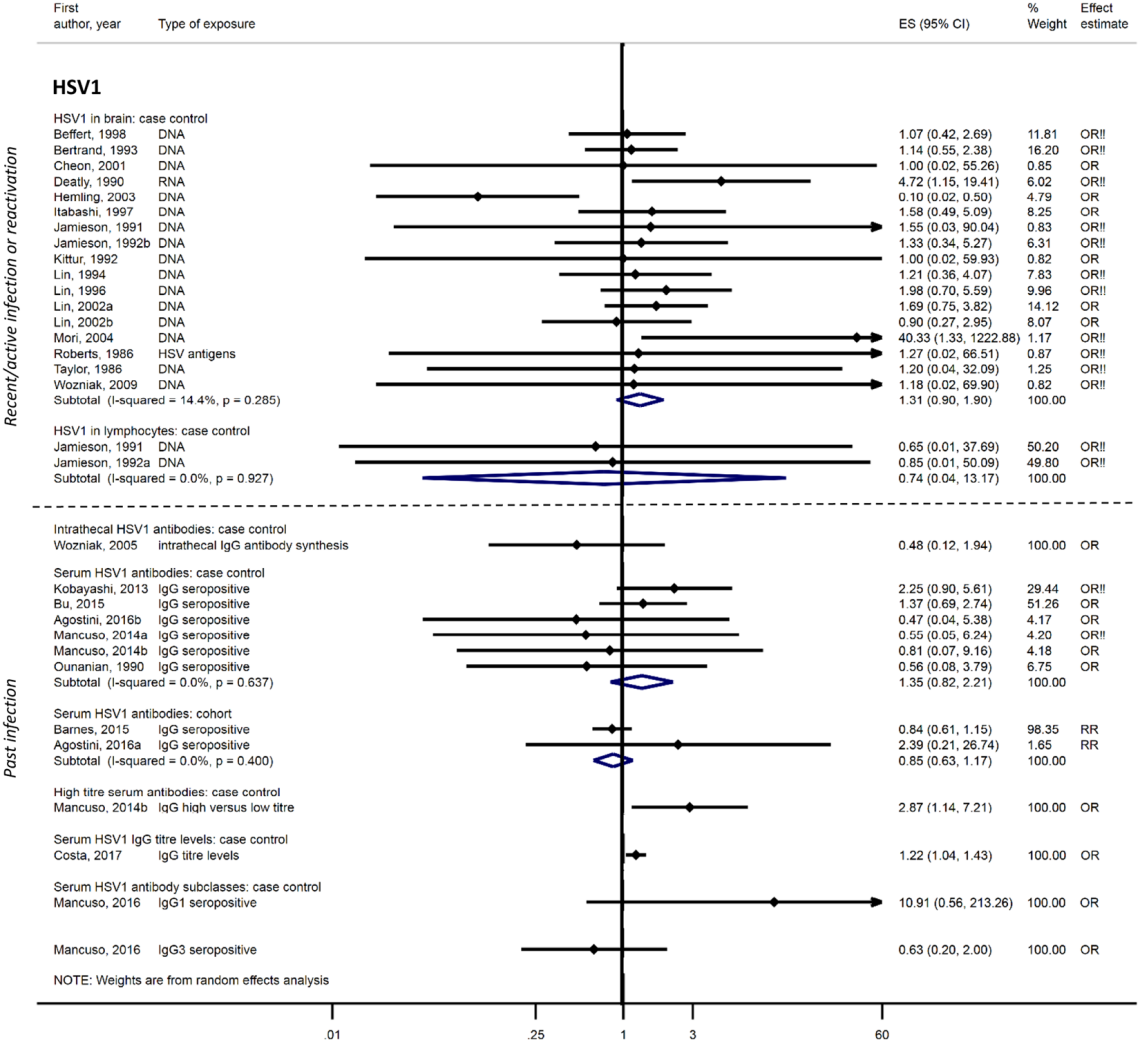
Effect of herpes simplex virus type 1 infections on dementia risk.!! No adjustment/matching for age. One study assessing the effect of HSV1 in the brain (Lin, 1998) is not included here as it uses the same data as another study (Lin, 2002a); however, it has been retained for sub-group analyses.

For HSV-1, 32 case-control^16–47^ and three cohort studies^48–50^ investigated associations with dementia or MCI. Two studies had no domains at high risk of bias^49,50^, three had one domain^38,43,48^ and 30 had ≥two domains at high risk of bias (e-table 3). Seventeen post-mortem case-control studies, all with at least two domains at high risk of bias, showed no evidence of a difference in detection of HSV-1 in the brains of dementia patients compared to controls: pooled OR 1.31; 95%CI, 0.90–1.90, though 14 of 17 studies had <100 participants. There was also no association between HSV1 DNA in lymphocytes (two studies)^22,24^, intrathecal HSV1 antibody synthesis (one study)^36^, HSV1 IgG seropositivity (eight studies)^25,32,33,38,44,47–49^ or IgG subclasses (one study)^31^ and dementia. One case-control study suggested HSV1 IgG titres were higher in AD patients than controls (adjusted OR 1.22; 95%CI, 1.04–1.43)^20^ and in a secondary analysis of another case-control study, high-titre serum IgG was associated with dementia: OR 2.87; 95%CI, 1.14–7.22^32^ (Fig. 2). For MCI, case-control studies showed no association with HSV-1 IgG seropositivity (three studies)^25,33,47^, HSV-1 IgG subclasses (one study)^31^, or HSV-1 detection in blood (method unspecified; one study)^39^ (e-Fig. 1). A population cohort with no domains at high risk of bias also showed no association between rates of cognitive decline over 4 years and HSV-1 baseline antibody titre^50^.

**Figure 3:**
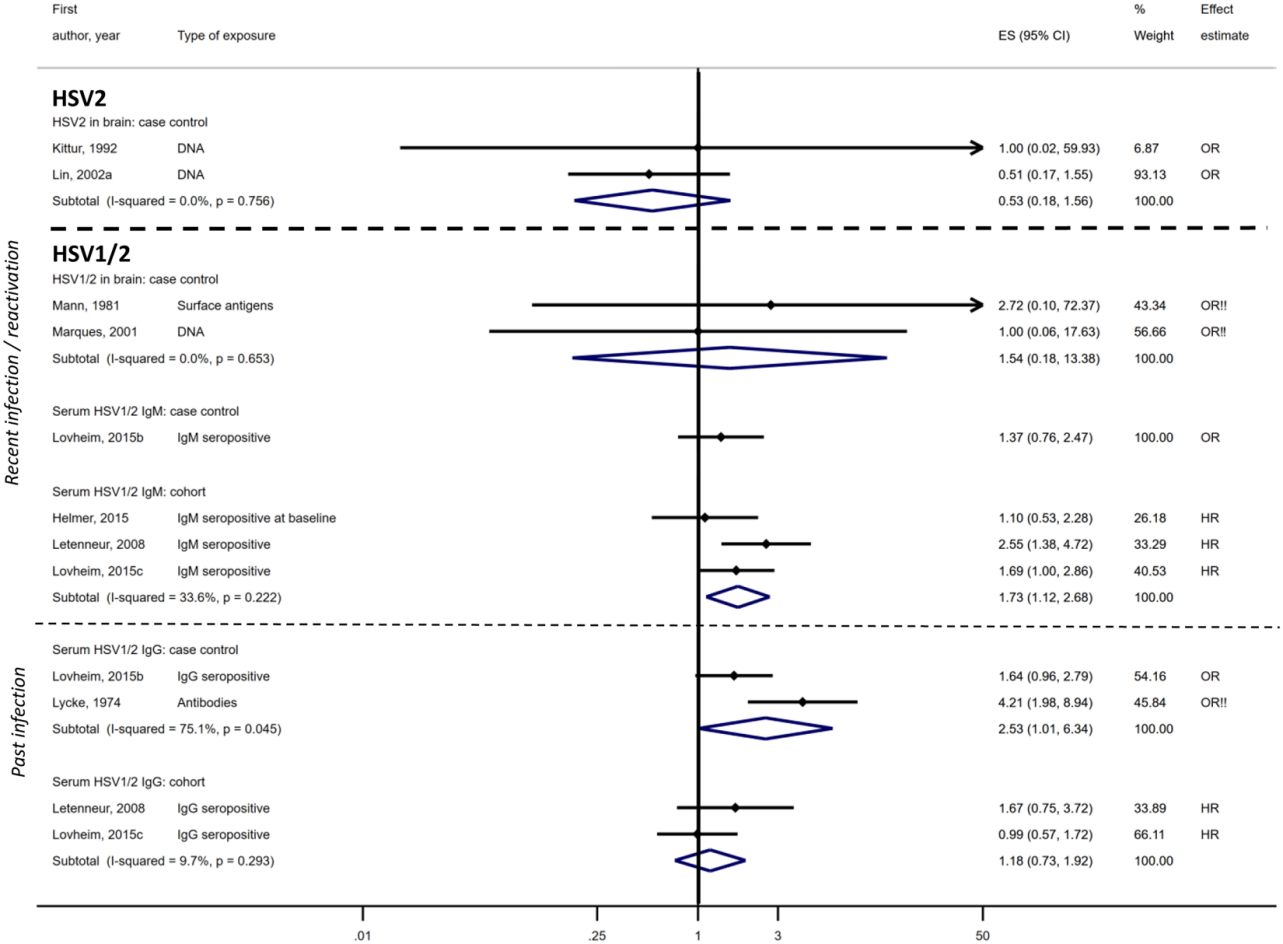
Effect of herpes simplex virus type 2 or herpes simplex type unspecified on dementia risk.!! No adjustment/matching for age.

The effect of HSV-1/2 (type unspecified) was assessed in a further five case-control^51–55^ and three cohort studies^56–58^. Three had no domains at high risk of bias^53,57,58^, one had one domain^56^ and four studies had multiple domains at high risk of bias^51,52,54,55^. Results were mixed; HSV-1/2 in the brain was not associated with dementia in two small post-mortem case-control studies (pooled OR 1.54; 95%CI, 0.18–13.38) (overall n = 71)^51,54^. Serum IgM showed an association with dementia in three cohort studies (pooled OR 1·73; 95%CI, 1.12–2.68) with 0–1 domain at high risk of bias^56–58^ but a case-control study found no effect^53^. IgG seropositivity to HSV-1/2 was not associated with dementia in two cohort studies (pooled OR 1.18; 95%CI, 0.73–1.92)^56,57^ but had a marginal association in two case-control studies (pooled OR 2.53; 95%CI, 1.01–6.34)^52,53^ (Fig. 3). One study found no difference in mean HSV-1/2 serum antibody titres between dementia cases and controls (effect estimates not reported)^55^.

For HSV-2, there were three case-control studies^29,39,42^, all with ≥two domains at high risk of bias. There was no association between HSV-2 virus detected in the brain and dementia in two studies: pooled OR 0.53; 95%CI, 0.18–1.56^29,42^, while the third showed an association between HSV-2 (measurement unspecified) in blood and vascular MCI: unadjusted OR 4.29; 95%CI, 2.01-9.16^39^ (Fig. 3).

**Figure 4:**
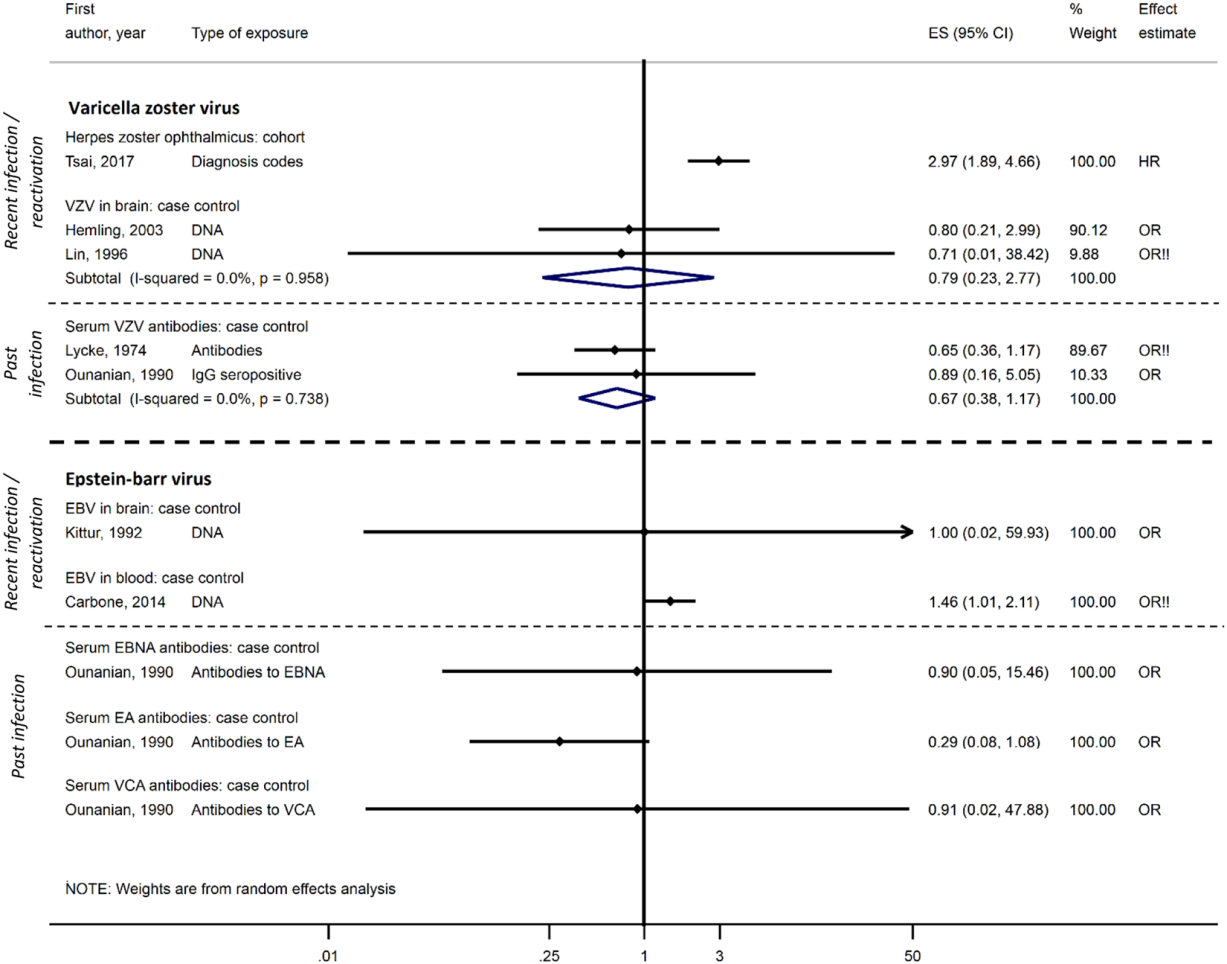
Effect of varicella zoster virus and Epstein-Barr virus on dementia risk.!! No adjustment/matching for age.

The relationship between VZV and dementia was explored in one cohort study^59^, with no domains at high risk of bias, and five case-control studies^27,40,44,46,52^, all with ≥two domains at high risk of bias. The cohort study showed a very strong association between ophthalmic zoster (VZV reactivation) and dementia: adjusted HR 2.97; 95%CI, 1.89-4.66^59^. Neither VZV DNA detected in post-mortem brain samples in two case-control studies (pooled OR 0.79; 95%CI, 0.23–2.77)^27,40^ nor VZV IgG serum antibodies from two other case-control studies (pooled OR 0.67; 95%CI, 0.38–1.17) was associated with dementia^44,52^ (Fig. 4). One additional case-control study reported no association between serum IgG reactivity and AD but did not present estimates^46^.

For EBV, there were three case-control studies^42,44,60^, each with ≥two domains at high risk of bias. EBV DNA in the brain was not associated with dementia in one small study of post-mortem brains (n = 10): OR 1.00; 95%CI, 0.02–59.93^42^, while another study showed a marginal association between EBV DNA in the blood and dementia: unadjusted OR 1.46; 95%CI, 1.01–2.11^60^. The third study found that serum antibodies to three EBV antigens were not associated with dementia^44^ (Fig. 4). One additional nested case-control study with one domain at high risk of bias showed an increase in EBV IgG levels among those developing MCI over two year follow-up (p = 0.003)^43^.

**Figure 5:**
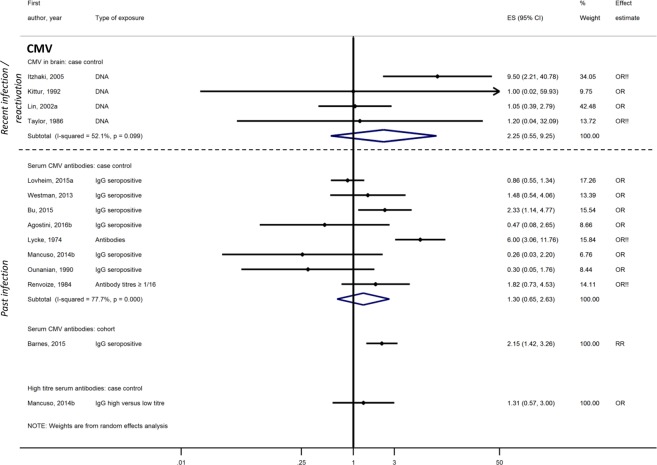
Effect of cytomegalovirus on dementia risk.!! No adjustment/matching for age.

Associations between CMV and dementia or MCI were investigated in 17 case-control^29,30,32,38–41,44–47,52,55,60–63^ and three cohort studies^49,50,64^. Four studies had no domains at high risk of bias^49,50,63,64^, one had one domain^38^ and 15/20 had ≥two domains at high risk of bias. Neither CMV DNA in the brain (pooled OR from four case-control studies 2.25; 95%CI, 0.55–9.24)^29,41,42,45^, CMV-specific serum IgG (pooled OR from eight case-control studies 1.30; 95%CI, 0.65–2.63)^32,38,44,47,52,61–63^, nor high CMV antibody titres (one case-control study)^32^ were associated with dementia. Three additional case-control studies compared blood CMV IgG titres between dementia cases and controls but did not present effect estimates; two reported no difference^46,55^ and one found that AD patients had higher mean antibody titres than controls (p = 0.014)^60^. CMV IgG seropositivity at baseline was associated with AD in one cohort with no domains at high risk of bias: RR 2.15; 95%CI, 1.42–3.27^49^ (Fig. 5). For MCI, one large Mexican-American population cohort with no domains at high risk of bias reported a decline in MMSE score associated with a one unit increase in serum CMV IgG titre (p < 0.001) and a marked difference between MMSE scores of participants with the highest and lowest antibody titres (p = 0.003)^50^. In contrast, a smaller Japanese community cohort study, also with no domains at high risk of bias, showed no association between quartiles of CMV titre and cognitive function^64^. One case-control study found no association between IgG seropositivity and MCI^47^ while another case-control study reported that CMV in blood (detection method unclear) was associated with MCI in unadjusted analysis: OR 2.17; 95%CI, 1.28–3.67^39^.

The relationship between HHV-6 and dementia or MCI was explored in eight case-control studies^29,30,36,40,41,46,47,60^, all with ≥two domains at high risk of bias and one cohort study, which had one domain at high risk of bias^48^. HHV-6 DNA detection in the brain was associated with dementia in three case-control studies: pooled OR 2.47; 95%CI, 1.25–4.86^29,30,40^, and HHV-6 DNA in blood had a marginal association with dementia in data pooled across two further case-control studies: pooled OR 1.22; 95%CI, 1.01–1.47^46,60^. There was no association between HHV-6 intrathecal antibody synthesis and dementia in two case-control studies^36,47^, or between HHV-6 IgG seropositivity and dementia in the cohort study^48^ (e-Fig. 2). One case-control study suggested a negative association between HHV6 seropositivity and MCI: OR 0.32; 95%CI, 0.11–0.97^47^.

For HHV-8 (and/or Kaposi’s sarcoma (KS)) there were two case-control^65,66^ and four cohort studies^67–70^, all carried out among people with HIV. Two studies had no domains at high risk of bias^66,70^, two had one domain^68,69^ and two had two domains at high risk of bias^65,67^. HHV-8 IgG seropositivity was not associated with dementia in either a case-control study: OR 1.22; 95%CI, 0.66-2.27^66^ or two cohort studies: pooled OR 0.79; 95%CI, 0.37–1.69^69,70^, all with 0–1 domain at high risk of bias. KS was not associated with dementia in one case-control study^65^ and had weak evidence of an apparent protective effect against dementia in three cohorts: pooled OR 0.58; 95%CI, 0.33–1.05^67,68,70^ (e-Fig. 2).

One cohort study, with no domains at high risk of bias, investigated the effect of infection with multiple herpesviruses^71^. IgG seropositivity to HSV-1, HSV-2 and CMV was associated with an increased risk of cognitive decline compared to infection with 0–1 virus: OR 2.3; 95%CI, 1.1–5.0, however having two herpesviruses compared to 0–1 was not associated with cognitive decline: OR 1.8; 95%CI, 0.9–3.6.

One RCT^72^ and one cohort study^58^, each with no domains at high risk of bias, investigated the effect of antiviral treatment for HSV-1 on cognition or dementia. The RCT showed that using valacyclovir for 90 days in patients with HSV encephalitis had no effect on cognitive sequelae at 12 months (adjusted OR 0.81; 95%CI, 0.24–2.77)^72^. The population cohort study showed that incident dementia rates were similar for people with and without reimbursement claims data for anti-herpetic medication: adjusted HR 0.75, no CIs presented, p = 0.14^58^.

In sub-group analyses, no differences by age or sex were seen in studies of HSV-1, HSV-1/2, VZV reactivation or HHV-8. Several studies of HSV-1, HSV-1/2, VZV and HHV-6 stratified by APOE4 status, but there were no consistent patterns. Stratifying by dementia subtype gave similar effects to those presented in the main meta-analysis. Finally, removing studies at the highest risk of bias did not result in major differences to findings (e-Figs A1–A3). We did not find evidence of publication bias for studies of HSV-1 DNA in the brain and dementia (p = 0.88) (e-Fig. 4).

Overall, the quality of evidence for VZV reactivation on dementia was judged to be moderate, whilst for every other herpesvirus exposure and dementia or MCI, the quality of evidence was judged to be very low using GRADE criteria, due to serious or very serious risks of bias, inconsistency and imprecision (e-table 4).

## Discussion

Our systematic review showed that, while past herpesvirus infection was generally not associated with dementia risk, there were tentative associations between recent infection with, or reactivation of, several herpesviruses and dementia or MCI, though quality of evidence across the 57 included studies was very low. For HSV-1, HSV-1/2, and HSV-2 there was no evidence that DNA in the brain was associated with dementia and little robust evidence that HSV-1 or HSV-1/2 IgG seropositivity was associated with the risk of dementia or MCI. Although some studies suggested that recent infection or reactivation of HSV-1/2, measured by serum IgM or high titre serum IgG, was associated with dementia, results were inconsistent and evidence graded very low quality. Moderate quality evidence from a single study indicated that clinically-diagnosed VZV reactivation (specifically ophthalmic zoster) was associated with increased risk of dementia, while VZV infection or reactivation from brain or serum samples was not associated with dementia. Findings for EBV and CMV were inconsistent and rated very low quality. Although most studies reported no association with dementia, serum IgG CMV seropositivity was associated with dementia in one cohort, and CMV IgG titres with rates of cognitive decline in another large population cohort, but not in a smaller cohort of the very old. Very low quality evidence suggested that HHV-6 recent infection/reactivation, but not past HHV-6 or HHV-8 infection, was associated with dementia risk.

Human herpesviruses establish latency and persist lifelong after initial infection. Our finding that past infection alone was insufficient to raise dementia risk is consistent with current understanding of neurotropic herpesvirus latency, during which viruses such as HSV-1 and VZV show very restricted gene expression and do not produce infective virus particles or induce clinical disease^73^. Virus reactivation leading to systemic inflammation, glial activation and neuro-inflammation is a putative mechanism to explain our finding that recent infection/reactivation of several herpesviruses may be associated with cognitive decline or dementia^74^. For the most robust relationship identified in our review – that of ophthalmic zoster with dementia – localised vasculopathy, which occurs when VZV replicates in cerebral arteries^75^, may contribute to pathogenesis. For other viruses, potential mechanisms are less well-understood. While recent work showing β-amyloid seeding in response to HSV-1 and HHV-6 infection in mouse models and human neuronal cell cultures supports an anti-microbial peptide role for β-amyloid^76^, the significance for cognitive function in older adults is unknown. A disordered immune response to infections among older people, in part driven by CMV-induced immune system remodelling, has been proposed to promote development of inflammatory disorders such as autoimmune conditions and, potentially, dementia^5^.

Strengths of our review include the comprehensive search strategy covering seven medical databases as well as clinical trials registers and grey literature for articles in any language, setting or time period. We pre-specified and published our analysis plan^11^, which included careful consideration of methods of herpesvirus measurement to avoid inappropriate meta-analyses across heterogeneous exposures. Contact with authors and double reviewing throughout identified several instances of duplicate data published in different conference abstracts, which we excluded from meta-analyses. We also conducted a thorough risk of bias assessment for included studies and assessed the quality of evidence overall for each herpesvirus exposure to guide future research. Nevertheless, the strength of evidence is limited by the poor quality of many studies. This included bias associated with difficulty measuring herpesvirus exposures, other than past infection (denoted by serum IgG). Understanding of the nature of sub-clinical herpesvirus reactivation is limited and there is a dearth of standardised methods to assess viral activation. Even markers such as serum IgM, which is known to be associated with recent infection or reactivation, lack sensitivity. It is also unclear whether peripheral virus reactivation correlates with central nervous system reactivation. While some studies detected herpesvirus nucleic acid in post-mortem brains, it is unclear whether this reflects reactivation or peri-mortem changes associated with neurodegeneration. Our outcome definitions were intentionally broad and likely incorporated heterogeneous neuropathologies leading to dementia, although we did not see differences in results stratified by dementia sub-type. Nevertheless, a lack of consistency of dementia definitions and variable reporting of clinical outcomes makes between-study comparisons challenging. In general, our review found a lack of longitudinal studies with repeated measures of exposure, a lack of control for confounding e.g. by age and sex and many small studies that were underpowered to detect an effect.

In contrast to our findings, one previous systematic review and meta-analysis of 35 studies of herpesviruses and dementia reported an association between any herpesvirus in the brain and AD, and specific associations between HSV-1 and EBV with AD^9^. However, the virus-specific results were pooled across any method of virus detection (PCR, *in-situ* hybridization, serum IgG, serum IgM) at any site (blood, brain or CSF) and study quality was not assessed systematically. We found no other systematic reviews or meta-analyses of individual herpesviruses and dementia. Two cohort studies from Taiwan using the National Health Insurance Database published after the dates of our search strengthened our findings. One study reported that herpes zoster was associated with a small increase in subsequent dementia risk – adjusted HR 1.11; 95% CI, 1.04–1.17^77^. The other assessed the association between clinical presentation of new HSV symptoms (either oral or genital) and dementia in patients aged ≥50 years and found an increased risk of incident dementia (adjusted HR 2.56; 95% CI, 2.35–2.80), although the effect was attenuated after removing dementia diagnoses made in the first year after HSV, which were unlikely to be causally linked (adjusted HR 1.65; 95% CI; 1.44–1.87)^78^. In both studies, antiviral prescriptions after herpesvirus diagnosis were associated with lower dementia rates, although this warrants further investigation.

Our findings that larger longitudinal studies supported a possible association between CMV and dementia or cognitive decline are also consistent with literature highlighting the role of CMV in stimulating ‘inflammaging’ – a state of low-grade chronic inflammation occurring in older individuals which predisposes to a range of adverse health outcomes^79^. Several studies have investigated the effect of infectious burden on cognition. Cross-sectional analyses show that a greater infectious burden, measured by IgG seropositivity to a range of antigens (including but not limited to herpesviruses), is associated with poorer cognitive performance in young to middle-aged adults^80^ and with MCI in older adults^81^ after adjusting for sociodemographic and clinical confounders. One longitudinal study showed a similar association between infectious burden and cognitive decline in the memory domain^82^.

Our review highlights the complexity of studying the effects of viruses typically acquired in childhood on syndromes such as MCI and dementia, which have long latent periods and are associated with subtle behavioural, nutritional and immune changes that may occur many years before diagnosis. Nevertheless, knowledge of the mechanisms triggering dementia onset remains limited. Herpesviruses are common and a potentially tractable target for prevention with vaccines or treatment: the alpha herpesviruses (HSV-1, HSV-2 and VZV) in particular have rapid replication cycles and are susceptible to antiviral agents such as the synthetic nucleoside analogue acyclovir and related drugs. Although no effect of antiviral therapy on cognitive outcomes was seen in an RCT conducted in a population with herpes simplex encephalitis^72^, cognitive sequelae may be driven by encephalitis itself rather than any original causative organism^83^. The effect of antiviral therapy on cognitive outcomes in other HSV infected populations therefore remains unclear. While genetically influenced, herpesvirus acquisition is also socially-patterned, e.g. for CMV, individuals of lower socio-economic status are more likely to become infected earlier and to have higher antibody titres throughout life^77^, possibly reflecting a greater propensity to reactivation.

Future research should focus on high quality population studies using longitudinal designs to investigate effects of infections on long-term health outcomes such as dementia, ideally using repeated measures taken across the life course. For studies of herpesviruses, methods and timing of measurement are critical; strategies may include active surveillance in population cohort studies for viruses under-reported in electronic health records. For maximum benefit to policymakers and clinicians, future research should seek to understand when and in whom herpesvirus reactivation is associated with greatest risk of adverse health outcomes, to guide targeted intervention strategies.

## Supplementary information

Supplementary information
