# Supplementary material for: Human herpesvirus infections and dementia or mild cognitive impairment: a systematic review and meta-analysis
Source: Sci Rep. 2019 Mar 18;9:4743. doi: 10.1038/s41598-019-41218-w (PMC6426940; doi:10.1038/s41598-019-41218-w)
Supplement: Supplementary file 1 — Supplementary information [file 41598_2019_41218_MOESM1_ESM.pdf]

Supplementary information

Human herpesvirus infections and dementia or mild cognitive impairment: a systematic review and meta-analysis

Authors: Charlotte Warren-Gash[1], Harriet J Forbes[1] Elizabeth Williamson[1], Judith Breuer[2], Andrew C Hayward[3] Angelique Mavrodaris[4], Basil H Ridha[5,6], Martin N Rossor[5,6] , Sara L.Thomas[1], Liam Smeeth[1]

Affiliations: [1]Faculty of Epidemiology & Population Health, London School of Hygiene and Tropical Medicine, London, WC1E 7HT, United Kingdom

[2]Division of Infection & Immunity, University College London, Gower Street, London WC1E 6BT, United Kingdom

[3]Institute of Epidemiology and Healthcare, University College London, Gower Street, London WC1E 6BT United Kingdom

[4]Cambridge Institute of Public Health, University of Cambridge, Forvie site, Robinson Way, Cambridge CB2 0SR, United Kingdom

[5]NIHR University College London Hospitals Biomedical Research Centre, Maple House, Tottenham Court Road, London W1T 7DN, United Kingdom

[6]Dementia Research Centre, Institute of Neurology, University College London, Queen Square, London WC1N 3BG, United Kingdom

Contents

Part A: additional detail on methods 2

i. Search terms 2

ii. Changes to the original protocol 10

iii. Extracted data items 10

iv. GRADE assessment of quality: down/ upgrading reasons 11

PART B: main tables 14

etable 1 Study characteristics 14

etable 2 Study results 18

etable 3 Risk of bias summary showing judgements about each risk of bias domain 22

etable 4 Assessment of quality of evidence for outcomes 23

PART C: additional figures 24

efigure 1: Effect of herpesvirus infections on MCI risk 24

efigure 2: Effect of herpesviruses type 6 and 8 on dementia risk 25

efigure 3: Assessment of publication bias for HSV1 DNA in the brain (from case-control studies) as a risk factor for dementia 26

Subgroup analyses 27

efigure A1: Effect of herpesvirus infections on dementia risk, by age and sex 27

efigure A2: Effect of herpesvirus infections on dementia risk, by APOE type 28

efigure A3: Effect of herpesvirus infections on dementia risk, by outcome type 29

PART D: additional tables 32

etable A1: Exploring statistical heterogeneity by removing studies at high risk of bias 32

etable A2 Risk of bias summary with judgement and justification for each risk of bias domain 33

Part A: additional detail on methods

i. Search terms

Search terms for the original searches conducted in February/March 2017 are shown. Searches were repeated in December 2017 (see methods of main paper).

MEDLINE (OVID) search strategy [1946 to February Week 4 2017]

1 exp Herpes simplex
2 exp Herpes simplex virus vaccines
3 exp encephalitis, herpes simplex
4 exp Herpesvirus 1, Human
5 cold sore$.ti,ab.
6 exp Herpesvirus 2, Human
7 (genit$ herpes$ or genit$ sores).ti,ab.
8 exp Chickenpox
9 exp Chickenpox vaccine
10 exp Herpes zoster
11 exp Neuralgia, postherpetic
12 exp Herpesvirus 3, Human
13 exp Encephalitis, varicella zoster
14 (varicella or chickenpox or chicken pox or shingles or VZV or zoster).ti,ab.
15 exp Cytomegalovirus
16 exp Cytomegalovirus vaccines
17 exp Cytomegalovirus infections
18 (CMV or cytomegalovirus).ti,ab.
19 exp Herpesvirus 6, Human
20 Roseolovirus Infections/
21 Exanthema Subitum/
22 (B lymphotropic virus$ or roseola or sixth disease or exanthema subitum or exanthem criticum or Roseolovirus or pseudorubella or three?day fever).ti,ab.
23 exp Herpesvirus 7, Human
24 exp Epstein-Barr virus infections
25 exp Epstein-Barr virus
26 exp Herpesvirus 4, Human
27 (EBV or epstein-barr or burkitt adj5 lymphoma$ or glandular fever or infectious mono$ or mononucleosis or hair$ leukoplak$ or OHL).ti,ab.
28 exp Herpesvirus 8, Human
29 Sarcoma, Kaposi/
30 Lymphoma, Primary Effusion/
31 (kaposi$ sarcoma$ or Primary effusion adj2 lymphoma$ or body cavity adj2 lymphoma$).ti,ab.
32 ((HHV adj1 ("1" or "2" or "3" or "4" or "5" or "6" or "7" or "8")) or (HHV?1 or HHV?2 or HHV?3 or HHV?4 or HHV?5 or HHV?6 or HHV?7 or HHV?8)).ti,ab.
33 (HSV?1 or HSV 1 or HSV?2 or HSV 2).ti,ab.
34 herpes$.ti, ab.
35 exp acyclovir
36 ganciclovir/ or foscarnet/ or Idoxuridine/ or Trifluridine/
37 (ac?clovir or Zovirax or valac?clovir or valtrex or famc?clovir or famvir or penc?clovir or ganc?clovir or cidofovir or foscarnet$ or valganc?clovir or lubocavir or brivudin or Docosanol or Sorivudine or Idoxuridine or Trifluridine).ti,ab
38 or 1/37
39 exp dementia/
40 exp mild cognitive impairment/
41 (Alzheimer$ or dementia or Kluver-Bucy or huntington$).ti, ab.
42 (lewy$ adj2 bod$).ti, ab.
43 (Pick$ disease$ AND brain$).ti, ab.
44 ((memory or cognit$ or mental) adj5 (los$ or impair$ or deficit or problem or damage or declin$ or deteriorat$ or degenerat$ or diminish$)).ti, ab.
45 Supranuclear Palsy, Progressive/
46 (supra?nuclear pals$ or supra nuclear pals$ or PSP).ti, ab.
47 prion diseases/ or creutzfeldt-jakob syndrome/ or gerstmann-straussler-scheinker disease/ or insomnia, fatal familial/ or kuru/
48 (Prion$ disease$ or fatal familial insomnia or FFI or Gertsmann-Straussler-Scheinker syndrome or GSS or kuru or variab$ protease-sensitive prionopathy or VPSPr or transmissible spongiform encephalopath$ or TSE or Creutzfeld-Jacob$ or JCD or CJD).ti, ab.
49 or 39/48
50 38 and 49

Embase (OVID) search strategy [1947 to 2017 March 06]

1 exp herpes simplex/
2 exp herpes simplex virus/
3 herpes simplex vaccine/
4 cold sore$.ti,ab.
5 (genit$ herpes$ or genit$ sores).ti,ab.
6 Chickenpox/
7 Chickenpox vaccine/
8 exp herpes zoster/
9 exp Varicella zoster virus/
10 postherpetic neuralgia/
11 (varicella or chickenpox or chicken pox or shingles or VZV or zoster).ti,ab.
12 exp Human cytomegalovirus/
13 Cytomegalovirus vaccine/
14 exp Cytomegalovirus infection/
15 (CMV or cytomegalovirus).ti,ab.
16 Human herpesvirus 6/
17 Exanthema Subitum/
18 (B lymphotropic virus$ or roseola or sixth disease or exanthema subitum or exanthem criticum or Roseolovirus or pseudorubella or three?day fever).ti,ab.
19 Human herpesvirus 7/
20 exp Epstein-Barr virus infection/
21 Epstein-Barr virus/
22 Mononucleosis/
23 (EBV or epstein-barr or burkitt adj5 lymphoma$ or glandular fever or infectious mono$ or mononucleosis or hair$ leukoplak$ or OHL).ti,ab.
24 Herpesvirus 8, Human/
25 Kaposi sarcoma/
26 Primary effusion lymphoma/
27 (kaposi$ sarcoma$ or Primary effusion adj2 lymphoma$ or body cavity adj2 lymphoma$).ti,ab.
28 ((HHV adj1 ("1" or "2" or "3" or "4" or "5" or "6" or "7" or "8")) or (HHV?1 or HHV?2 or HHV?3 or HHV?4 or HHV?5 or HHV?6 or HHV?7 or HHV?8)).ti,ab.
29 (HSV?1 or HSV 1 or HSV?2 or HSV 2).ti,ab.
30 herpes$.ti, ab.
31 exp acyclovir/
32 ganciclovir/ or foscarnet/ or Idoxuridine/ or Trifluridine/
33 (ac?clovir or Zovirax or valac?clovir or valtrex or famc?clovir or famvir or penc?clovir or ganc?clovir or cidofovir or foscarnet$ or valganc?clovir or lubocavir or brivudin or Docosanol or Sorivudine or Idoxuridine or Trifluridine).ti,ab
34 or 1/33
35 exp dementia/
36 exp mild cognitive impairment/
37 (Alzheimer$ or dementia or Kluver-Bucy or huntington$).ti, ab.
38 (lewy$ adj2 bod$).ti, ab.
39 (Pick$ disease$ AND brain$).ti, ab.
40 ((memory or cognit$ or mental) adj5 (los$ or impair$ or deficit or problem or damage or declin$ or deteriorat$ or degenerat$ or diminish$)).ti, ab.
41 exp progressive supranuclear palsy/
42 (supra?nuclear pals$ or supra nuclear pals$ or PSP).ti, ab.
43 (Prion$ disease$ or fatal familial insomnia or FFI or Gertsmann-Straussler-Scheinker syndrome or GSS or kuru or variab$ protease-sensitive prionopathy or VPSPr or transmissible spongiform encephalopath$ or TSE or Creutzfeld-Jacob$ or JCD or CJD).ti, ab.
44 or 35/43
45 34 and 44

Global Health (OVID) search strategy [1910 to 2017 Week 08]

1 exp Human herpesviruses/
2 exp Human herpesvirus 1/
3 exp Human herpesvirus 2/
4 exp Herpes simplex viruses/
5 cold sore$.ti,ab.
6 (genit$ herpes$ or genit$ sores).ti,ab.
7 exp Varicella/
8 exp Human herpesvirus 3/
9 exp Herpes zoster/
10 (varicella or chickenpox or chicken pox or shingles or VZV or zoster).ti,ab.
11 exp Human herpesvirus 5/
12 (CMV or cytomegalovirus).ti,ab.
13 exp Human herpesvirus 6/
14 exp Roseolovirus/
15 Exanthema subitum/
16 (B lymphotropic virus$ or roseola or sixth disease or exanthema subitum or exanthem criticum or Roseolovirus or pseudorubella or three?day fever).ti,ab.
17 exp Human herpesvirus 4/
18 (EBV or epstein-barr or burkitt adj5 lymphoma$ or glandular fever or infectious mono$ or mononucleosis or hair$ leukoplak$ or OHL).ti,ab.
19 exp Human herpesvirus 8/
20 (kaposi$ sarcoma$ or Primary effusion adj2 lymphoma$ or body cavity adj2 lymphoma$).ti,ab.
21 Kaposi's sarcoma/
22 ((HHV adj1 ("1" or "2" or "3" or "4" or "5" or "6" or "7" or "8")) or (HHV?1 or HHV?2 or HHV?3 or HHV?4 or HHV?5 or HHV?6 or HHV?7 or HHV?8)).ti,ab.
23 (HSV?1 or HSV 1 or HSV?2 or HSV 2).ti,ab.
24 herpes$.ti, ab.
25 exp Aciclovir/
26 aciclovir/ or cidofovir/ or famciclovir/ or foscarnet sodium/ or ganciclovir/ or penciclovir/ or valaciclovir/
27 (ac?clovir or Zovirax or valac?clovir or valtrex or famc?clovir or famvir or penc?clovir or ganc?clovir or cidofovir or foscarnet$ or valganc?clovir or lubocavir or brivudin or Docosanol or Sorivudine or Idoxuridine or Trifluridine).ti,ab
28 or 1/27
29 exp dementia/
30 (Alzheimer$ or dementia or Kluver-Bucy or huntington$).ti, ab.
31 (lewy$ adj2 bod$).ti, ab.
32 (Pick$ disease$ AND brain$).ti, ab.
33 ((memory or cognit$ or mental) adj5 (los$ or impair$ or deficit or problem or damage or declin$ or deteriorat$ or degenerat$ or diminish$)).ti, ab.
34 (supra?nuclear pals$ or supra nuclear pals$ or PSP).ti, ab.
35 prion diseases/ or creutzfeldt-jakob disease/ or gerstmann-straussler-scheinker syndrome/ or kuru/
36 (Prion$ disease$ or fatal familial insomnia or FFI or Gertsmann-Straussler-Scheinker syndrome or GSS or kuru or variab$ protease-sensitive prionopathy or VPSPr or transmissible spongiform encephalopath$ or TSE or Creutzfeld-Jacob$ or JCD or CJD).ti, ab.
37 or 29/36
38 28 AND 37

PsycINFO (OVID) search strategy [1806 to February Week 4 2017]

1 exp herpes simplex/
2 exp herpes genitalis/
3 cold sore$.ti,ab.
4 (genit$ herpes$ or genit$ sores).ti,ab.
5 (varicella or chickenpox or chicken pox or shingles or VZV or zoster).ti,ab.
6 (CMV or cytomegalovirus).ti,ab.
7 (B lymphotropic virus$ or roseola or sixth disease or exanthema subitum or exanthem criticum or Roseolovirus or pseudorubella or three?day fever).ti,ab.
8 exp epstein barr viral disorder/
9 (EBV or epstein-barr or burkitt adj5 lymphoma$ or glandular fever or infectious mono$ or mononucleosis or hair$ leukoplak$ or OHL).ti,ab.
10 (kaposi$ sarcoma$ or Primary effusion adj2 lymphoma$ or body cavity adj2 lymphoma$).ti,ab.
11 ((HHV adj1 ("1" or "2" or "3" or "4" or "5" or "6" or "7" or "8")) or (HHV?1 or HHV?2 or HHV?3 or HHV?4 or HHV?5 or HHV?6 or HHV?7 or HHV?8)).ti,ab.
12 (HSV?1 or HSV 1 or HSV?2 or HSV 2).ti,ab.
13 herpes$.ti, ab.
14 exp antiviral drugs/
15 (ac?clovir or Zovirax or valac?clovir or valtrex or famc?clovir or famvir or penc?clovir or ganc?clovir or cidofovir or foscarnet$ or valganc?clovir or lubocavir or brivudin or Docosanol or Sorivudine or Idoxuridine or Trifluridine).ti,ab
16 or 1/15
17 exp dementia/
18 exp cognitive impairment/
19 (Alzheimer$ or dementia or Kluver-Bucy or huntington$).ti, ab.
20 (lewy$ adj2 bod$).ti, ab.
21 (Pick$ disease$ AND brain$).ti, ab.
22 ((memory or cognit$ or mental) adj5 (los$ or impair$ or deficit or problem or damage or declin$ or deteriorat$ or degenerat$ or diminish$)).ti, ab.
23 exp progressive supranuclear palsy/
24 (supra?nuclear pals$ or supra nuclear pals$ or PSP).ti, ab.
25 creutzfeldt jakob syndrome/
26 (Prion$ disease$ or fatal familial insomnia or FFI or Gertsmann-Straussler-Scheinker syndrome or GSS or kuru or variab$ protease-sensitive prionopathy or VPSPr or transmissible spongiform encephalopath$ or TSE or Creutzfeld-Jacob$ or JCD or CJD).ti, ab.
27 or 17/26
28 16 and 27

The Cochrane Library search strategy [inception to 7th March 2017]

1 (herpes*):ti,ab,kw
2 (((HHV NEAR/1 ("1" or "2" or "3" or "4" or "5" or "6" or "7" or "8")) or (HHV?1 or HHV?2 or HHV?3 or HHV?4 or HHV?5 or HHV?6 or HHV?7 or HHV?8))):ti,ab,kw
3 (HSV1 or "HSV 1" or HSV2 or "HSV 2"):ti,ab,kw
4 (ac?clovir or Zovirax or valac?clovir or valtrex or famc?clovir or famvir or penc?clovir or ganc?clovir or cidofovir or foscarnet* or valganc?clovir or lubocavir or brivudin or Docosanol or Sorivudine or Idoxuridine or Trifluridine):ti,ab,kw
5 (kaposi* sarcoma* or Primary effusion lymphoma* or body cavity lymphoma*):ti,ab,kw
6 (EBV or epstein-barr or burkitt NEAR/5 lymphoma* or glandular fever or infectious mono* or mononucleosis or hair* leukoplak* or OHL):ti,ab,kw
7 (B lymphotropic virus* or roseola or sixth disease or exanthema subitum or exanthem criticum or Roseolovirus or pseudorubella or three?day fever):ti,ab,kw
8 (CMV or cytomegalovirus):ti,ab,kw
9 (varicella or chickenpox or chicken pox or shingles or VZV or zoster):ti,ab,kw
10 ("genit* herpes*" or "genit* sores"):ti,ab,kw
11 (cold sore*):ti,ab,kw
12 #1 or #2 or #3 or #4 or #5 or #6 or #7 or #8 or #9 or #10 or #11
13 (Alzheimer* or dementia or Kluver-Bucy or huntington*):ti,ab,kw
14 (lewy* NEAR/2 bod*):ti,ab,kw
15 (Pick* disease* AND brain*):ti,ab,kw
16 ((memory or cognit* or mental) NEAR/5 (los* or impair* or deficit or problem or damage or declin* or deteriorat* or degenerat* or diminish*)):ti,ab,kw
17 (supra?nuclear pals* or supra nuclear pals* or PSP):ti,ab,kw
18 (Prion* disease* or fatal familial insomnia or FFI or Gertsmann-Straussler-Scheinker syndrome or GSS or kuru or variab* protease-sensitive prionopathy or VPSPr or transmissible spongiform encephalopath* or TSE or Creutzfeld-Jacob* or JCD or CJD):ti,ab,kw
19 #13 or #14 or #15 or #16 or #17 or #18
20 #12 and #19

SCOPUS (www.scopus.com) search strategy [inception to 7th March 2017]

((TITLE-ABS-KEY (alzheimer* OR dementia OR kluver-bucy OR huntington*)) OR (TITLE-ABS- KEY (lewy* W/2 bod*)) OR (TITLE-ABS-KEY (pick* AND disease* AND brain*)) OR (TITLE-ABS-KEY ((memory OR cognit* OR mental) W/5 (los* OR impair* OR deficit OR problem OR damage OR declin* OR deteriorat* OR degenerat* OR diminish*))) OR (TITLE-ABS-KEY (supra?nuclear AND pals* OR "supra nuclear pals*" OR psp)) OR (TITLE-ABS-KEY ("Prion* disease*" OR "fatal familial insomnia" OR ffi OR "Gertsmann-Straussler-Scheinker syndrome" OR gss OR kuru OR "variab* protease-sensitive prionopathy" OR vpspr OR "transmissible spongiform encephalopath*" OR tse OR creutzfeld-jacob* OR jcd OR cjd))) AND ((((TITLE-ABS-KEY ("cold sore*")) OR (TITLE-ABS-KEY ("genit* herpes*" OR "genit* sores")) OR (TITLE-ABS-KEY (varicella OR chickenpox OR "chicken pox" OR shingles OR vzv OR zoster)) OR (TITLE-ABS-KEY (cmv OR cytomegalovirus)) OR (TITLE-ABS-KEY ("B lymphotropic virus*" OR roseola OR "sixth disease" OR "exanthema subitum" OR "exanthem criticum" OR roseolovirus OR pseudorubella OR "three?day fever")) OR (TITLE-ABS-KEY (ebv OR epstein-barr OR "burkitt W/5 lymphoma*" OR "glandular fever" OR "infectious mono*" OR mononucleosis OR "hair* leukoplak*" OR ohl)) OR (TITLE-ABS-KEY ("kaposi* sarcoma*" OR "Primary effusion W/2 lymphoma*" OR "body cavity W/2 lymphoma*")) OR (TITLE-ABS-KEY ((hhv W/1 ("1" OR "2" OR "3" OR "4" OR "5" OR "6" OR "7" OR "8")) OR (hhv?1 OR hhv?2 OR hhv?3 OR hhv?4 OR hhv?5 OR hhv?6 OR hhv?7 OR hhv?8)))) OR (TITLE-ABS-KEY (hsv?1 OR "HSV 1" OR hsv?2 OR "HSV 2")) OR (TITLE-ABS-KEY (herpes*)) OR (TITLE-ABS-KEY (ac?clovir OR zovirax OR valac?clovir OR valtrex OR famc?clovir OR famvir OR penc?clovir OR ganc?clovir OR cidofovir OR foscarnet* OR valganc?clovir OR lubocavir OR brivudin OR docosanol OR sorivudine OR idoxuridine OR trifluridine))))

Web of Science [inception to 1st March 2017]

1 (("cold sore*")) OR (("genit* herpes*" or "genit* sores")) OR ((varicella or chickenpox or "chicken pox" or shingles or VZV or zoster)) OR ((CMV or cytomegalovirus)) OR (("B lymphotropic virus*" or roseola or "sixth disease" or "exanthema subitum" or "exanthem criticum" or Roseolovirus or pseudorubella or "three?day fever")) OR ((EBV or epstein-barr or burkitt NEAR/5 lymphoma* or "glandular fever" or "infectious mono*" or mononucleosis or "hair* leukoplak*" or OHL)) OR (("kaposi* sarcoma*" or "Primary effusion" NEAR/2 lymphoma* or "body cavity" NEAR/2 lymphoma*)) OR (((HHV NEAR/1 ("1" or "2" or "3" or "4" or "5" or "6" or "7" or "8")) or (HHV?1 or HHV?2 or HHV?3 or HHV?4 or HHV?5 or HHV?6 or HHV?7 or HHV?8))) OR ((herpes*)) OR ((ac?clovir or Zovirax or valac?clovir or valtrex or famc?clovir or famvir or penc?clovir or ganc?clovir or cidofovir or foscarnet* or valganc?clovir or lubocavir or brivudin or Docosanol or Sorivudine or Idoxuridine or Trifluridine))

2 ((Alzheimer* or dementia or Kluver-Bucy or huntington*)) OR ((lewy* NEAR/2 bod*)) OR (("Pick* disease*" AND brain*)) OR (((memory or cognit* or mental) NEAR/5 (los* or impair* or deficit or problem or damage or declin* or deteriorat* or degenerat* or diminish*))) OR (("supranuclear pals*" or "supra nuclear pals*" or PSP)) OR (("Prion* disease*" or "fatal familial insomnia" or FFI or "Gertsmann-Straussler-Scheinker syndrome" or GSS or kuru or "variab* protease-sensitive prionopathy" or VPSPr or "transmissible spongiform encephalopath*" or TSE or Creutzfeld-Jacob* or JCD or CJD))

3 #1 and #2

ClinicalTrials.gov search strategy [inception to 7th March 2017]

(herpes OR varicella OR zoster OR chickenpox OR cytomegalovirus OR epstein-barr OR "glandular fever" OR "infectious mononucleosis") AND (dementia OR Alzheimer OR "mild cognitive impairment")

Grey Literature Report (www.greylit.org) search strategy [inception to 7th March 2017]

Herpes dementia
Herpes alzheimer

British Library e-theses online service (http://ethos.bl.uk) search strategy [inception to 7th March 2017]

(herpes AND dementia)
(herpes AND alzheimer)

ii. Changes to the original protocol

1. We added a relevant bias domains to our risk of bias approach: Reverse causation was an additional domain not included in the protocol.
2. In the original protocol we said each domain would be classified as either 'high risk' (if criterion are very inadequately addressed), 'low risk' (if criterion are adequately addressed) or 'unclear risk'. We have now added an additional criterion - 'moderate risk' (if criterion are somewhat inadequately addressed).
3. Once the risk of bias assessment was completed, a summary risk of bias table was produced; where a domain had more than one item for assessment (e.g. the second domain, "selection of participants", had two items - participation bias and selection of controls) the highest risk of bias judgment was used in the summary table.
4. In the original protocol, we had written, "Studies were included if the primary outcome was stroke (first or subsequent)." However, if a study met all other criteria but stroke was only a secondary outcome, we decided it should be included. Therefore, we have revised it to, "Studies were included if stroke (first ever or subsequent) was an outcome".

iii. Extracted data items

Data items on the following five domains were extracted

1. *Population:* characteristics of the study population (e.g. mean/median age, ethnic distribution, immune status), inclusion and exclusion criteria;
2. *Exposure:* definition and identification of human herpesvirus exposure, number of exposed subjects;
3. *Comparators:* definition and identification of unexposed individuals, number of unexposed subjects;

4. *Outcomes:* definition and identification of primary (stroke) and secondary outcomes (stroke subtypes or TIA), number of subjects with outcome;
5. *Study characteristics:* authors, publication year, setting/source of participants, design, methods of recruitment and sampling, period of study, length of follow-up time (if relevant), aims and objectives.

iv. GRADE assessment of quality: down/ upgrading reasons

1. Risk of bias:

- Not serious if more than half the studies have no domain at high risk of bias.
- Very serious if studies with two or more domains at high risk of bias represent more than 50% of the total studies and contribute more than 50% to any meta-analyses.
- Serious if studies fall between not serious and very serious.

2. Inconsistency not serious if have 0, serious if have 1, and very serious if have two or more of the following:

- Heterogeneity is moderate (I^2 ~30-60%), substantial (50-90%) or considerable (75-100%).
- Wide variance of point estimates across studies.
- Minimal/no overlap of the confidence intervals.

3. Indirectness: Direct evidence consists of research that directly compares the interventions which we are interested in, delivered to the populations in which we are interested, and measures the outcomes important to patients. The effect on overall quality of evidence will vary depending on how indirect the evidence is. Rough guide - not serious if have 0, serious if have 1, and very serious if have two of the following:

- An indirect comparison (for example study A compares to a placebo and study B compares to a different drug).
- Studies differ in terms of population (e.g. be restricted to immunosuppressed patients).
- Studies differ in terms of exposure definition (e.g. for example use different methods to ascertain herpesvirus exposures).
- Studies differ in terms of outcome measures (e.g. for example be restricted to certain time-frames or have a different definition of stroke).

4. Imprecision: where studies have low power and consequently wide confidence intervals (based on guidelines from the Cochrane Back Group).

- Serious imprecision: Wide confidence intervals
- Very serious imprecision: Very wide confidence intervals

Upgrading reasons include

5. Large effect:

- None: most effect estimates are less than two.
- Strong association: ES* >2 or <0.5 (based on direct evidence, with no plausible confounders)
- Very strong association: ES* >4 or <0.2 (based on direct evidence with no serious problems with risk of bias or precision, i.e. with (sufficiently narrow confidence intervals)

6. Plausible confounding

- Would reduce demonstrated effect: e.g. If, for instance, only sicker patients receive an intervention or exposure, yet they still fare better, it is likely that the actual intervention or exposure effect is even larger than the data suggest (confounding by indication)

Patients given antivirals are likely to be less healthy so this could reduce the demonstrated effect seen in patients given antivirals.

- Would suggest spurious effect: When confounding is expected to increase the effect but no effect was observed.

7. Dose response gradient (yes/no)

etable 1: Study characteristics

Author, yr	Design	Study period	Setting	Study population at recruitment	Exposure definition and ascertainment	Comparator definition and ascertainment	Outcome type	Outcome definition and ascertainment
Human herpes simplex virus type 1 infection								
Beffert, 1998	Case control	Not reported	Country unknown, post-mortem brain samples.	Cases were AD brain samples and controls were non-AD brain samples.	HSV1 DNA: extracted from brain tissue and detected by PCR. Positivity defined as having at least 1 of 5 brain regions positive for HSV1.	HSV1 negative patients	AD	Neuropathologically confirmed
Bertrand, 1993	Case control	Not reported	Canada, brain tissue from the Douglas Hospital Brain Bank.	Cases were AD brain samples and controls were non-AD brain samples (neurological controls and ischaemic patients). Age range 56-95 years.	HSV1 DNA: detected by PCR. Positivity defined as having at least 1 of 5 brain regions positive for HSV1.	HSV1 negative patients	Sporadic AD	Neuropathologically confirmed
Cheon, 2001	Case control	Not reported	United Kingdom, brain samples from Medical Research Council's London Brain Bank for Neurodegenerative Diseases.	Cases were AD brain samples, age-matched to control brain samples from individuals with no neurological or psychiatric history.	HSV1 DNA: glycoprotein D gene (via PCR) and viral phosphoprotein and glycoprotein (via Western blotting). Mean time from death to brain dissection 30-40 d.	HSV1 negative patients	AD	Clinically assessed by psychiatrists during lifetime and met NINCDA/ADRDA criteria for probable AD.
Costa, 2017	Case control	Not reported	Italy, hospital in Milan	All paticipants were HSV-1 seropositive. Cases were patients with AD or MCI, age and gender matched to controls without AD.	Anti-HSV-1 IgG titers measured in diluted serum using commercial enzyme immunoassays. HSV-1 Ab titers were expressed as antibody index.	Levels of anti-HSV1 titres.	AD or MCI	Probable AD diagnosed according to NINCDA/ADRDA criteria and MCI by Petersen criteria. All participants had complete medical and neurological exam.
Deatly, 1990	Case control	Not reported	Canada, brain tissue from Dementia Study at University of Western Ontario.	Cases had AD and controls had no clinical or neuropathological evidence of neurological/ psychiatric disease or a non-AD dementing illness	HSV1 RNA: assessed by in-situ hybridization. And searched for in trigeminal ganglia and peripheral nervous system and viral RNA in the CNS.	People with no evidence of HSV DNA sequences	AD	Neuropathological examination: pathological lesions meeting criteria of the NIH and Dementia Study Laboratory.
Itabashi, 1997	Case control	Not reported	Japan, autopsy brain samples	Cases were patients with AD, age-matched to controls with no confirmed neuropsychiatric disease	HSV1 DNA: HSV glycoprotein D DNA (type I and II) detected by PCR with the technician blind to the diagnosis.	People with no evidence of HSV DNA sequences	AD	Diagnosis of AD was established by National Institute on Aging criteria.
Jamieson, 1991	Case control	Not reported	United Kingdom, autopsy brain samples.	Cases were AD brain samples and controls were brain samples from "normal" individuals.	HSV1 DNA: DNA sequences detected using PCR from brain specimens and lymphocytes.	People with no evidence of HSV DNA sequences	AD	Not reported
Jamieson, 1992a	Case control	Not reported	United Kingdom, blood samples from older individuals	Cases were patients with AD and controls were "normal" individuals.	HSV1 DNA: Peripheral blood lymphocytes examined using PCR.	People with no evidence of HSV DNA sequences	AD	Patients with cerebral atrophy who fulfilled the NINCDS-ADRDA criteria for "probable AD".
Jamieson, 1992b	Case control	Not reported	United Kingdom, post-mortem brain samples from two independent brain banks in Bristol and Edinburgh	Cases were patients with SDAT and elderly (age 64-95 years) controls were patients with no signs or a history of CNS disorder/neuropathological evidence of AD.	HSV1 DNA: PCR techniques to detect viral thymidine kinase gene in post-mortem brain tissue	People with no evidence of HSV DNA sequences	SDAT	Patients with SDAT had a history of dementia and the neuropathological features of AD
Kobayashi, 2013	Case control	Not reported	Japan, outpatients being treated at two hospitals	Cases had AD or aMCI: excluded if; diagnosed for >3 yrs, had dementia with Lewy bodies, frontotemporal /vascular dementia, normal pressure hydrocephalus, other CNS disease, head trauma, substance/major depressive/ psychotic disorder, epilepsy, delirium. Controls (age ≥60yrs) had no memory deficit or were patients with mood disorders.	HSV1 seropositivity and avidity: whole blood samples collected on day of cognitive function tests. Anti-HSV1 antibody titre measured using ELISA: seropositivity defined as IgG Index>1.0.	Patients without HSV1	AD and aMCI	AD diagnosis based on NINCDS–ADRDA criteria and MCI based on criteria defined by Peterson.
Lin, 1994	Case control	Not reported	United Kingdom, study using post-mortem brain tissue.	Cases were patients with AD and controls were "normal" individuals	HSV1 DNA: detected using PCR techniques, using two different primers.	People with no evidence of HSV DNA sequences	AD	Not reported
Lin, 1998	Case control	Not reported	United Kingdom, study using brain specimens.	Cases were patients with AD and controls were individuals without AD	HSV1 DNA: Detected using PCR and electrophoresis. Positive if one or both brain regions tested were positive.	People with no evidence of HSV DNA sequences	AD	DSM-IIIR and McKahn criteria (neurofibrillary tangles plus plaques, clinical history of dementia, no other explanation on brain examination).
Mancuso, 2014a (Frontiers in…)	Case control	Not reported	Italy, hospital in Milan	All subjects consecutively recruited. Cases had AD and controls had no family history of dementia or neurologic disease at enrolment.	HSV1 (IgG seropositivity and avidity): measured using commercial EIAs from serum (AI>1.1=positive, AI<0.09=negative). IgG avidity measured with protein-denaturing agent	Participants with no evidence of HSV1 IgG antibodies	AD or aMCI	Probable AD according to NINCDS–ADRDA criteria. aMCI according to Petersen criteria and Grundman operational criteria.
Mancuso, 2016	Case control	Not reported	Italy, setting unspecified	Cases were patients with AD or MCI and were matched to healthy controls on age and sex.	HSV1 IgG subclasses (IgG_1 and IgG_3): detected via ELISA from serum samples	Participants with no evidence of HSV1 IgG antibodies	AD or MCI	A diagnosis ofAD or MCI: no further information provided
Mori, 2004	Case control	Not reported	Japan, post-mortem brain samples	Cases were patients with AD and controls were without AD (either sudden death, schizophrenia, depression or manic depressive illness).	HSV1 DNA: Nested PCR detected the HSV1 glycoprotein D gene.	Participants with no evidence of HSV1 DNA	AD (familial and sporadic)	Familial AD - fulfilled recognized criteria for auto-somal dominant inheritance
Roberts, 1986	Case control	Not reported	United Kingdom, study using post-mortem brain tissue.	Cases were patients with AD and controls individuals without neurological conditions.	HSV antigenicity (an active infection): using the PAP method.	People with no evidence of HSV antigenicity	AD	Histologically diagnosed.
Wozniak, 2009	Case control	Not reported	United Kingdom, study using post-mortem brain tissue from the South West Dementia Brain Bank.	Cases with AD and controls (human glioblastoma (T98G) cells)	HSV1 DNA: by in situ PCR. Data from the frontal and cortex regions combined for each experimental subject	People with no evidence of HSV1	AD	Unclear
Varicella zoster virus reactivation (herpes zoster)								
Tsai, 2017	Cohort	2001-2008	Taiwan, Health Insurance Database	Individuals with herpes zoster opthalmicus (aged >40 years) propensity score matched to three patients without zoster. Patients with a history of dementia were excluded.	First ever herpes zoster opthalmicus in clinic or hospital, identified from ICD-9 codes.	Patients who had not received a diagnosis of herpes zoster within 1 year before the index date.	Dementia	Two or more dementia diagnoses by a certified neurologist
Human herpes simplex virus type 1 reactivation								
Helmer, 2015	Cohort	Not reported-	France, Three City French population-based cohort	Two samples of non-demented patients at baseline: 2341 participants with IgM at baseline and 3789 with reimbursement claims data after second follow-up	HSV IgM: Serological diagnosis at baseline sample	People with no evidence of HSV IgM at baseline	Dementia	DSM-IV criteria

etable 1: Study characteristics

Author, yr	Design	Study period	Setting	Study population at recruitment	Exposure definition and ascertainment	Comparator definition and ascertainment	Outcome type	Outcome definition and ascertainment
Epstein-Barr virus (EBV) infection								
Shim, 2017	Case control	2002-2010	Korea, population-based cohort study among elderly individuals, with follow-ups every 2-3 years.	Discovery sample: Cases were those converting from cognitively normal to aMCI over 2-yr follow-up period and had ≥2 consecutive follow-ups without reversion from aMCI to cognitively normal/AD. Matched controls (on age, sex and education) were cognitively normal over 2-yr follow-up period. Both drawn from 1,391 participants at baseline. Validation sample: converters only.	HSV1 and EBV IgG level IgG measured by ELISA before and after the "follow-up" period (for cases the follow-up point is the time they convert to aMCI).	Before or after converting/not converting to aMCI	amnestic MCI	Criteria included: memory complaints, objective memory impairment on a delayed recall test, relatively normal general cognitive function, normal/minimally impaired activities of daily living, and not demented.
Cytomegalovirus (CMV) infection								
Lovheim, 2015a	Case control (nested)	1988-2010	Sweden, The Northern Sweden Health and Disease Study Cohort with plasma samples and data from single hospital.	Cases had prior AD diagnosis and stored plasma samples available from original cohort study. Cases were matched (on age, sex) to dementia-free controls.	CMV IgG and IgM: Samples taken in original cohort study (1986-2006). Frozen samples thawed and analysed using ELISA techniques.	No serological evidence of infection	AD	Diagnosed at the University Hospital Memory Clinic in Umeå, Sweden, and validated before inclusion in the study
Kawasaki, 2016	Cohort	2008-2012	Japan, population-based cohort in Tokyo followed 3 yrs by annual telephone calls or mail survey	Individuals (age ≥85yrs) randomly selected from Tokyo (N=542) [excluded if had dementia or baseline MMSE score missing (N=50)] with 3yr follow-up.	CMV IgG seropositivity: measured by a quantitative EIA	Lowest quartile of CMV titre (presumably including those classed as 'negative')	Cognitive decline	Annual change of MMSE score
Renvoize, 1984	Case control	Not reported	United Kingdom, psychiatric hospitals in region of northern England.	Cases had AD and controls were non-AD patients with chronic functional psychoses. Cases with history of major psychiatric/physical disease, family history of other forms of dementia, CVD/focal neurological signs on examination or abnormal blood results excluded.	CMV antibody titres: using a complement-fixation test. Numbers with antibody titres ≥1/16 and mean antibody titres reported.	CMV antibody titre	AD	Patient examination and medical record review by psychiatrist: history of AD (before and after 65yrs for PDAT/SDAT, respectively) and slow, progressive cognitive impairment.
Westman, 2013	Case control	Not reported	Sweden, Memory Clinic at the Department of Geriatrics in Uppsala University Hospital and volunteers in similar geographic area.	Cases were patients with AD and controls were age-matched non-AD subjects (recruited from database of volunteers from same geographical area as cases).	CMV IgG seropositivity: using multi-colour flow cytometry, lymphocytes from peripheral blood mononuclear cells were analysed.	No serological/genetic evidence of infection	AD	Clinical AD diagnosis according to NINCDS-ADRDA and DSM-IV criteria, and CT/MRI scan consistent with AD.
Human herpesvirus 8 infection								
Dore, 1998	Cohort	1983-1994	Australia, prospective cohort study among patients with AIDS	Patients (age unspecified) diagnosed with AIDS at three major HIV//AIDS units. Information on initial and subsequent AIDS illnesses was collected.	KS: determined at recruitment and during follow-up, treated as a time-dependant covariate.	Patients without KS	ADC	Primary outcome: time to ADC from initial AIDS diagnosis.
Dupin, 2000	Case control	Not reported	France, AIDS patients; setting unknown	1960 AIDS patients; randomly selected cases with ADC and controls without ADC to have antibodies tested.	KS: immunofluorescence assay	Patients without KS	ADC	Not reported
Mary-Krause, 1999	Cohort	1992-1998	France, hospital database study on HIV patients	Patients with ≥6 mo follow-up or dying before 6 mo. Exclusion criteria: opportunistic CNS disease at entry, h/o opportunistic brain disease, patient in ART trial.	KS, modelled as a time-dependant covariate.	Person time without a diagnosis of KS.	HIV-related encephalopathy.	First occurrence of outcome
Polk, 2002	Case control (nested)	1984-1998	United States, Multicentre cohort study of homosexual men. Data collected via examinations, lab tests and interviews.	Cases were those developing dementia and were individually matched with replacement to controls on age, recruitment date and HIV characteristics.	HHV-8: immunofluorescence assay. No further HHV-8 testing if seropositive at entry. Otherwise, HHV-8 serostatus from visit closest to 1 yr pre-dementia diagnosis used.	No serological evidence of infection	Dementia	Progressive cognitive or behavioural decline according to third-party report or neurologic assessment with neuropsychological testing.
Renwick, 2001	Cohort study	1984-1996	The Netherlands, The Amerstam Cohort Studies on HIV infection and AIDS, a study pf HIV-1 infected homosexual men	Participants (age unknown) were HIV seropositive homosexual men.	HHV-8 antibodies: EIA. If tested positive, date of seroconversion determined by testing earlier samples, to time-update exposure.	No serological evidence of infection	Definite ADC	1987 CDC and Prevention surveillance case definition.
Rezza, 1999	Cohort study	1985-1997	Italy, multicentre cohort study of HIV seroconverters	Of 1520 cohort, selected if had exposure data 1yr (median) post-HIV seroconversion (n=390) and known HIV-exposure category (n=383, 25.4%).	HHV-8 antibodies: immunofluorescence assay	No serological evidence of infection	HIV encephalopathy	1987 CDC and Prevention surveillance case definition.
Treatment for herpesviruses (e.g. antivirals such as acyclovir)								
Gnann, 2015	RCT	2000-2009	Multinational (United States, Canada, England, and Sweden), double-blind RCT	Immunocompetent subjects (age ≥12 yrs) with HSE (HSV DNA in the CSF by PCR) who completed 14–21 days of intravenous acyclovir.	Valcyclovir: 2g daily (administered as four 500-mg tablets 3 times daily) for 90 dy	Identical placebo tablets for 90 days	Mild/no cognitive impairment at 12 months	Measured by MDRS and MMSE at 12 mo.
Multiple herpesviruses								
Agostini, 2016a	Cohort	Not reported	Italy, cohort of individuals with amnestic MCI followed by a Neurology department for 24 months	Patients (any age) with a diagnosis of amnestic MCI: those neurocognitive evaluation/serum samples at baseline and follow-up excluded.	HSV1, HHV6 IgG titres: commercial EIAs taken at baseline. Also measured HSV1 and HHV-6 avidity using a protein-denaturing agent.	No serological evidence of infection	AD	Neurocognitive evaluation, including MRI scan to identify those converting to AD
Agostini, 2016b	Case control	Not reported	Italy, a hospital providing healthcare rehabilitation, social welfare and social therapeutic services.	Cases were patients with AD or aMCI, age-matched to healthy controls who were unrelated healthy spouses of AD and MCI patients with MMSE score >28.	HHV-6, CMV and HSV1 IgG antibody titres and avidity index: using ELISA and protein-denaturing agent.	Patients without HHV-6, CMV, HSV1 IgG antibody titres or low avidity index	AD and aMCI	AD: NINCDS-ADRDA and aMCI: Petersen and Grundman criteria. AD patients were mild as determined by CDR scale and MMSE score
Aiello, 2006	Cohort	1998-2003	United States, community-dwelling Mexican Americans, sampled from existing cohort study	Adults (aged 60-100yrs) in SALSA cohort study with available blood samples and ≥2 sequential follow-up visits (67%; 1,204/1,789 from original cohort study).	CMV and HSV1 IgG: using ELISA, serum samples taken at baseline. Antibody levels treated as continuous term and split into quartiles.	Lowest quartile of antibody level	Cognitive decline	MMSE and DEL-REC score assessed at baseline and annually over 4-yrs.
Bu, 2015	Case control	2012-2013	China, single hospital and mental health centre	Cases were consecutive AD patients matched (on age, gender) to controls with normal cognition, randomly recruited from hospitals health examination centre.	CMV, HSV1 IgG antibody titres: ELISA techniques. All measurements performed in a blinded manner to clinical outcomes.	No serological evidence of infection	AD	AD diagnosed clinically in line with NINCDS-ADRDA criteria.

etable 1: Study characteristics

Author, yr	Design	Study period	Setting	Study population at recruitment	Exposure definition and ascertainment	Comparator definition and ascertainment	Outcome type	Outcome definition and ascertainment
Barnes, 2015	Cohort	1994-2012	United States, 3 ongoing cohort studies (Rush Memory and Aging Project (MAP), the Religious Orders Study (ROS), and the Minority Aging Research Study (MARS)): annual clinical evaluations, cognitive testing, blood sample and neurological examination.	All participants dementia free at entry, had blood samples and ≥2 cognitive tests to assess decline. Stratified sampling scheme: black individuals from all cohorts included (n=210) and white participants (n=639) randomly selected from MAP and ROS.	CMV and HSV1 IgG: CMV detected using solid-phase ELISAs and assays of antibodies with specificity for HSV1 performed using purified viral envelope glycoprotein gG-1 as the solid-phase antigen. Seronegative= immune status ratio<0.9, seropositive=≥0.9 or equivocal values.	No serological evidence of infection	AD	Annual evaluation by an experienced clinician according to NINCDS–ADRDA criteria.
Carbone, 2014	Case control (nested)	1999-2004	Italy, individuals originally recruited into a longitudinal "Conselice study of brain ageing"	From original cohort of 1016 adults (≥65 years), 985 had blood samples taken. All cases and controls were AD free at the strat of the study: cases had AD after 5 years of follow-up and controls had no AD at follow-up.	CMV, EBV or HHV-6 IgG and DNA: IgG from peripheral blood leukocytes using ELISAs. DNA from blood using PCR. All samples taken at basline.	No serological/genetic evidence of infection	AD	Cases and controls clinically diagnosed according to DSM IV and NINCDS-ADRDA.
Deng, 2016	Case control	2006-2014	China, employees of the Kailuan Group Company aged 50-65 yrs and with no CVD at enrollment	Cases had caregiver >4dys/wk and diagnosis of cognitive impairment in 2008-2014. Controls were admitted to hospital with no cognitive impairment.	Serological evidence of HSV1, HSV-2 or CMV	No serological evidence of infection	Vascular cognitive impairment with no dementia (VCIND).	Clinical MCI diagnosis, MMSE >20/>24 for those with elementary/ secondary education, appropriate CDR results and MRI findings.
Hemling, 2003	Case control	Not reported	Finland, post-mortem brain specimens	Cases were AD patients and controls were patients with no clinical or neuropathological evidence of neurological or psychiatric disease.	HSV1, HHV-6, VZV DNA: Detected by PCR from brain specimens. DNA-positive if any of the neuroanatomical regions gave positive result	No evidence of herpesvirus DNA	Lates-onset AD	Clinically diagnosed and neuropathaolgically confirmed.
Itzhaki, 2005	Case control	Not reported	Not reported	Cases were VaD patients and controls were "elderly normals"	HSV1, HHV-6, CMV DNA: no further information.	No evidence of herpesvirus DNA	VaD	No information
Kittur, 1992	Case control	Not reported	United States, dementia clinic in single hospital, Baltimore Longitudinal Study of Aging, brain specimens from autopsy.	Cases were AD patients from dementia clinic and age-matched controls (without dementia or neurologic symptoms) came from Baltimore study.	HSV1, HSV2, EBV, CMV DNA from blood and brain tissues.	No evidence of herpesvirus DNA	AD	Diagnosed according to consensus clinical diagnostic criteria.
Letenneur, 2008	Cohort	1988-2002	France, PAQUID cohort study of cerebral aging. Data collected through series of surveys and clinical assessments.	Individuals (≥65 yr) from cohort (N=3777) with serum samples from 1st visit (N=591). Excluded if had dementia at time of blood sample collection (n=28) and patients died/were lost to follow-up (n=51).	HSV1/2 IgM and IgG antibodies: from frozen samples taken in 1989 using ELISAs.	No serological evidence of infection	AD	NINCDS/ADRDA and DSMIII-R criteria (evaluated by psychologists), then confirmed by neurologist.
Lin, 1996	Case control	Not reported	United Kingdom, brain specimens following post-mortem	Cases were brain from patients with AD and controls were "normals"	HSV1 and VZV DNA: PCR. DNA from 1-3 brain regions extracted; if all brain regions negative, patients classed as negative for herpesvirus.	People with no evidence of HSV1 or VZV DNA	AD	Not defined
Lin, 2002a (J of Pathology)	Case control	Not reported	United Kingdom, brain specimens.	Cases were patients with AD and they were age-matched to "normals"	HSV1, HSV2, CMV, HHV6 DNA Brain tissue, usually frontal and temporal cortex, was examined for DNA using PCR.	No evidence of DNA	AD	No information
Lin, 2002b	Case control	Not reported	United Kingdom, frozen post-mortem brain specimens	Cases were patients with VaD or Binswanger's disease and controls were age-matched normals without neurological disease.	CMV, HHV6 and HSV1 DNA: PCR. Virus-positive if ≥1 region positive and virus-negative if all regions negative. HSV1 only measured in VaD cases, not controls.	No evidence of DNA sequences	VaD	No information
Lovheim, 2015b	Case control (nested)	1986-2010	Sweden, The Northern Sweden Health and Disease Study Cohort and linked hospital records	Cases had AD diagnosis with stored plasma samples, from original cohort study. Cases included if an age, sex, cohort and sampling date-matched, dementia-free control available.	HSV1/2 IgG and IgM antibodies: ELISA. Samples taken as part of original cohort study (between 1986-2006).	No serological evidence of infection	AD	Medical record review for clinical diagnoses in hospital: assessment of symptoms, physical examinations, cognitive tests and brain imaging.
Lovheim, 2015c	Cohort	1988-2010	Sweden, longitudinal study of persons from single area (to investigate memory and health) linked to death data.	All participants (aged ≥60 years) contributing at least one sample or at least two samples from different examinations.	HSV1/2 IgG and IgM antibodies: ELISA.	No serological evidence of infection	AD	Clinical evaluation and evaluation of medical records: research psychiatrist then assessed those with cognitive deviation to determine diagnosis.
Lycke, 1974	Case control	Not reported	Sweden, a hospital for acute and chronic psychiatric disorders. Source of reference controls not clear.	Cases were selected from hospital of psychiatric disorder and healthy individuals donating blood of varying ages were used as controls.	HSV1/2, CMV, VZV antibodies: complement fixation method.	No serological evidence of infection	Senile and atherosclerotic dementia	No information
Mancuso, 2014b	Case control	Not reported	Italy, dementia clinic of hospital in Milan	Cases were AD patients matched (age, gender) to healthy controls, with no family history of dementia, no evidence of neurologic disease and MMSE≥28	HSV1 and CMV antibodies: Serum IgG titres measured using commercial EIAs.	No serological evidence of infection	AD	NINCDS-ADRDA and National Institute on Aging criteria. Patients had mild AD (MMSE range 18–23).
Mann, 1981	Case control	Not reported	Country not reported, brain samples of patients - setting unknown.	Cases had either died with AD or had AD diagnosed following biopsies of temporal lobe where tumour suspected and controls (of similar age) had no neurological/psychiatric disease or had undergone craniotomy where tumour of temporal lobe suspected.	HSV antibodies: unlabelled antibody-enzyme PAP method.	No serological evidence of infection	AD	Either 1) diagnosis of AD and histological evidence AD; or 2) AD diagnosed following craniotomy- exact definition of AD not defined for second group.
Marques, 2001	Case control	Not reported	United States, study using post-mortem brain tissue.	Cases were AD brain samples and controls were non-AD brain samples. Autopsies performed maximum of 19 h post-mortem.	HSV1 and HSV2 DNA: using the DELFIA time resolved fluorescence hybridization assay.	Participants with no evidence of HSV1 or HSV2 DNA	AD	Pathological diagnosis according to the Consortium to Establish a Registry for Alzheimer's Disease
Ounanian, 1990	Case control	Not reported	France, unclear.	Cases were patient with AD and controls were age-matched subjects presenting no neurological or immunological diseases.	HSV1, CMV, VZV IgG Ab: ELISA tests. Ab to EBV nuclear antigens (EBNA), to early antigens (EA) and viral vapsid antigens (VCA) detected using immunofluorescence.	No serological evidence of infection	Sporadic AD	Diagnosed according to the DSM-III definition.

etable 1 Study characteristics

Author, yr	Design	Study period	Setting	Study population at recruitment	Exposure definition and ascertainment	Comparator definition and ascertainment	Outcome type	Outcome definition and ascertainment
Renvoize, 1987	Case control	Not reported	United Kingdom, hospital based study	Cases had AD and controls were patients without AD suffering from functional psychiatric disorders, newly admitted to a single hospital for assessment.	Serum antibody titres to HSV and CMV: complement fixation. Comparison of mean titres	Comparison of mean titres	AD	Clinical diagnosis, Hachinski score of ≤4 and Mental Test score of <18.
Strandberg, 2003	Cohort	2000-2003	Finland, random sample home-dwelling older individuals with CVD recruited into DEBATE cohort study	All participants in DEBATE study, exlcuding 17 (4%) without antibody data at baseline and 35 (9%) who died or missing cognitive data 1 yr after cohort entry.	CMV, HSV1 or HSV2 IgG Ab: EIA method from baseline samples. Number of seropositivities toward HSV1, HSV2, CMV: 3 categories (0-1, 2, 3).	Patients with 0-1 viral seropositives.	Cognitive decline	Decrease of MMSE score (baseline points minus points at 1 yr). All tests performed by same trained nurse.
Taylor, 1986	Case control	Not reported	United Kingdom, post-mortem brain tissues from two centres collected and frozen within 48 hours of death	Cases were individuals with schizophrenia, AD or Huntington's Disease and controls were individuals without neurological conditions.	CMV and HSV1 DNA sequences: molecular hybridization	People with no evidence of HSV1 or CMV sequences	AD	Diagnosed by presence of neurofibrillary tangles and plaques on neuropathological examination.
Westman, 2017	Case control	Not reported	Sweden, Memory Clinic single hospital and volunteers in similar geographic area.	Cases were patients with AD and controls were age-matched non-AD subjects recruited from a database of listed volunteers from the same geographical area.	HSV1, CMV, VZV, HHV-6A: multiplex immunoassay for IgG reactivity toward each virus. HHV6 DNA levels analysed using PCR in PBMCs.	No serological/genetic evidence of infection	AD	Clinical AD diagnosis in line with the NINCDS-ADRDA and DSM-IV criteria and CT/MRI scan consistent with AD
Wozniak, 2005	Case control	Not reported	UK and USA, four brain banks	Cases were patients with AD from the four brain banks and controls were age-matched "normals" from two brain banks.	AI for HSV1 and HHV6 measured using indirect ELISA for IgG antibody. AI≥1.5=intrathecal antibody synthesis. Specimens taken post-mortem for some AD patients; all other specimens taken whilst patients alive.	Subjects with no evidence of intrathecal antibody synthesis: an AI<1.5	AD	AD diagnosed differently in four brain banks: Two banks used the NINCDS-ADRDA definition, one the DSM definition and one a consortium definition.

Abbreviations: RCT=randomised controlled trial, SCCS=self-controlled case series, RR=risk (or rate) ratio, CI=confidence interval, AI=antibody index, KS=Kaposi's sarcoma, aMCI=amnestic mild cognitive impairment, ELISA=, NINCDS-ADRDA=National Institute of Neurological and Communicative Diseases and Stroke/AD and Related Disorders Association, MMSE=Mini-Mental State Examination, HADS=Hamilton Depression Rating Scale, VaD=Vascular dementia, FAB=frontal assessment battery, AI=antibody index, NIH=National Institute of Health, SDAT=Senile dementia of the AD type, CNS=Central nervous system, AI=avidity index, GMT=geometric mean titre, MDRS=Mattis Dementia Rating Scale, EIA=enzyme immunoassay

etable 2: Study results

Author, yr	Design	Population size (N), follow-up time (years)	Subjects with outcome [or exposure for case-control studies] (N, %)	Statistical analysis method used	Main reported results	Adjusted for
Human herpes simplex virus type 1 infection						
Beffert, 1998	Case control	AD Cases: n=73<br>Controls: n=33	AD cases: 54/73 (74%)<br>Controls: 24/33 (73%)	Chi-squared test	OR 1.07 (95% CI 0.42-2.69)<br>*[calculated by review authors]*	None
Bertrand, 1993	Case control	AD Cases: n=98<br>Controls: n=57	AD cases: 73/98 (74.5%)<br>Controls: 41/57 (71.9%)	Chi-squared test	OR 1.14 (95% CI 0.55-2.38)<br>*[calculated by review authors]*	None
Cheon, 2001	Case control	AD Cases: n=10<br>Controls: n=10	HSV1 glycoprotein D gene, viral phosphoprotein and glycoprotein detected in all samples examined.	None performed	OR 1.00 (95% CI 0.02-55.27)<br>*[calculated by review authors]*	Matching factors only (age)
Costa, 2017	Case control	AD Cases: n=79<br>MCI cases: n=57<br>Controls: n=81	AD Cases: mean±SD: 8.80±2.39 AI<br>MCI cases: mean±SD: 8.55±2.44 AI<br>Controls: mean±SD: 7.93±2.43 AI, p=0.037	Binomial logistic regression	AD versus controls: OR: 1.22 (95%CI: 1.04–1.43)<br>MCI versus controls: P=0.194<br>AD versus MCI: P=0.577	Age, sex, and APOE4 positivity/negativity,
Deatly, 1990	Case control	AD cases: n=21<br>Controls: n=19	HSV1 RNA<br>AD cases:17/21 (81.0%)<br>Controls: n=9/19 (47.4%)	Chi-square test	OR 4.72 (95% CI 1.15-19.41)<br>*[calculated by review authors]*	None
Itabashi, 1997	Case control	AD cases: n=46<br>Controls: n=23	AD cases: 14/46 (30·4%)<br>Controls: 5/23 (21·7%)	None performed	OR 1.58 (95% CI 0.49-5.09)<br>*[calculated by review authors]*	Matching factors only (age)
Jamieson, 1991	Case control	AD Cases: n=8<br>Controls: n=5	HSV DNA detected in all brain samples but no lymphocyte samples.	None performed	HSV DNA in brain samples: 1.55 (95% CI 0.03-89.96)<br>HSV DNA in lymphocyte samples: OR 0.65 (95% CI 0.01-37.7)<br>[calculated by review authors]	None
Jamieson, 1992a	Case control	AD Cases: n=6<br>Controls: n=5	HSV DNA detected in zero lymphocyte samples.	None performed	OR 0.85 (95% CI 0.01-50.1)<br>[calculated by review authors]	None
Jamieson, 1992b	Case control	SDAT cases: n=21<br>Controls: n=15	SDAT cases: 14/21 (66.7%)<br>Controls: 9/15 (60.0%)	None performed	OR: 1.33 (95% CI 0.34-5.27)<br>*[calculated by review authors]*	None
Kobayashi, 2013	Case control	AD Cases: n=85<br>aMCI cases: n=34<br>Controls: n=28	AD Cases: n=66/85 (77.6%)<br>aMCI cases: n=29/34 (85.3%)<br>Controls: n=17/28 (60.7%)	Chi-squared test for seropositivity analyses and Mann– Whitney U test for avidity analysis.	Anti-HSV1 antibody avidity index<br>Higher in aMCI group than in healthy control and AD groups (P < 0.05, P < 0.05).<br>Seropositivity (baseline: controls)<br>AD cases: OR 2.25 (95% CI 0.90-5.61)<br>aMCI cases: OR 3.75 (95% CI 1.11-12.65)<br>[calculated by review authors]	None (though there was no association between age and HSV1 avidity).
Lin, 1994	Case control	AD Cases: n=25<br>Controls: n=22	AD cases: n=17/25 (68%)<br>Controls: n=14/22 (64%)	None performed	OR: 1.21 (95% CI 0.36-4.07)<br>*[calculated by review authors]*	None
Lin, 1998	Case control	AD cases: n=61<br>Controls: n=48	AD cases: n=45/61 (73.8%)<br>Controls: n=30/48 (62.5%)	Logistic regression	OR: 1.69 (95% CI 0.75-3.82)<br>*[calculated by review authors]*	None
Mancuso, 2014a (Frontiers in…)	Case control	AD cases: n=83<br>aMCI cases: n=68<br>Controls: n=74	HSV1 seroprevalence: AD cases: n=81/83 (97.6%), aMCI cases: n=65/68 (95.6%), Controls: n=73/74 (98.6%)<br>HSV1 IgG levels (AI) AD: median: 9.3 AI; range: 7.4–10.6 AI , aMCI: median: 8.8 AI; range: 7.1–10.5 AI , Controls: median: 7.9 AI; range: 6.0–9.6 AI<br>HSV1 IgG Avidity Index,% (median, IQR) AD cases: 90.8 [84.8–99.3], aMCI cases: 91.6 [86.0–96.2], Controls: 89.2 (85.6–94.6)	Chi-squared tests and ANOVA models	HSV1 seroprevalence: AD: OR 0.55 (95% CI 0.05-6.25), aMCI: OR 0.30 (95% Ci 0.03-2.92) *[calculated by review authors]*<br>HSV1 IgG levels (AI): HC vs. AD p=0.0049; HC vs. aMCI p=0.025<br>HSV1 IgG Avidity Index,%: no P values reported	None
Mancuso, 2016	Case control	AD cases: n=28<br>MCI cases: n=28<br>Controls: n=27	IgG_1 AD cases: n=28/28 (100%), MCI cases: n=28/28 (100%), Controls: n=23/27 (85%)<br>IgG_3 AD cases: n=18/28 (64%), MCI cases: n=26/28 (93%), Controls: n=20/27 (74%)	None performed	IgG1<br>AD: OR 10.91 (95% CI 0.55-2.13.26)<br>MCI: OR 10.91 (95% CI 0.55-2.13.26)<br>IgG3:<br>AD: OR 0.63 (95% CI 0.20-2.00)<br>MCI: OR 4.55 (95% CI 0.85-24.32)<br>*[calculated by review authors]*	Matching factors only (age and sex)
Mori, 2004	Case control	Familial AD cases: n=3<br>Sporadic AD cases: n=2<br>Controls: n=6	HSV1 detected by PCR<br>Familial AD cases: n=3/3 (100%)<br>Sporadic AD cases: n=2/2 (100%)<br>Controls: n=1/6 (16.7%)<br>HSV1 detected in frontal and temporal lobe of familial AD cases and frontal lobe of sporadic AD cases and control.	Fisher's exact test	(combining AD cases...)<br>OR 40.33 (95% CI 1.33-1223.05)<br>[calculated by review authors]	None
Roberts, 1986	Case control	AD cases: n=25<br>Controls: n=32	No Herpes simplex virus antigenicity was seen in any temporal lobe area in the AD cases or the controls.	None performed	OR 1.27 (95% CI 0.02-66.47)<br>[calculated by review authors]	None
Wozniak, 2009	Case control	AD cases: n=6<br>Controls: n=5	AD cases: 6/6 (100%)<br>Controls: 5/5 (100%)	Binomial regression	OR 1.18 (95% CI 0.02-70.0)<br>*[calculated by review authors]*	None
Varicella zoster virus reactivation (herpes zoster)						
Tsai, 2017	Cohort	Herpes zoster opthalmicus patients: n=846<br>Comparison patients: n=2538<br>Maximum follow-up 5 years	Herpes zoster opthalmicus patients: n=39/846 (4.6%)<br>Comparison patients: n=42/2538 (1.7%)	Cox proportional hazards regression	HR: 2.97 (95% CI 1.90-4.67)	Sex, age, urbanization level and geographic location, monthly income, hypertension, diabetes, hyperlipidemia, CHD and stroke
Human herpes simplex virus type 1 reactivation						
Helmer, 2015	Cohort	Exposed: 75/2341 (3.2%)<br>Unexposed: 2266/2341 (96.8%)<br>Follow-up: maximum 12 yrs	513 incident cases of dementia: unclear how many exposed/unexposed.	Cox models	By follow-up time (CIs calculated by review authors):<br>Entire follow-up: HR 1.1 (95% CI 0.53-2.28)<br>At/after 7 yr follow-up: HR 1.4, (95% CI 0.74-2.67)<br>At/after 10 yr follow-up: HR 2.1 (1.07-4.11)	Age, sex, APOE4, CVD risk factors
Epstein-Barr virus (EBV) infection						

etable 2: Study results

Author, yr	Design	Population size (N), follow-up time (years)	Subjects with outcome [or exposure for case-control studies] (N, %)	Statistical analysis method used	Main reported results	Adjusted for
Shim, 2017	Case control	"Discovery" sample:<br>Cases: n=18<br>Controls: n=18<br>Validation sample:<br>Cases: n=65		Paired t-test or Wilcoxon test	Discovery sample (mean (SD))<br>Anti-HSV1 IgG<br>Cases: pre-FU: 8.4 (2.4), post-FU: 8.4 (2.4) P=0.50, Controls: pre-FU: 9.0 (1.8) post-FU 9.1 (1.8) P=0.58<br>Anti-EBV IgG<br>Cases: pre-FU: 69.0 (18.9), post-FU: 71.3 (17.2) P=0.003, Controls: pre-FU: 58.9 (23.9), post-FU: 59.1 (24.6) P=0.65	Matching factors only.
Cytomegalovirus (CMV) infection						
Lovheim, 2015a	Case control (nested)	AD cases: n=360<br>Controls: n=360	IgG positive: Cases: 312/360 (86.7%), Controls: n=318/360 (88.3%)<br>No results for IgM	Not reported	IgG: OR 0.86 (95% CI 0.55-1.34)	Matching factors only (age and sex)
Kawasaki, 2016	Cohort	N=328 (2 patients with severe cognitive impairment excluded)	Unclear	Linear regression	Effect estimate is -0.00 (95% CI -0.35 - 0.35) for Q4 CMV versus Q1 CMV titre	Age, sex, education, baseline MMSE category
Renvoize, 1984	Case control	AD cases: n=113<br>Controls: n=39	CMV antibody titres ≥ 1/16<br>AD cases: 97/113 (85.8%), Controls: 30/39 (76.9%)<br>Mean antibody titre ($\log_{10}$) to CMV<br>AD cases: 32, $\log_{10}$ 1.5051 ± 0.3709, Controls: 27.27, $\log_{10}$ 1.4357 ± 0.3878	Chi-squared test	CMV antibody titres ≥ 1/16: OR 1.82 (95% CI 0.73-4.53)<br>Mean antibody titre ($\log_{10}$) to CMV: t = 0.9963, 0.5>P>0.1	None reported
Westman, 2013	Case control	AD cases: n=50<br>Controls: n=50	CMV IgG<br>AD cases: n=42/50 (84%)<br>Controls: n=39/52 (78%)	Non-parametric Mann-Whitney U-test	IgG: OR 1.48 (95% CI 0.54-4.06)	Matching factors only (age)
Human herpesvirus 8 infection						
Dore, 1998	Cohort	KS exposed: n=678<br>Unexposed: n=1760	KS exposed: n=29/678 (4.3%)<br>Unexposed: 169/1760 (9.6%)	Cox proportional hazard models	RR 0.48 (95% CI 0.32-0.71)	CD4 count at initial AIDS illness
Dupin, 2000	Case control	ADC cases: n=75<br>Controls: n=78	ADC cases: 15 out of 75 (20%)<br>Controls: 17/78 (21.8%)	Chi-squared test	OR 0.90 (95 % CI: 0.41 to 1.96) *[calculated by review authors]*<br>GMT of KSHV antibodies higher in patients without ADC than patients with ADC (P = 0.03)	
Mary-Krause, 1999	Cohort	9117 patients (median follow-up 15 months (IQR 8-26 months)	Not given	Cox proportional hazards model	HR 0.78 (95% CI0.62-0.96)	Age at AIDS diagnosis, exposure group and CD4 cell counts at AIDS diagnosis
Polk, 2002	Case control (nested)	Dementia cases: n=210<br>Controls: n=210	HHV-8 positive<br>Cases: 142/210 (67.6%)<br>Controls: 136/210 (64.8%)	Conditional logistic regression	OR 1.22 (95% CI 0.66, 2.28)	Matching factors only
Renwick, 2001	Cohort study	Overall: n=599<br>HHV-8 exposed: n=290 (48.4%)<br>Unexposed: n=309 (51.6%)	HHV-8 positive: n=14/290 (4.8%)<br>HHV-8 negative: n=16/309 (5.2%)	Cox proportional hazards	HR 0.70 (0.29-1.87)	ART use, CD4 cell count
Rezza, 1999	Cohort study	Overall: n=386<br>Exposed: n=147 (38.1%)<br>Unexposed: n=239 (61.9%)	HHV-8 positive: n=8/147 (5.4%)<br>HHV-8 negative: n=4/239 (1.7%)	Cox proportional models	HR 1.00 (95% CI 0.26-3.79)	Age at HIV seroconversion, gender, HIV exposure category, time dependent ART and CD4 cell count
Treatment for herpesviruses (e.g. antivirals such as acyclovir)						
Gnann, 2015	RCT	Intervention group: n=40<br>Placebo group: n=47	Mattis dementia scale: Intervention group: n=30/36 (85.7%), Placebo group: n=37/43 (90.2%)<br>MMSE scale: Intervention group: n=29/36 (87.9%), Placebo group: n=30/43 (85.7%)	Chi-squared test	Mattis dementia scale: OR 0.81 (95% CI 0.24-2.77)<br>MMSE: OR 1.80 (95% CI 0.63-5.14)<br>*[calculated by review authors]*	
Multiple herpesviruses						
Agostini, 2016a	Cohort	HSV1 Exposed: n=35 (97%)<br>Unexposed: n=1 (3%)<br>HHV-6 Exposed: n=31<br>Unexposed: n=5<br>Follow-up: 24 months	HSV1 seropositivity: Exposed: n=21/35 (60.0%), Unexposed: 0/1 (0%)<br>HHV-6 seropositivity: Exposed: 18/31 (58.1%), Unexposed: 3/5 (60.0%)	Chi-square test, t Student's test, Kruskal-Wallis test and the Mann Whitney U test	HSV1 seropositivity: RR 2.39 (95% CI 0.21-26.74)<br>HHV-6 seropositivity: RR 0.97 (0.45-2.10)<br>[calculated by review authors]	None
Agostini, 2016b	Case control	AD cases: n=59<br>aMCI cases: n=60<br>Controls: n=61	AD patients<br>HHV-6 IgG: 47/59 (79.7%), IgG avidity: 100% (94.3–100)<br>HSV1 IgG: 57/59 (96.6%), IgG avidity: 90.8% (84.6–99.6)<br>CMV IgG: 55/59 (92.7%), IgG avidity: 99.5% (88.1–100)<br>aMCI patients<br>HHV-6 IgG:47/60 (78.3%), IgG avidity: 100% (85.3–100)<br>HSV1 IgG: 58/60 (96.5%), IgG avidity: 91.6% (86.1–96.0)<br>CMV IgG: 58/60 (96.5%), IgG avidity: 100% (84.6–100)<br>Controls<br>HHV-6 IgG: 56/61 (75.4%), IgG avidity: 100% (95.7–100)<br>HSV1 IgG: 60/61 (98.0%), IgG avidity: 89.5% (85.6–94.4)<br>CMV IgG: 59/61 (97.1%), IgG avidity: 87.9% (70.5–100)	Kruskal-Wallis test and, when appropriate, the Mann Whitney U test, and the correlations using Spearman's correlation coefficient	Higher HSV1 titres are seen in AD and aMCI individuals compared to HC (AD versus HC: p=0.0001; aMCI versus HC: p=0.002).<br>HHV-6 seropositive<br>AD: OR 0.35 (95% CI 0.11-1.06), aMCI: OR 0.32 (95% CI 0.11-0.97)<br>HSV1 seropositive<br>AD: OR 0.48 (95% CI 0.04-5.38), aMCI: OR 0.48 (95% CI 0.04-5.48)<br>CMV seropositive<br>AD: OR 0.47 (95% CI 0.08-2.65), aMCI: OR 0.98 (95% CI 0.13-7.21)<br>*[calculated by review authors]*	Matching on age only.

etable 2: Study results

Author, yr	Design	Population size (N), follow-up time (years)	Subjects with outcome [or exposure for case-control studies] (N, %)	Statistical analysis method used	Main reported results	Adjusted for
Aiello, 2006	Cohort	HSV1<br>Exposed: n=1163/1204 (96.6%)<br>Unexposed: 41/1204 (3.4%)<br>CMV<br>Exposed: n=1182/1204 (98.2%)<br>Unexposed: 22/1204 (1.8%)<br>Follow-up time: max 4 years.	No cross-tabulations for exposure and outcome.	Mixed regression models treating cognitive decline as a continuous variable	DECLINE IN MMSE SCORE<br>CMV:<br>One-unit increase in IgG antibody level: β=-0.274, P<.001<br>Highest antibody levels compared with the lowest levels (β=-0.053, P=0.003).<br><br>HSV1<br>One-unit increase in IgG antibody level: NOT REPORTED<br>Highest antibody levels compared with the lowest levels (β=0.003, P>0.001).	Age, sex, education, income and chronic health conditions
Barnes, 2015	Cohort	CMV exposed: n=623 (73.4%)<br>Unexposed: n=226 (26.6%)<br><br>HSV1 exposed:n=370 (43.6%)<br>Unexposed: n=479 (56.4%)<br>Follow-up: 5.0 years (measure not given)	Overall<br>93 (11.0%) patients developed AD	Cox proportional hazard models	CMV: RR 2.15 (95% CI 1.42-3.27)<br>HSV1: RR 0.84 (95% CI 0.62–1.16)	Age, sex, education duration, race, vascular risk factors, vascular diseases, and apolipoprotein ε4 level
Bu, 2015	Case control	AD cases: n=128<br>Controls: n=135	CMV IgG seropositivity; AD cases: n=114/128 (89.1%), Controls: n=102/135 (75.6%)<br>HSV1 IgG seropositivity; AD cases: n=109/128 (85.2%), Controls: n=106/135 (78.5%)	Unconditional logistic regression analysis	CMV IgG seropositivity: OR 2.33 (95% CI 1.14-4.77)<br>HSV1 IgG seropositivity: OR 1.37 (95% CI 0.69-2.74)	Age, gender, education, APOE genotype, hypercholesterolemia, hypertension, CHD, diabetes
Carbone, 2014	Case control (nested)	AD Cases: n=36<br>Controls: n=106	EBV DNA<br>AD cases: n=19/36 (52.8%)<br>Controls: n=33/106 (31.1%)<br><br>HHV-6 DNA<br>AD cases: 7/32 (21.9%)<br>Controls: 3/68 (4.4%)<br><br>Data not available for IgG measures	Chi-square test and odds ratios calculated.	DNA<br>EBV DNA:OR 1.46 (95% CI 1.01-2.11)<br>HHV-6 DNA:OR 1.22 (95% CI 1.01-1.48)<br><br>IgG<br>IgG CMV titers: increased in AD cases (p =0.014).<br>IgG levels specific for EBNA and VCA increased in AD cases (EBNA: p =0.014; VCA: p = 0.05).<br>HHV-6 IgG seropositivity: no difference between cases and controls	None
Deng, 2016	Case control	Cases = 130 and controls = 147	HSV1: VCIND: n=93 (71.5%), Controls: n=98 (66.7%)<br>HSV-2: VCIND: n=31 (23.8%), Controls: n=10 (6.8%)<br>CMV: VCIND: n=100 (76.9%), Controls: n=89 (60.5%)	Chi-squared test	HSV1: OR 1.26 (95% CI 0.75-2.10)<br>HSV2: OR 4.29 (95% CI 2.01-9.16)<br>CMV: OR 2.17 (95% CI 1.28-3.67)<br>[calculated by review authors]	None
Hemling, 2003	Case control	AD cases: n=34<br>Controls: n=40	HSV1: AD cases: n=1/34 (2.9%), Controls: n=10/40 (25.0%)<br>HHV-6: AD cases: n= 30/34 (88.2%), Controls: n=35/40 (87.5%)<br>VZV: AD cases: n=9/34 (26.6%), Controls: n=11/40 (27.5%)	Multinomial logistic regression	HSV1: OR 0.1 (95% CI 0.02–0.5)<br>HHV-6: OR 1.9 (95% CI 0.4–10.6)<br>VZV: OR 0.8 (95% CI 0.2–2.8)	Age and sex
Itzhaki, 2005	Case control	VaD cases: n=18<br>Controls: n=29	CMV: VaD cases: n=15/18 (83%), Controls: n=10/29 (34%)<br>No data for HHV6 and HSV1: The proportions of the patients harbouring the other viruses in brain did not differ significantly from those of normals.	None	CMV: OR 9.5 (95% CI 2.21-40.79)<br>[calculated by review authors]	None
Kittur, 1992	Case control	AD Cases: n=5<br>Controls: n=5	No herpesvirus DNA detected in brain or blood samples.	None	OR 1.00 (95% CI 0.02-60.0) for all exposures<br>*[calculated by review authors]*	Matching factors only (age)
Letenneur, 2008	Cohort	IgG positive: n=424/512 (82.8%)<br>IgM positive: n=43/512 (8.4%)<br>Follow-up: mean 8.2 yr (SD=4.4)	IgG: Exposed: n=69/424 (16.3%), Unexposed: n=8/88 (9.1%)<br>IgM: Exposed: n=13/43 (30.2%), Unexposed: n=64/469 (13.7%)	Cox proportional hazards	HSV1/2 IgG: HR 1.67 (95% CI 0.75-3.73)<br>HSV1/2 IgM: HR 2.55 (95% CI 1.38-4.72)	Age at inclusion, gender, educational level, baseline MMSE and APOE-e4 allele
Lin, 1996	Case control	HSV1: AD cases: n=36, Controls: n=36<br>VZV: AD cases: n=17, Controls: n=12	HSV1: AD cases: n=28/36 (78%), Controls: n=23/36 (64%)<br>VZV: AD cases: n=0/17 (0%), Controls: n=0/12 (0%)	None	HSV1: OR 1.98 (95% CI 0.70-5.59)<br>VZV: OR 0.71 (95% CI 0.01-38.48)<br>*[calculated by review authors]*	None
Lin, 2002a (J of Pathology)	Case control	HSV1:<br>AD cases: n=61, Controls: n=48<br>HSV2:<br>AD cases: n=53, Controls: n=39<br>CMV:<br>AD cases: n=45, Controls: n=29<br>HHV6:<br>AD cases: n=50, Controls: n=35	HSV1:<br>AD cases: n=45/61 (73.8%), Controls: n=30/48 (62.5%)<br>HSV2:<br>AD cases: n=7/53 (13.2%), Controls: n=8/35 (22.9%)<br>CMV:<br>AD cases: n=16/45 (35.6%), Controls: n=10/29 (34%)<br>HHV6:<br>AD cases: n=36/50 (72.0%), Controls: n=14/35 (40.0%)	Logistic regression	HSV1: OR 1.69 (95% CI 0.75-3.82)<br>HSV2: OR 0.51 (95% CI 0.17-1.57)<br>CMV: OR 1.05 (95% CI 0.39-2.79)<br>*[results above calculated by review authors]*<br>HHV6: OR 3.86 (95% CI 1.54-9.64)	Matching factors only (age)
Lin, 2002b	Case control	VaD cases: n=15<br>Controls: n=38	CMV<br>VaD cases: n=14/15 (93.3%), Controls: n=10/29 (34%)<br>HHV6<br>VaD cases: n=7/15 (47%), Controls: n=14/35 (40%)<br>HSV1<br>VaD cases: n=9/15 (60%), Controls: n=30/48 (63%)* [data from controls taken from Lin et al 1998]	Logistic regression	CMV: OR 26.6 (95% CI 3.04-232.6)<br>HHV6: OR 1.31 (95% CI 0.39-4.44)<br>HSV1: OR 0.90 (95% CI 0.27-2.95) [data from controls taken from Lin et al 1998]	Matched on age only
Lovheim, 2015b	Case control (nested)	AD cases: n=360<br>Controls: n=360	IgG positive<br>Cases: n=338/360 (93.9%), Controls: n=324/360 (90.0%)<br>IgM positive:<br>Cases: n=27/360 (7.5%), Controls: n=20/360 (5.6%)	Conditional logistic regression	HSV1/2 IgG: OR 1.64 (95% CI 0.96-2.78)<br>HSV1/2 IgM: OR 1.37 (95% CI 0.76-2.47)	Matching factors only

etable 2: Study results

Author, yr	Design	Population size (N), follow-up time (years)	Subjects with outcome [or exposure for case-control studies] (N, %)	Statistical analysis method used	Main reported results	Adjusted for
Lovheim, 2015c	Cohort	IgG: Positive: n=3026 (88.2%), Negative: n=406 (11.8%)<br>IgM: Positive: n=100 (2.9%), Negative: n=3332 (97.1%)<br>Follow-up: mean 11.3 years (SD 5.7)	IgG: Exposed: n=231/3026 (7.6%),Unexposed: n=14/406 (3.4%)<br>IgM:Exposed: n=15/100 (15.0%), Unexposed: n=230/3332 (6.9%)	Cox proportional hazard regression	HSV1/2 IgG: HR 0.99 (95% CI 0.57-1.72)<br>HSV 1/2 IgM: HR 1.69 (95% CI 1.00-2.86)	Age and sex
Lycke, 1974	Case control	Dementia cases: n=105<br>Controls: n=100	HSV1/2: Dementia cases: n=94/105 (90%), Controls: n=67/100 (67%)<br>CMV: Dementia cases: n=90/105 (86%), Controls: n=50/100 (50%)<br>VZV: Dementia cases: n=29/105 (28%), Controls: n=37/100 (37%)	Logistic regression	HSV1/2: OR 4.21 (95% CI 1.98-8.92)<br>CMV: OR 6.00 (95% CI 3.06-11.76)<br>VZV: OR 0.65 (95% CI 0.36-1.17)<br>*[calculated by review authors]*	None
Mancuso, 2014b	Case control	AD cases: n=83<br>Controls: n=51	HSV1: seropositive; IgG>75th percentile [AI >10.1]<br>AD cases: n=81/83 (97.6%); n=26/83 (31%).<br>Controls: n=50/51 (98.0%); n=7/51 (14%)<br>CMV: seropositive; IgG>75th percentile [AU/ml>153.9]<br>AD cases: n=77/83 (93.0%); n=22/83 (26.2%)<br>Controls: n=50/51 (98.0%); n=11/51 (22.0%)	Chi-square	HSV1 seropositivity: OR 0.81 (95% CI 0.07-9.17)<br>CMV seropositivity: OR 0.26 (95% CI 0.03-2.20)<br>HSV1 high titre: OR 2.87 (95% CI 1.14-7.21)<br>CMV high titre: OR 1.31 (95% CI 0.57-3.00)<br>[calculated by review authors]	Matching factors only (age and gender)
Mann, 1981	Case control	AD cases: n=22<br>Controls: n=19	AD cases: n=1/22 (4.5%)<br>Controls: n=0/19 (0%)	None	HSV 1/2 OR 2.72 (95% CI 0.10-70.80)<br>[calculated by review authors]	None
Marques, 2001	Case control	AD cases: n=15<br>Controls: n=15	AD cases: 1/15 (6.7%)<br>Controls: 1/15 (6.7%)	None performed	HSV 1/2 OR 1.00 (95% CI 0.06-17.6)<br>[calculated by review authors]	None
Ounanian, 1990	Case control	AD cases: n=19<br>Controls: n=21	**<br>HSV1; AD cases: n=16/19 (84.2%), Controls: n=19/21 (90.5%)<br>CMV; AD cases: n=14/19 (73.7%), Controls: n=19/21 (90.5%)<br>VZV; AD cases: n=16/19 (84.2%), Controls: n=18/21 (85.7%)<br>EBV (VCA); AD cases: n=19/19 (100%), Controls: n=21/21 (100%)<br>EBV (EA); AD cases: n=8/19 (42.1%), Controls: n=15/21 (71.0%)<br>EBV (EBNA); AD cases: n=18/19 (94.7%), Controls: n=20/21 (95.2%)	Chi-squared test	HSV1; OR 0.56 (95% CI 0.08-3.79)<br>CMV; OR 0.29 (95% CI 0.05-1.75)<br>VZV; OR 0.89 (95% CI 0.16-5.05)<br>EBV (VCA); OR 0.9 (95% CI 0.02-47.9)<br>EBV (EA); OR 0.29 (95% CI 0.08-1.08)<br>EBV (EBNA); OR 0.9 (95% CI 0.05-15.47)<br>*[calculated by review authors]*	Matching factors only (age)
Renvoize, 1987	Case control	AD cases: n=33<br>Controls: n=28	Not available	t-test	CMV: 16.7 v. 19.5, t=0.797, 0.5>P>0.1;<br>HSV: 25.4 v. 20.5, t= 1.108, 0.5>P>0.1	None
Strandberg, 2003	Cohort	Total: N=383<br>0-1: n=48 (12.5%)<br>2: n=229 (59.8%)<br>3: n=106 (27.7%)<br>Follow-up: 1 year	Herpesvirus and MMSE decline not cross-tabulated.<br>MMSE score decreased in 150 patients (43.1%) during 1 year follow-up.	Logistic regression	Decrease in MMSE score during 1 year:<br>0-1: 1.00<br>2: OR 1.8 (95% CI 0.9-3.6)<br>3: OR 2.3 (95% CI 1.1-5.0)	MMSE score at baseline.
Taylor, 1986	Case control	AD cases: n=8<br>Controls: n=31	HSV1: AD cases: 0/8 (0%), Controls: 1/31 (6.5%)<br>CMV: AD cases: 0/8 (0%), Controls: 1/31 (6.5%)	None	HSV1 OR: 1.20 (95% CI 0.04-32.10)<br>CMV OR: 1.20 (95% CI 0.04-32.10)<br>[calculated by review authors]	None
Westman, 2017	Case control	AD cases: n=50<br>Controls: n=52	HHV6 PCR<br>AD cases: n=4/50 (8%)<br>Controls: n=4/52 (7.7%)	Mann–Whitney test	HHV-6 DNA: OR 1.04 (95% CI 0.25-4.42)<br>*HHV-6 IgG reactivity: Lower in AD patients (p=0.02)*<br>*CMV IgG reactivity: Non-significant (p=0.28)*<br>*HSV1 IgG reactivity: Non-significant*<br>*VZV IgG reactivity: Non-significant*	Matching factors only (age)
Wozniak, 2005	Case control	AD cases: n=27<br>Controls: n=13	HSV1 IgG specific AI≥1.5<br>AD cases: n=14/27 (52%), Controls: n=9/13 (69%)<br>(note: all subjects were seropositive for HSV1)<br>HHV8; AD cases: n=4/18 (22%), Controls: n=0/9 (0%)	Chi-squared test	HSV1: OR 0.48 (95% CI 0.12-1.94)<br>HHV6: OR 5.90 (95% CI 0.28-122.55)<br>[calculated by review authors]	Matching factors only (age)

Abbreviations: RCT=randomised controlled trial, SCCS=self-controlled case series, RR=risk (or rate) ratio, CI=confidence interval, AI=antibody index, KS=Kaposi's sarcoma, aMCI=amnestic mild cognitive impairment, ELISA=, NINCDS-ADRDA=National Institute of Neurological and Communicative Diseases and Stroke/AD and
*Or Odds Ratio (OR) if specified. Clinical Dementia Rating (CDR)
**Only percentages were given; the number seropositive was calculated and percentages amended where necessary.

Notes
Depending on amount of information, data on the secondary outcomes may be better in an appendices
Yellow highlight denotes that calculation of effect estimatesa has been checked and was correct. We used an online OR calculator and ensured that 0.5 as added to each cell of the two by two table if one of the cells was 0.
Red highlight denotes that calculation of effect estimates has been checked and was not correct: the calculation had not included +0.5 to every cell. It has been updated here and also in the metan spreadsheet.

etable 3 Risk of bias summary showing judgements about each risk of bias domain

Observational studies

First author, publication yr	Confounding	Selection of participants	Misclassification of variables	Bias due to missing data	Reverse Causation
**HSV-1 infection**					
Beffert, 1998	High risk	High risk	Low risk	Low risk	High risk
Bertrand, 1993	High risk	High risk	Low risk	Low risk	High risk
Cheon, 2015	High risk	High risk	Low risk	Low risk	High risk
Costa, 2017	Moderate risk	High risk	Low risk	Unclear risk	High risk
Deatly, 1990	High risk	High risk	Low risk	Low risk	High risk
Itabashi, 1997	High risk	High risk	Low risk	Low risk	High risk
Jamieson, 1991	High risk	High risk	Low risk	Low risk	High risk
Jamieson, 1992a	High risk	High risk	Low risk	Low risk	High risk
Jamieson, 1992b	High risk	High risk	Low risk	Low risk	High risk
Kobayashi, 2013	High risk	High risk	Low risk	Low risk	High risk
Lin, 1994	High risk	High risk	Low risk	Low risk	High risk
Lin, 1998	High risk	High risk	Low risk	Low risk	High risk
Mancuso, 2014a	High risk	Moderate risk	Low risk	Low risk	High risk
Mancuso, 2016	High risk	High risk	Low risk	Low risk	High risk
Mori, 2004	High risk	High risk	Low risk	Low risk	High risk
Roberts, 1986	High risk	High risk	Low risk	Low risk	High risk
Wozniak, 2009	High risk	High risk	Low risk	Low risk	High risk
**Varicella zoster virus reactivation (herpes zoster)**					
Tsai, 2017	Low risk	Low risk	Moderate risk	Low risk	Low risk
**HSV-1 reactivation**					
Helmer, 2015	Moderate risk	Moderate risk	Low risk	Low risk	Low risk
**Epstein-Barr virus infection**					
Shim, 2017	Moderate risk	High risk	Low risk	Low risk	Low risk
**Cytomegalovirus infection**					
Lovheim, 2015a	Moderate risk	Low risk	Low risk	Low risk	Low risk
Kawasaki 2016	Moderate risk	Moderate risk	Low risk	Unclear risk	Low risk
Renvoize, 1984	High risk	High risk	Low risk	Low risk	High risk
Westman, 2013	High risk	Moderate risk	Low risk	Low risk	High risk
**Human herpesvirus 8 infection**					
Dore, 1998	High risk	Unclear risk	High risk	Low risk	Low risk
Dupin, 2000	High risk	Unclear risk	High risk	Low risk	Low risk
Mary-Krause, 1999	Low risk	Low risk	High risk	Low risk	Low risk
Polk, 2002	Moderate risk	Low risk	Low risk	Low risk	Low risk
Renwick, 2001	High risk	Unclear risk	Low risk	Unclear risk	Low risk
Rezza, 1999	Moderate risk	Moderate risk	Low risk	Unclear risk	Low risk
**Multiple herpesviruses**					
Agostini, 2016a	High risk	Unclear risk	Low risk	Unclear risk	Low risk
Agostini, 2016b	High risk	High risk	Low risk	Low risk	High risk
Aiello 2006	Moderate risk	Moderate risk	Low risk	Low risk	Low risk
Barnes, 2015	Moderate risk	Moderate risk	Low risk	Low risk	Low risk
Bu, 2015	Moderate risk	Moderate risk	Low risk	Low risk	High risk
Carbone, 2014	High risk	High risk	Low risk	High risk	High risk
Deng 2016	High risk	High risk	Low risk	Low risk	High risk
Hemling, 2003	Moderate risk	High risk	Low risk	Low risk	High risk
Itzhaki, 2005	High risk	High risk	Low risk	Low risk	High risk
Kittur, 1992	High risk	Moderate risk	Low risk	Low risk	High risk
Letenneur, 2008	Moderate risk	High risk	Low risk	Low risk	Low risk
Lin, 1996	High risk	High risk	Low risk	Low risk	High risk
Lin, 2002a	High risk	High risk	High risk	High risk	High risk
Lin, 2002b	High risk	High risk	High risk	Low risk	High risk
Lovheim, 2015b	Moderate risk	Low risk	Low risk	Low risk	Low risk
Lovheim, 2015c	Moderate risk	Low risk	Low risk	Low risk	Low risk
Lycke, 1974	High risk	High risk	High risk	Low risk	High risk
Mancuso, 2014b	High risk	Moderate risk	Low risk	Low risk	High risk
Mann, 1981	High risk	High risk	Low risk	Low risk	High risk
Marques, 2001	High risk	High risk	High risk	Low risk	High risk
Ounanian, 1990	High risk	High risk	Low risk	Low risk	High risk
Renvoize, 1987	High risk	High risk	Low risk	Low risk	High risk
Strandberg, 2003	Moderate risk	Low risk	Low risk	Low risk	Low risk
Taylor, 1986	High risk	High risk	Low risk	Low risk	High risk
Westman, 2017	High risk	Moderate risk	Low risk	Low risk	High risk
Wozniak, 2005	High risk	Moderate risk	Low risk	Low risk	High risk

RCTS

First author, publication yr	Selection bias	Performance bias	Detection bias	Attrition bias	Reporting bias
**HSV-1 infection**					
Gnann, 2000	Low risk	Low risk	Unclear risk	Low risk	Low risk

Key
- High risk
- Moderate risk
- Low risk
- Unclear risk

etable 4 Assessment of quality of evidence for outcomes

Quality assessment							Quality
**№ of studies**	**Study design**	**Risk of bias**	**Inconsistency**	**Indirectness**	**Imprecision**	**Other considerations**	
CMV: past infection							
9	observational studies	very serious [a]	very serious [b]	not serious	serious [c]	none	⨁◯◯◯ VERY LOW
CMV: recent infection/reactivation							
4	observational studies	very serious [a]	very serious [b]	not serious	very serious [d]	none	⨁◯◯◯ VERY LOW
EBV: past infection							
1	observational studies	very serious [a]	serious [e]	not serious	very serious [d]	none	⨁◯◯◯ VERY LOW
EBV: recent infection/reactivation							
2	observational studies	very serious [a]	serious [e]	not serious	very serious [d]	none	⨁◯◯◯ VERY LOW
HSV-1: past infection							
11	observational studies	very serious [a]	serious [f]	not serious	very serious [d]	none	⨁◯◯◯ VERY LOW
HSV-1: recent infection/reactivation							
19	observational studies	very serious [a]	serious [f]	not serious	very serious [d]	none	⨁◯◯◯ VERY LOW
HSV-2: recent infection/reactivation							
2	observational studies	very serious [a]	serious [f]	not serious	very serious [d]	none	⨁◯◯◯ VERY LOW
HSV-1/2: past infection							
4	observational studies	serious [g]	very serious [b]	not serious	serious [c]	none	⨁◯◯◯ VERY LOW
HSV-1/2: recent infection/ reactivation							
6	observational studies	serious [g]	very serious [b]	not serious	very serious [d]	none	⨁◯◯◯ VERY LOW
VZV: past infection							
2	observational studies	very serious [a]	not serious	not serious	serious [c]	none	⨁◯◯◯ VERY LOW
VZV: recent infection/ reactivation							
2	observational studies	very serious [a]	not serious	not serious	very serious [d]	none	⨁◯◯◯ VERY LOW
HHV6: past infection							
3	observational studies	very serious [a]	very serious [b]	not serious	very serious [d]	none	⨁◯◯◯ VERY LOW
HHV6: recent infection/reactivation							
5	observational studies	very serious [a]	serious [h]	not serious	serious [c]	strong association	⨁◯◯◯ VERY LOW
HHV8: past infection							
6	observational studies	serious [g]	serious [i]	not serious	serious [c]	none	⨁◯◯◯ VERY LOW
VZV reactivation (herpes zoster)							
1	observational studies	not serious	serious [j]	not serious	not serious	very strong association	⨁⨁⨁◯ MODERATE

a. Study/studies suffered from two or more domains at high risk of bias.

b. Wide variance of point estimates across studies, minimal overlap of confidence intervals, where meta-analyses were possible I^2 statistic indicates statistical heterogeneity.

c. Wide confidence interval(s)

d. Very wide confidence interval(s)

e. Some variance of point estimates across studies, confidence intervals overlap, statistical evidence of heterogeneity not investigated

f. Some point estimates across studies in different directions, some overlap of confidence intervals, where meta-anlayses were possible I^2 statistic indicates little heterogeneity

g. <50% studies have two or more domains at high risk of bias and contribute <50% weight to the meta-analysis

h. Variance in point estimates across studies (but in same direction), some overlap of confidence intervals, where meta-anlayses were possible I^2 statistic indicates little heterogeneity

i. Variance in point estimates across studies (but in same direction), some overlap of confidence intervals, where meta-anlayses were possible I^2 statistic indicates considerable heterogenei

j. Only one study therefore difficult to assess

PART C: additional figures

efigure 1: Effect of herpesvirus infections on MCI risk

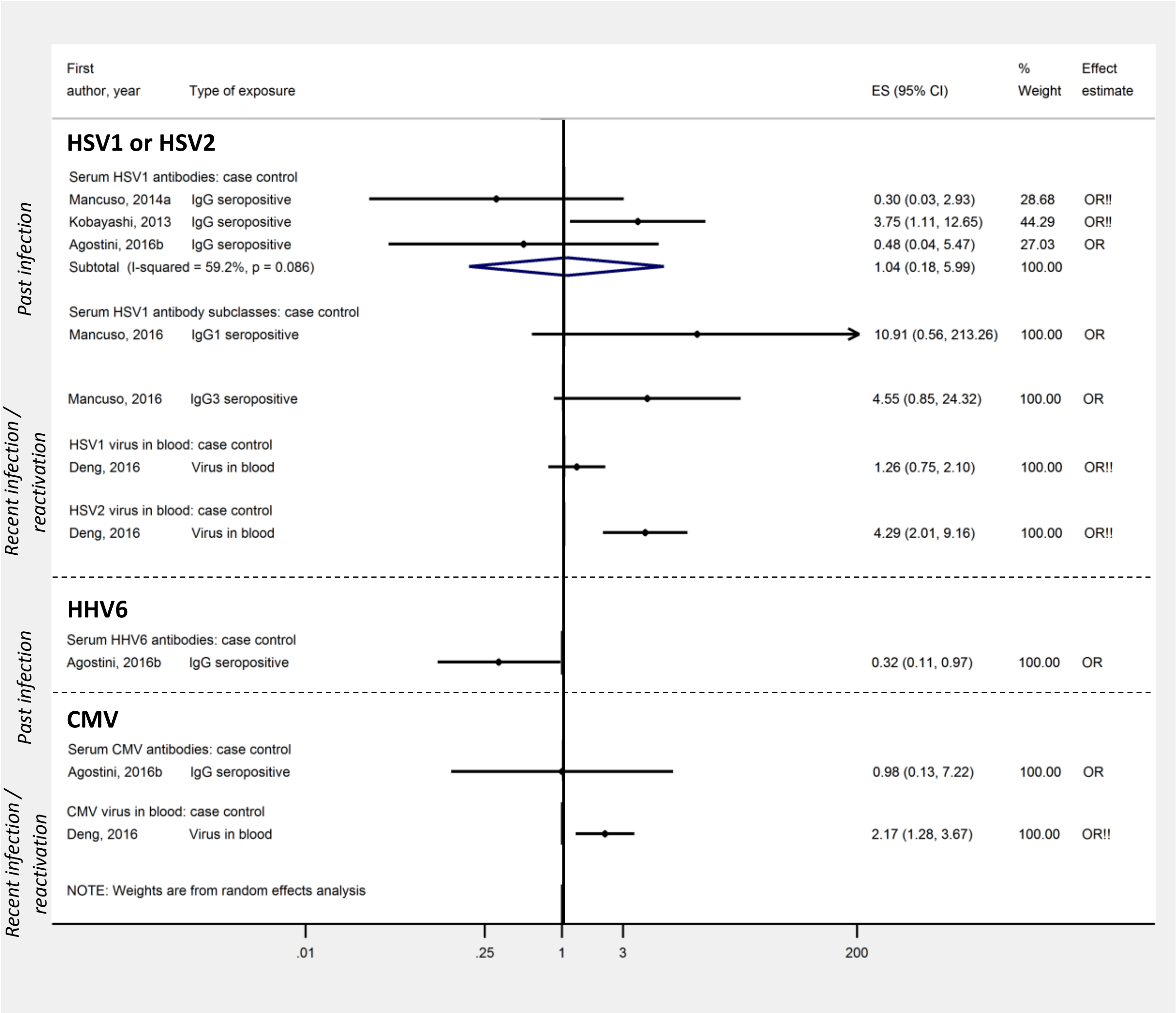

‼No age adjustment/matching for age

efigure 2: Effect of herpesviruses type 6 and 8 on dementia risk

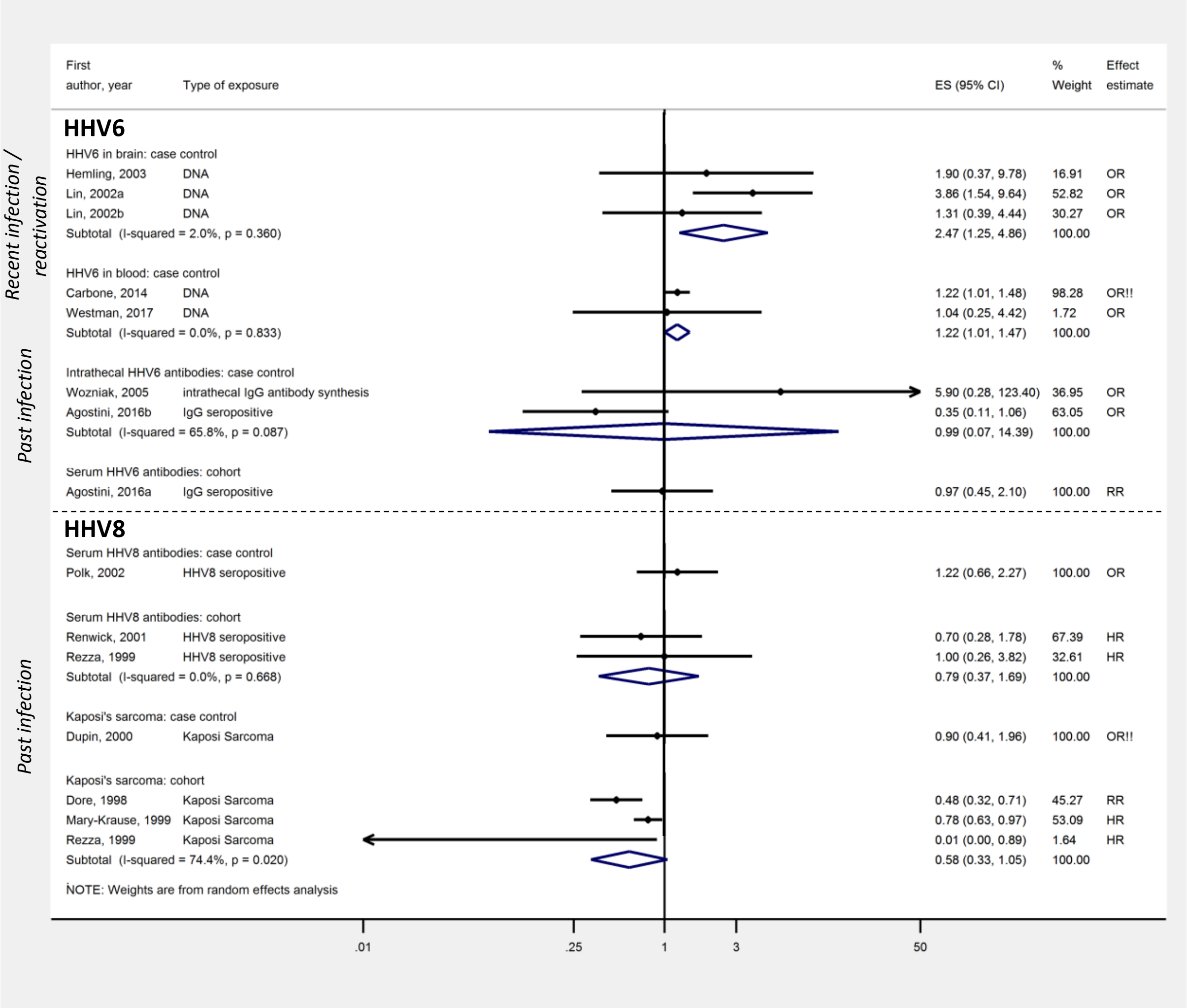

‼No age adjustment/matching for age. All dementia in studies assessing HHV-8 were AIDs-related dementia, except for one study (Polk, 2002) where the dementia type was unspecified.

efigure 3: Assessment of publication bias for HSV1 DNA in the brain (from case-control studies) as a risk factor for dementia

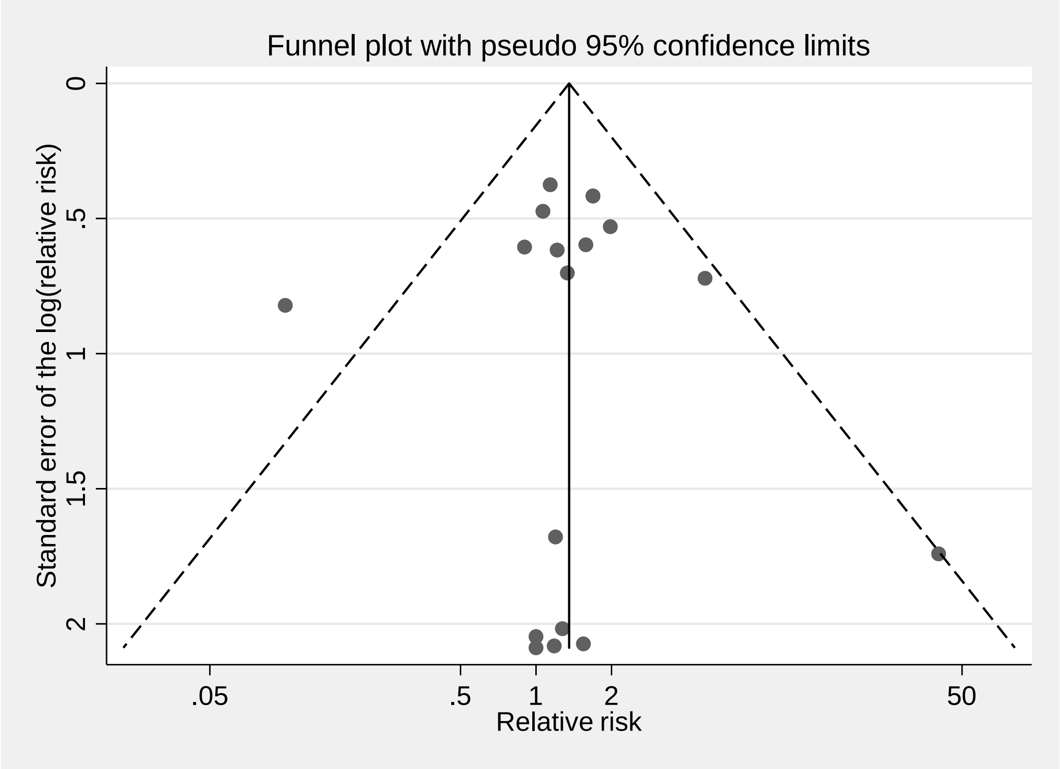

Funnel plot of the log odds ratio plotted against the standard error of the log odds ratio for 17 studies reporting the effect of HSV1 DNA in the brain on dementia risk (dotted line represents pseudo 95% confidence limits).

Note: Using Begg's test for small-study effects: P=0·88

Subgroup analyses

efigure A1: Effect of herpesvirus infections on dementia risk, by age and sex

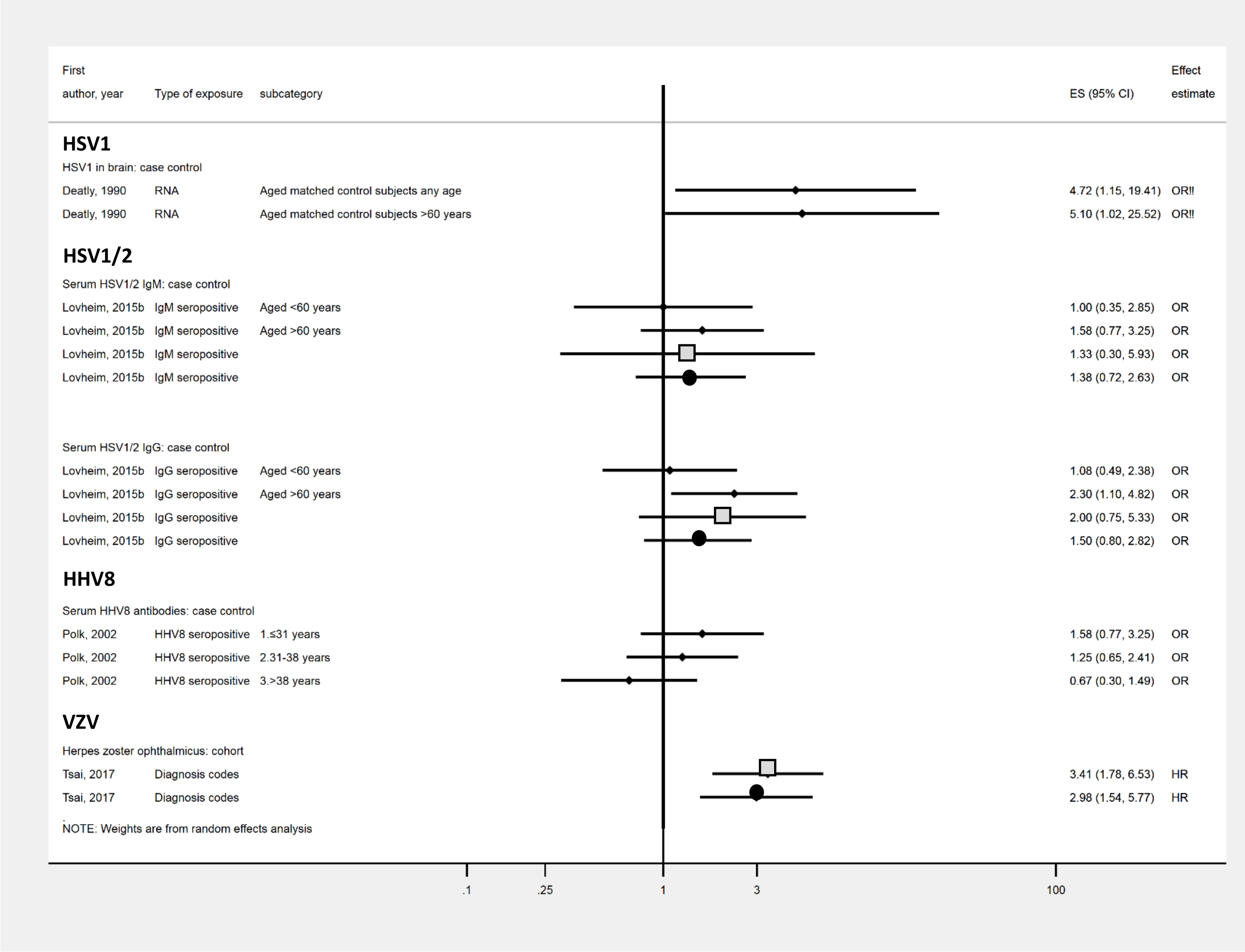

efigure A2: Effect of herpesvirus infections on dementia risk, by APOE type

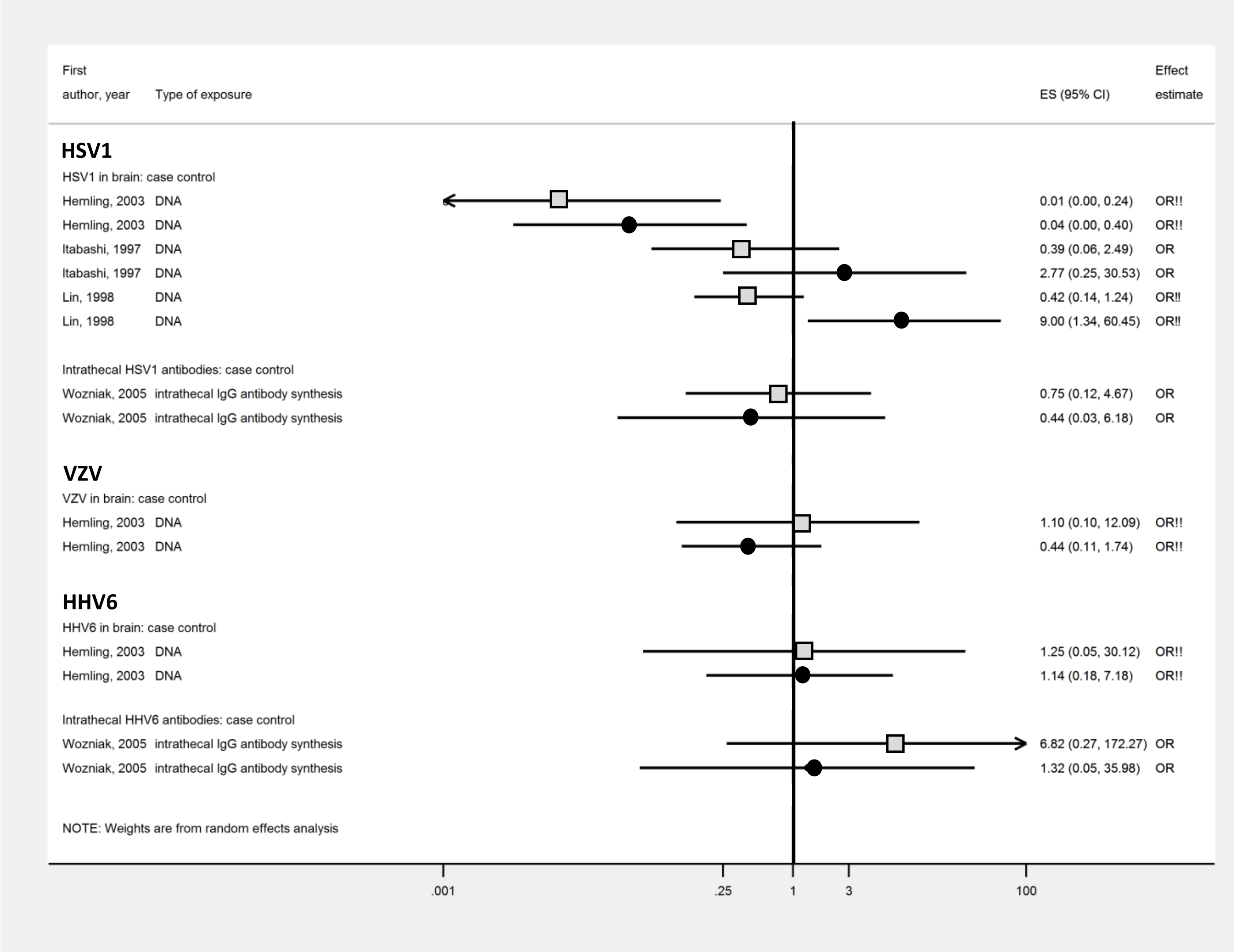

‼No age adjustment/matching for age

□ APOE-ε4 negative

● APOE-ε4 positive

efigure A3: Effect of herpesvirus infections on dementia risk, by outcome type

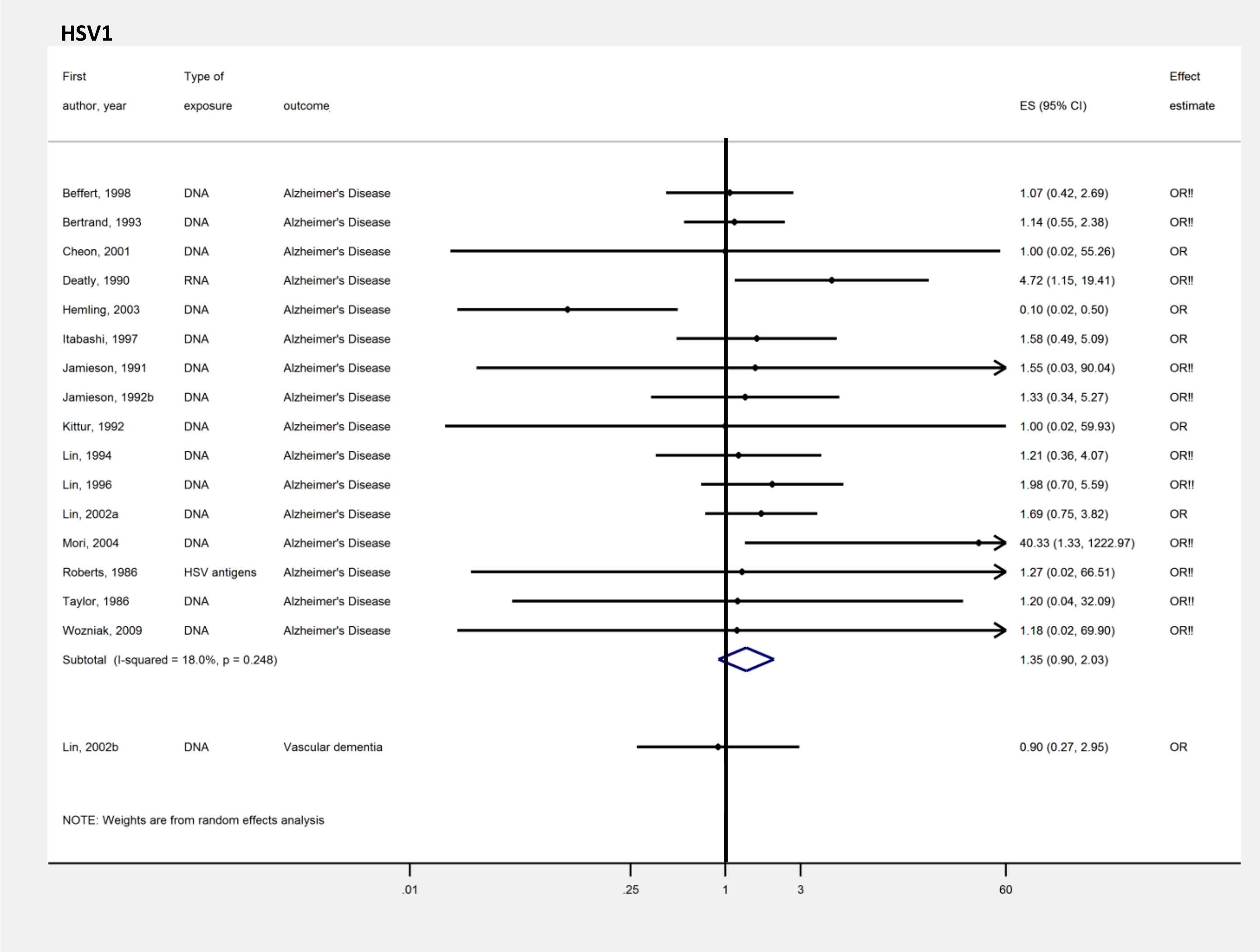

HSV1/2

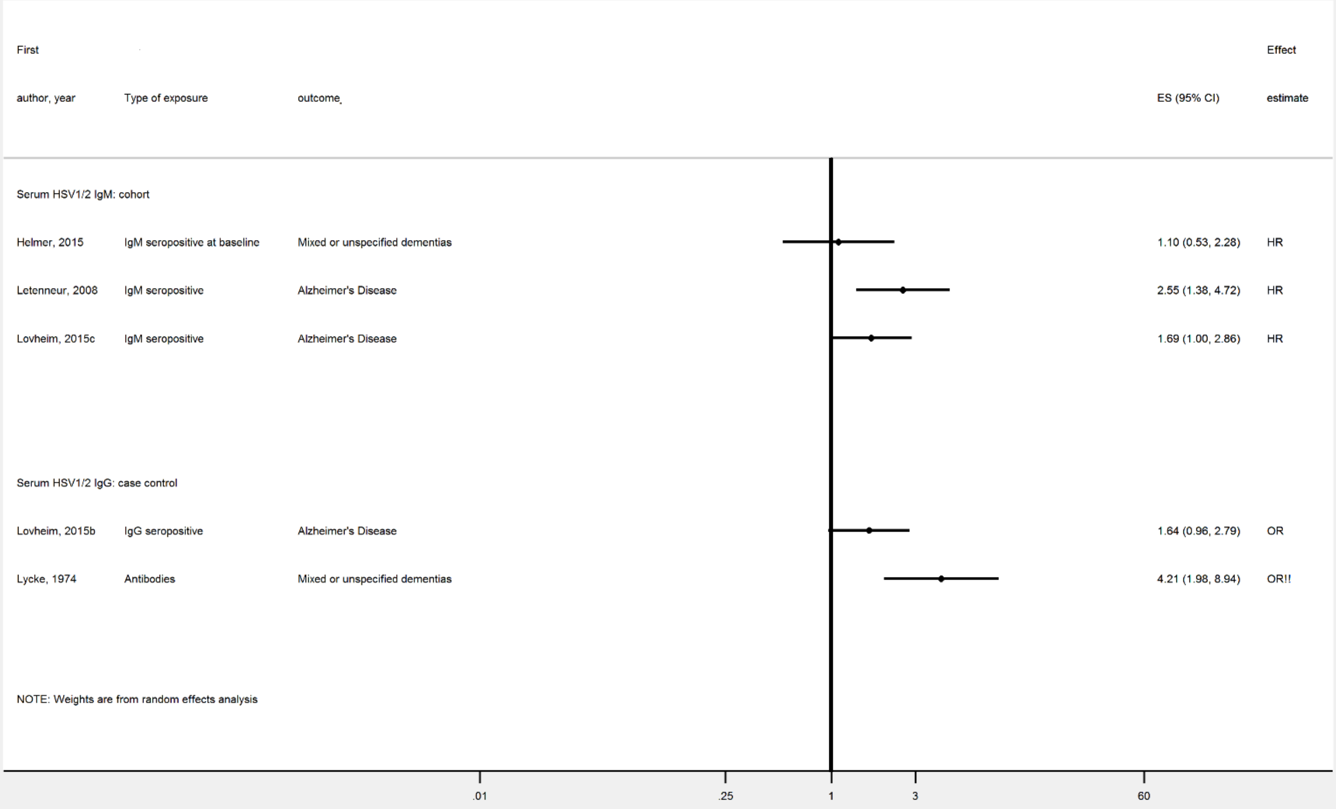
First
author, year
Type of exposure
outcome
ES (95% CI)
Effect
estimate
Serum HSV1/2 IgM: cohort
Helmer, 2015
IgM seropositive at baseline
Mixed or unspecified dementias
1.10 (0.53, 2.28)
HR
Letenneur, 2008
IgM seropositive
Alzheimer's Disease
2.55 (1.38, 4.72)
HR
Lovheim, 2015c
IgM seropositive
Alzheimer's Disease
1.69 (1.00, 2.86)
HR
Serum HSV1/2 IgG: case control
Lovheim, 2015b
IgG seropositive
Alzheimer's Disease
1.64 (0.96, 2.79)
OR
Lycke, 1974
Antibodies
Mixed or unspecified dementias
4.21 (1.98, 8.94)
OR!!
NOTE: Weights are from random effects analysis
.01
.25
1
3
60

VZV

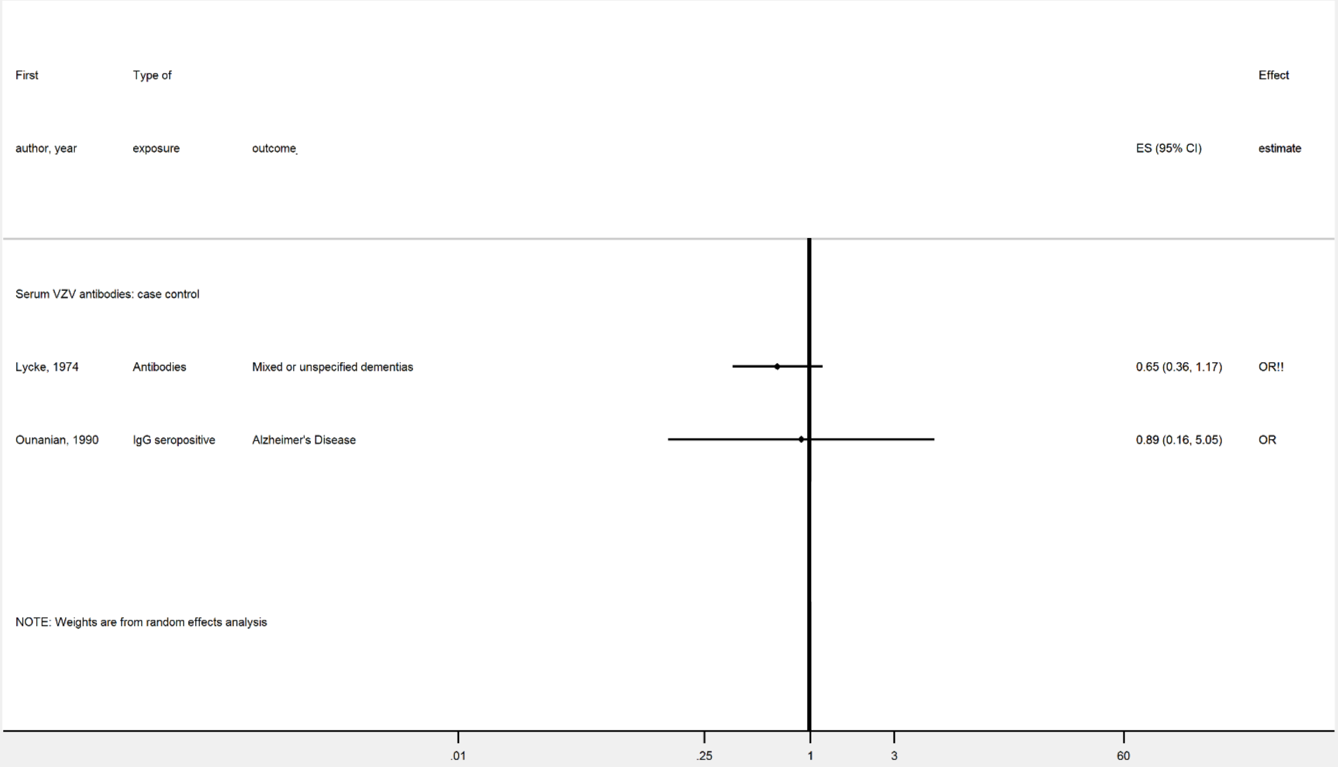
First
author, year
Type of
exposure
outcome
ES (95% CI)
Effect
estimate
Serum VZV antibodies: case control
Lycke, 1974
Antibodies
Mixed or unspecified dementias
0.65 (0.36, 1.17)
OR!!
Ounanian, 1990
IgG seropositive
Alzheimer's Disease
0.89 (0.16, 5.05)
OR
NOTE: Weights are from random effects analysis
.01
.25
1
3
60

CMV

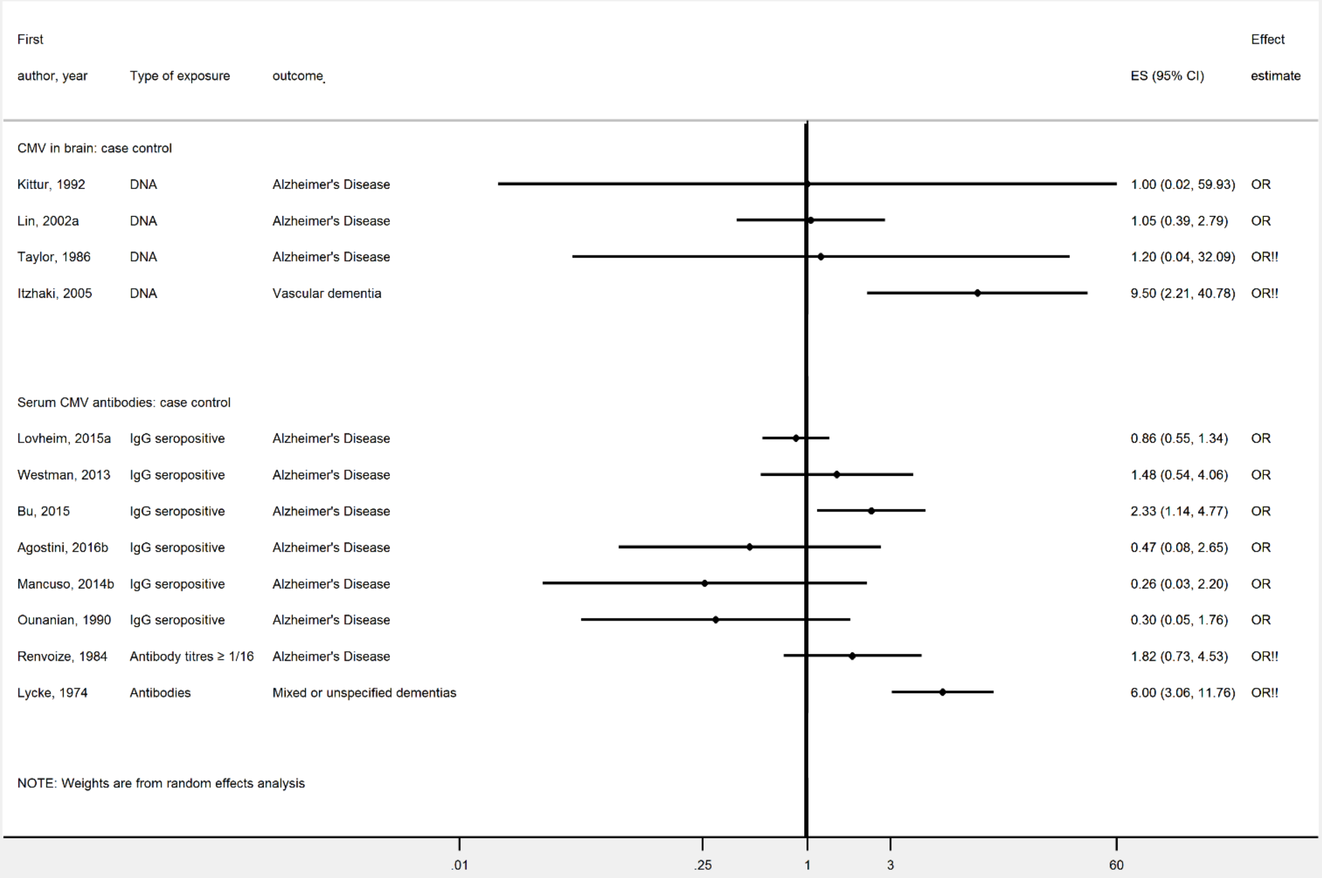
First
author, year
Type of exposure
outcome
ES (95% CI)
Effect
estimate
CMV in brain: case control
Kittur, 1992 DNA Alzheimer's Disease 1.00 (0.02, 59.93) OR
Lin, 2002a DNA Alzheimer's Disease 1.05 (0.39, 2.79) OR
Taylor, 1986 DNA Alzheimer's Disease 1.20 (0.04, 32.09) OR!!
Itzhaki, 2005 DNA Vascular dementia 9.50 (2.21, 40.78) OR!!
Serum CMV antibodies: case control
Lovheim, 2015a IgG seropositive Alzheimer's Disease 0.86 (0.55, 1.34) OR
Westman, 2013 IgG seropositive Alzheimer's Disease 1.48 (0.54, 4.06) OR
Bu, 2015 IgG seropositive Alzheimer's Disease 2.33 (1.14, 4.77) OR
Agostini, 2016b IgG seropositive Alzheimer's Disease 0.47 (0.08, 2.65) OR
Mancuso, 2014b IgG seropositive Alzheimer's Disease 0.26 (0.03, 2.20) OR
Ounanian, 1990 IgG seropositive Alzheimer's Disease 0.30 (0.05, 1.76) OR
Renvoize, 1984 Antibody titres ≥ 1/16 Alzheimer's Disease 1.82 (0.73, 4.53) OR!!
Lycke, 1974 Antibodies Mixed or unspecified dementias 6.00 (3.06, 11.76) OR!!
NOTE: Weights are from random effects analysis
.01
.25
1
3
60

HHV6

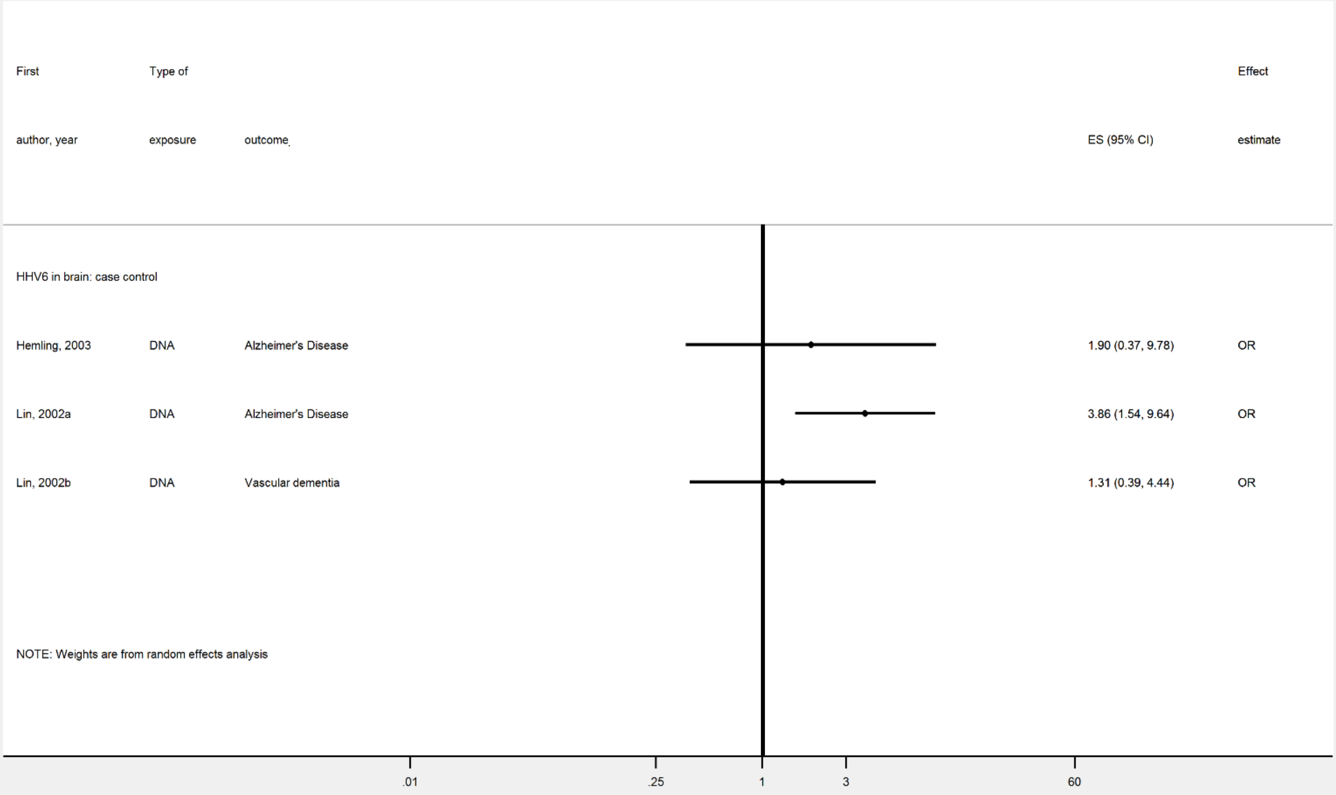
First
author, year
Type of
exposure
outcome
ES (95% CI)
Effect
estimate
HHV6 in brain: case control
Hemling, 2003 DNA Alzheimer's Disease 1.90 (0.37, 9.78) OR
Lin, 2002a DNA Alzheimer's Disease 3.86 (1.54, 9.64) OR
Lin, 2002b DNA Vascular dementia 1.31 (0.39, 4.44) OR
NOTE: Weights are from random effects analysis
.01
.25
1
3
60

PART D: additional tables

etable A1: Exploring statistical heterogeneity by removing studies at high risk of bias

	No. of studies	Summary RR (95% CI)	I^2
**OUTCOME: DEMENTIA**			
**Serum HSV1/2 IgM: cohort**			
Results from primary analysis including all studies	3	1.73 (1.12-2.86)	33.6%
No studies had more than one domain with a high risk of bias			
**Serum HSV1/2 IgG: cohort**			
Results from primary analysis including all studies	2	2.53 (1.01-6.34)	75.1%
Neither study had more than one domain with a high risk of bias			
**CMV in brain: case-control**			
Results from primary analysis including all studies	4	2.25 (0.55-9.25)	52.1%
All studies had more than one domain with a high risk of bias			
**Serum CMV antibodies: case-control**			
Results from primary analysis including all studies	8	1.30 (0.65-2.63)	77.7%
Results after removing studies with more than one domain at high risk of bias	2	1.36 (0.51-3.60)	81.4%
**Intrathecal HHV6 antibodies: case control**			
Results from primary analysis including all studies	2	0.99 (0.07-14.39)	65.8%
All studies had more than one domain with a high risk of bias			
**Kaposi's sarcoma: cohort**			
Results from primary analysis including all studies	3	0.58 (0.33-1.05)	74.4%
Results after removing studies with more than one domain at high risk of bias	2	0.16 (0.00-9.76)	72.2%
**OUTCOME: MCI**			
**Serum HSV1 antibodies: case control**			
Results from primary analysis including all studies	3	1.04 (0.18-5.99)	59.2%
All studies had more than one domain with a high risk of bias			

Note: If a meta-analysis combined only two studies we did not explore heterogeneity

etable A2 Risk of bias summary with judgement and justification for each risk of bias domain

	Confounding	Selection of participants		Misclassification of variables										Bias due to missing data		Reverse causation	Generalisability
	Age and other confounders	Participation bias	Selection of controls	Exposure				Outcome				Covariates		Differential loss to follow up	Exclusion of individuals with missing data		
				Differential			Non-differential	Differential			Non-differential	Differential	Non-differential				
				Recall bias	Observer bias	Ascertainment bias		Recall bias	Observer bias	Ascertainment bias							
Criteria for risk of bias assessment	**Low:** Adjusted for age with several categories or continuous if appropriate. Measued at baseline and time-updated if necessary. **Moderate:** Age-adjusted but with less detail (eg binary). **High:** Not adjusted for age.	**Low:** (1) automated participation (e.g. medical record review), or (2) random sample. **Moderate:** Non-random sample, with evidence that those included and not included in the study have different characteristics in terms of age and gender. **High:** Non-random sample, with no comparison of characteristics of those included/excluded.	**Low:** controls selected using random sampling (or other system unlikely to be biased by exposure status) from the population from which the cases arose. **Moderate:** Controls not selected from the population from which the cases arose OR controls not selected using random sampling. **High:** probability of selection as a control likely to be affected	**Low:** exposure status defined before occurance of stroke. **High:** exposure status defined by patient recall of exposure in context of recent stroke	**Low:** exposure status defined before occurance of stroke OR exposure status defined by observer blinded to outcome (stroke) status. **High:** exposure status defined by observer unblinded to outcome (stroke) status, without clear objective criteria to apply.	**Low:** exposure status defined before occurance of stroke. **High:** participants with stroke are more or less likely to be tested for exposures.	members of study assessed for exposure at baseline. Exposure defined using laboratry criteria. **High:** allocation of exposure status relies on exposure having been defined by diagnosed as patient recall part of routine of stroke in medical care: context of exposure may herpes virus therefore be exposure.	**Low:** stroke status defined after exposure status assigned. **High**: stroke status	**Low:** stroke status defined after exposure status assigned. **High:** stroke status defined by observer in context likely to be influenced by herpesvirus status.	**Low:** ascertainment of stroke unlikely to be influenced by herpesvirus status. **High**: ascertainment of stroke likely to be influenced by herpesvirus status.	**Low:** Active data collection of outcome, or outcome unlikely to be missed or presents validation results of >70% sensitivity and specificity. **High:** Unclear or unvalidated methd of diagnosis.	Is there differential misclassification of covariates by exposure or outcome?	Is there non-differential misclassification of covariates?	**Low:** (1) automated follow up (e.g. through record linkage), or (2) ≥80% follow up, or (3) 70-80% follow up with a comparison (min age, sex) showing similar characteristics between those included and those not included in the study. **Medium:** 70-80% follow up with a comparison (min age, sex) showing dissimilar characteristics between those included and those not included in the study. **High:** <70% follow-up that will likely impact conclusions, with no discussion/analysis to assess impact.	**Low**: None or very low percentage of missing data, or appropriate missing data technique used). Sensitivity analysis performed to assess potential impact of missingness. **Moderate**: Substantial missing data (15-25%) with no or inappropriate method used. **High:** Large amount of missing data (>25 %) with no discussion /attempt to assess impact, or inappropriate method used.	**Low:** exposure defined prior to ascertainment of stroke. **High**: exposure defined after the stroke defined as the study outcome.	
HSV-1 infection																	
Beffert, 1998	High: no confounders taken into account.	High: No information on how cohort was sampled.	High: no information on how controls were selected.	Low: exposure status defined through brain sample tests: doesn't rely on patient recall	Low: unclear if exposure status defined by observer blinded to outcome status.	Low: all cases and controls had brain samples tested.	Low: defined using laboratory test.	Low: AD diagnosis doesn't rely on patient recall	Low: clinician diagnosing AD unlikely to be aware of exposure status	Low: ascertainment of AD unlikely to be influenced by herpesvirus status.	Low: AD diagnosed neuropathologically	No covariates	No covariates	Low: case control study	Unclear: missing data not reported	High: as brain samples tested following AD diagnosis	Generalisable to older inidividuals
Bertrand, 1993	High: no confounders taken into account.	High: No information on how cohort was sampled.	High: no information on how controls were selected.	Low: exposure status defined through brain sample tests: doesn't rely on patient recall	Low: unclear if exposure status defined by observer blinded to outcome status.	Low: all cases and controls had brain samples tested.	Low: defined using laboratory test.	Low: AD diagnosis doesn't rely on patient recall	Low: clinician diagnosing AD unlikely to be aware of exposure status	Low: ascertainment of AD unlikely to be influenced by herpesvirus status.	Low: AD diagnosed neuropathologically	No covariates	No covariates	Low: case control study	Unclear: missing data not reported	High: as brain samples tested following AD diagnosis	Generalisable to older inidividuals
Cheon, 2015	High: matched on age, however no confounders taken into account.	High: No information on how cohort was sampled.	High: controls were selected using non-random sampling method (consecutive series), nor is it clear whether they come from the same population from which cases arose.	Low: exposure status defined through brain sample tests: doesn't rely on patient recall	Low: unclear if exposure status defined by observer blinded to outcome status.	Low: all cases and controls had brain samples tested.	Low: defined using laboratory test.	Low: AD diagnosis doesn't rely on patient recall	Low: clinician diagnosing AD unlikely to be aware of exposure status	Low: ascertainment of AD unlikely to be influenced by herpesvirus status.	Low: AD diagnosed by a psychiatrist.	No covariates relevant as we are preseting crude OR only	No covariates relevant as we are preseting crude OR only	Low: case control study	Unclear: missing data not reported	High: as brain samples tested following AD diagnosis	Generalisable to older inidividuals
Deatly, 1990	High: no confounders taken into account.	High: No information on how cohort was sampled.	High: no information on how controls were selected.	Low: exposure status defined through brain sample tests: doesn't rely on patient recall	Low: unclear if exposure status defined by observer blinded to outcome status.	Low: all cases and controls had brain samples tested.	Low: defined using laboratory test.	Low: AD diagnosis doesn't rely on patient recall	Low: clinician diagnosing AD unlikely to be aware of exposure status	Low: ascertainment of AD unlikely to be influenced by herpesvirus status.	Low: diagnosed using diagnostic criteria.	No covariates	No covariates	Low: case control study	Unclear: missing data not reported	High: as brain samples tested following AD diagnosis	Unclear
Itabashi, 1997	High: no confounders taken into account.	High: No information on how cohort was sampled.	High: no information on how controls were selected.	Low: exposure status defined through blood tests: doesn't rely on patient recall	Low: exposure status defined by observer blinded to outcome status.	Low: all cases and controls had blood samples tested.	Low: defined using laboratory test.	Low: AD diagnosis doesn't rely on patient recall	Low: clinician diagnosing AD unlikely to be aware of exposure status	Low: ascertainment of AD unlikely to be influenced by herpesvirus status.	Low: diagnosed using diagnostic criteria.	No covariates	No covariates	Low: case control study	Unclear: missing data not reported	High: as blood samples tested following AD diagnosis	Unclear
Jamieson, 1991	High: no confounders taken into account.	High: No information on how cohort was sampled.	High: no information on how controls were selected.	Low: exposure status defined through brain sample tests: doesn't rely on patient recall	Low: unclear if exposure status defined by observer blinded to outcome status.	Low: all cases and controls had brain samples tested.	Low: defined using laboratory test.	Low: AD diagnosis doesn't rely on patient recall	Low: clinician diagnosing AD unlikely to be aware of exposure status	Low: ascertainment of AD unlikely to be influenced by herpesvirus status.	Unclear: no information on how AD was defined.	No covariates	No covariates	Low: case control study	Unclear: missing data not reported	High: as brain samples tested following AD diagnosis	Unclear

Observational Studies																		
	**Confounding**	**Selection of participants**		**Misclassification of variables**										**Bias due to missing data**				
	Age and other confounders	Participation bias	Selection of controls	Exposure				Outcome				Covariates		Differential loss to follow up	Exclusion of individuals with missing data	**Reverse causation**	**Generalisability**
				Differential			Non-differential	Differential			Non-differential	Differential	Non-differential				
				Recall bias	Observer bias	Ascertainment bias		Recall bias	Observer bias	Ascertainment bias							
Jamieson, 1992a	High: no confounders taken into account.	High: No information on how cohort was sampled.	High: no information on how controls were selected.	Low: exposure status defined through blood tests: doesn't rely on patient recall	Low: unclear if exposure status defined by observer blinded to outcome status.	Low: all cases and controls had blood samples tested.	Low: defined using laboratory test.	Low: AD diagnosis doesn't rely on patient recall	Low: clinician diagnosing AD unlikely to be aware of exposure status	Low: ascertainment of AD unlikely to be influenced by herpesvirus status.	Low: diagnosed using diagnostic criteria.	No covariates	No covariates	Low: case control study	Unclear: missing data not reported	High: as blood samples tested following AD diagnosis	Unclear
Jamieson, 1992b	High: no confounders taken into account. Did compare to elderly controls in main analysis, however no matching or adjusting for age.	High: No information on how cohort was sampled.	Moderate: no clear if controls selected randomly, but did derive from same brain banks as cases	Low: exposure status defined through PCR: doesn't rely on patient recall	Low: unclear if exposure status defined by observer blinded to outcome status.	Low: all cases and controls had samples tested.	Low: defined using laboratory test.	Low: AD diagnosis doesn't rely on patient recall	Low: observer diagnosing AD unlikely to be aware of exposure status	Low: ascertainment of AD unlikely to be influenced by herpesvirus status.	Low: diagnosed using clinical and neuropathalogical criteria.	No covariates	No covariates	Low: case control study	Unclear: missing data not reported	High: as brain samples tested following AD diagnosis	Unclear
Kobayashi, 2013	High: no confounders taken into account (although the authors didn't find an association between HSV-1 antibody levels and age).	High: No information on how cohort was sampled.	High: no information on how or where the controls were selected. It is unclear if they came from the same population that the cases arose from.	Low: exposure status defined through serum sample tests: doesn't rely on patient recall	Low: unclear if exposure status defined by observer blinded to outcome status.	Low: all cases and controls had serum samples tested.	Low: defined using laboratory test.	Low: AD/aMCI diagnosis doesn't rely on patient recall	Low: observer diagnosing AD/aMCI unlikely to be aware of exposure status	Low: ascertainment of AD/aMCI unlikely to be influenced by herpesvirus status.	Low: diagnosed using clinical criteria.	No covariates	No covariates	Low: case control study	Unclear: missing data not reported	High: as blood samples tested following AD diagnosis	Unclear
Lin, 1994	High: no confounders taken into account.	High: No information on how cohort was sampled.	High: no information on how controls were selected.	Low: exposure status defined through brain sample tests: doesn't rely on patient recall	Low: unclear if exposure status defined by observer blinded to outcome status.	Low: all cases and controls had brain samples tested.	Low: defined using laboratory test.	Low: AD diagnosis doesn't rely on patient recall	Low: clinician diagnosing AD unlikely to be aware of exposure status	Low: ascertainment of AD unlikely to be influenced by herpesvirus status.	Unclear: no information on how AD was defined.	No covariates	No covariates	Low: case control study	Unclear: missing data not reported	High: as brain samples tested following AD diagnosis	Unclear
Lin, 1998	High: no confounders taken into account.	High: No information on how cohort was sampled.	High: no information on how controls were selected.	Low: exposure status defined through brain sample tests: doesn't rely on patient recall	Low: exposure status defined by observer blinded to outcome status.	Low: all cases and controls had brain samples tested.	Low: defined using laboratory test.	Low: AD diagnosis doesn't rely on patient recall	Low: clinician diagnosing AD unlikely to be aware of exposure status	Low: ascertainment of AD unlikely to be influenced by herpesvirus status.	Low: diagnosed using diagnostic criteria.	No covariates	No covariates	Low: case control study	Unclear: missing data not reported	High: as brain samples tested following AD diagnosis	Unclear
Mancuso, 2014a	High: no confounders taken into account.	Moderate: non-random sampling (consecutive recruitment); good description of exclusion criteria but no reporting of numbers excluded.	Moderate: controls not selected randomly, but did derive from same population as cases.	Low: exposure status defined through serum sample tests: doesn't rely on patient recall	Low: unclear if exposure status defined by observer blinded to outcome status.	Low: all cases and controls had serum samples tested.	Low: defined using laboratory test.	Low: AD and aMCI diagnoses doesn't rely on patient recall	Low: clinician diagnosing AD/aMCI unlikely to be aware of exposure status	Low: ascertainment of AD/aMCI unlikely to be influenced by herpesvirus status.	Low: diagnosed via complete diagnostic evaluation.	No covariates	No covariates	Low: case control study	Unclear: missing data not reported	High: samples tested following AD/aMCI diagnosis	Generalisable to older inidividuals
Mancuso, 2016	High: no confounders taken into account.	High: No information on how cohort was sampled.	High: no information on how controls were selected.	Low: exposure status defined through brain sample tests: doesn't rely on patient recall	Low: observer blinded to outcome status.	Low: all cases and controls had brain samples tested.	Low: defined using laboratory test.	Low: AD diagnosis doesn't rely on patient recall	Low: clinician diagnosing AD unlikely to be aware of exposure status	Low: ascertainment of AD unlikely to be influenced by herpesvirus status.	Low: diagnosed using clinical criteria.	No covariates	No covariates	Low: case control study	Unclear: missing data not reported	High: as brain samples tested following AD diagnosis	Unclear
Mori, 2004	High: no confounders taken into account.	High: No information on how cohort was sampled.	High: no information on how controls were selected.	Low: exposure status defined through serum sample tests: doesn't rely on patient recall	Low: unclear if exposure status defined by observer blinded to outcome status.	Low: all cases and controls had serum samples tested.	Low: defined using laboratory test.	Low: AD diagnoses doesn't rely on patient recall	Unclear: Unclear how outcomes defined	Low: ascertainment of AD unlikely to be influenced by herpesvirus status.	Unclear: Unclear how outcomes defined	No covariates	No covariates	Low: case control study	Unclear: missing data not reported	High: samples tested following AD diagnosis	Generalisable to older inidividuals

Observational Studies																	
	**Confounding**	**Selection of participants**		**Misclassification of variables**										**Bias due to missing data**		**Reverse causation**	**Generalisability**
	Age and other confounders	Participation bias	Selection of controls	Exposure				Outcome				Covariates		Differential loss to follow up	Exclusion of individuals with missing data		
				Differential			Non-differential	Differential			Non-differential	Differential	Non-differential				
				Recall bias	Observer bias	Ascertainment bias		Recall bias	Observer bias	Ascertainment bias							
Roberts, 1986	High: no confounders taken into account.	High: No information on how cohort was sampled.	High: no information on how controls were selected.	Low: exposure status defined through brain sample tests: doesn't rely on patient recall	Low: unclear if exposure status defined by observer blinded to outcome status.	Low: all cases and controls had brain samples tested.	Low: defined using laboratory test.	Low: AD diagnosis doesn't rely on patient recall	Low: person diagnosing AD unlikely to be aware of exposure status	Low: ascertainment of AD unlikely to be influenced by herpesvirus status.	Unclear: little information on how AD was defined.	No covariates	No covariates	Low: case control study	Unclear: missing data not reported	High: as brain samples tested following AD diagnosis	Unclear
Wozniak, 2009	High: no confounders taken into account.	High: No information on how cohort was sampled.	High: no information on how controls were selected.	Low: exposure status defined through brain sample tests: doesn't rely on patient recall	Low: unclear if exposure status defined by observer blinded to outcome status.	Low: all cases and controls had brain samples tested.	Low: defined using laboratory test.	Low: AD diagnosis doesn't rely on patient recall	Low: person diagnosing AD unlikely to be aware of exposure status	Low: ascertainment of AD unlikely to be influenced by herpesvirus status.	Unclear: little information on how AD was defined.	No covariates	No covariates	Low: case control study	Unclear: missing data not reported	High: as brain samples tested following AD diagnosis	Unclear
HSV-1 reactivation																	
Helmer, 2015	Moderate: age adjusted but no detail	Moderate: No information on how cohort was sampled. Within cohort subsamples included if have baseline blood and available claims data at '2nd' f/u. No description of characteristics of included and non included	N/A (only for case control studies)	Low: exposure status defined through blood tests: doesn't rely on patient recall	Unclear: unclear if exposure status defined by observer blinded to outcome status.	Low: exposure status defined before dementia ascertained.	Unclear: no diagnosis information on tests used to define HSV-1	Low: dementia doesn't reply on patient recall	Unclear: not clear if observer defining dementia aware of exposure status.* check again what info observers had access to	Low: ascertainment of dementia unlikely to be influenced by herpesvirus status.	Unclear: little information given regarding dementia diagnosis* check how ascertained	Low because collected at baseline	Unclear	Low for part 2 as one criterion for inclusion is adequate f/u BUT unclear for part 1	Unclear: missing data not reported	Low: exposure recorded prior to ascertainment of dementia.	Reasonable as population cohort of older individuals
Epstein-Barr virus infection																	
Shim, 2017	Moderate: age adjusted but no detail	High: No info on how cohort was sampled. Not clear how cohort reduced from >100 at baseline to 36 in main analysis.	Low: nested case-control study therefore controls sampled from the population in which the cases arose.	Low: exposure status defined through blood tests: doesn't rely on patient recall	Low: unclear if exposure status defined by observer blinded to outcome status.	Low: exposure status defined before dementia ascertained.	Low: defined using laboratory test.	Low: dementia diagnosis doesn't rely on patient recall	Unclear: not clear if observer defining dementia aware of exposure status.	Low: ascertainment of dementia unlikely to be influenced by herpesvirus status.	Low: diagnosed via complete diagnostic evaluation.	Low: collected at specific follow-ups for cases and controls	Low	Low: case control study	Unclear: missing data not reported	Low: exposure recorded prior to ascertainment of dementia.	Generlaisable to older Korean individuals.
Cytomegalovirus infection																	
Lovheim, 2015a	Moderate: Tightly matched on age and gender. No other potential confounders investigated.	Unclear: not clear if *all* AD patients from original cohort included or if random/non-random sample.	Low: nested case-control study therefore controls sampled from the population in which the cases arose.	Low: exposure status defined through blood tests: doesn't rely on patient recall	Low: unclear if exposure status defined by observer blinded to outcome status.	Low: exposure status defined before dementia ascertained.	Low: defined using laboratory test.	Low: AD diagnosis doesn't rely on patient recall	Unclear: not clear if observer defining AD aware of exposure status.	Low: ascertainment of AD unlikely to be influenced by herpesvirus status.	Low: diagnosed in hospital and validated.	Low: age and gender only covariates	Low: age and gender only covariates	Low: case control study	Unclear	Low: exposure recorded prior to ascertainment of dementia.	Generalisable to older individuals
Kawasaki 2016	Moderate: age adjusted but no detail	Moderate. Although cohort was recruited through random sampling, no info was given on this. Also only 328 included of 494 because they had repeat MMSEs and no severe cognitive impairment at baseline. Characteristics of those with and without repeat MMSE are unclear.	N/A (only for case control studies)	Low	Low	Low	Low	Low	Low	Low	Low as active collection of outcome	Low because collected at baseline	Unclear	Unclear - note all accoutned for but quite a few died (76/494) and some either refused MMSE or did only a telephone survey (70/494) or had unknown survival status (18/494). Unclear if their characteristics differed	Unclear: missing data not reported	Low: exposure recorded prior to ascertainment of dementia.	Generalisable to older Japanese population
Renvoize, 1984	High: no confounders taken into account.	High: No information on how study sample was sampled.	Low: controls sampled from the hospitals in which the cases arose.	Low: exposure status defined through laboratory tests: doesn't rely on patient recall	Low: unclear if exposure status defined by observer blinded to outcome status.	Low: all cases and controls had laboratory samples tested.	Low: defined using laboratory test.	Low: AD diagnosis doesn't rely on patient recall	Unclear: not clear if observer defining AD aware of exposure status.	Low: ascertainment of AD unlikely to be influenced by herpesvirus status.	Low: robust definition of AD	No covariates	No covariates	Low: case control study	Unclear: missing data not reported	High: samples tested following outcome diagnosis	Unclear
Westman, 2013	High: no confounders taken into account.	Unclear: not clear how patients were enrolled into study (eg if random sampling was used).	Moderate: no information on how controls were selected, however they were derived from the same brain banks as the cases.	Low: exposure status defined through blood tests: doesn't rely on patient recall	Low: exposure status defined by observer blinded to outcome of patients.	Low: all cases and controls had brain samples tested.	Low: defined using laboratory test.	Low: AD diagnosis doesn't rely on patient recall	Low: observer defining AD not aware of exposure status.	Low: ascertainment of AD unlikely to be influenced by herpesvirus status.	Low: robust definition of AD	No covariates	No covariates	Low: case control study	Unclear: missing data not reported	High: as blood samples tested following AD diagnosis	Unclear
Human herpesvirus 8 infection																	
Dore, 1998	High: although CD4 cell count included in the model, age was not taken into account.	Unclear: not clear how patients were enrolled into cohort study (eg if random sampling was used).	N/A (only for case control studies)	Low: exposure status defined prior to outcome: doesn't rely on patient recall	Low: exposure status defined before outcome	Low: exposure status defined before outcome	Unclear: definition of exposure not well defined.	Low: outcome diagnosis doesn't rely on patient recall	High: very likely that observer defining ADC will be aware of patients KS status.	High: ascertainment of dementia may be influenced by KS status	Unclear: diagnois not well described.	CD4 cell counts measured in exposed and unexpposed	CD4 cell counts unlikely to be missclassified	Low: numbers lost to follow-up not given.	Unclear: missing data not reported	Low: exposure recorded prior to ascertainment of outcome	AIDs patients
Dupin, 2000	High: no confounders taken into account.	Unclear: although the cases and controls were randomly selected from the original cohort, it is not clear how patients were enrolled into the original cohort.	N/A (only for case control studies)	Low: exposure status defined prior to outcome: doesn't rely on patient recall	Low: exposure status defined before outcome	Low: exposure status defined before outcome	Unclear: definition of exposure not well defined.	Low: outcome diagnosis doesn't rely on patient recall	High: very likely that observer defining ADC will be aware of patients KS status.	High: ascertainment of dementia may be influenced by KS status	Unclear: diagnois not well described.	No covariates	No covariates	Low: case control study	Unclear: missing data not reported	Low: exposure recorded prior to ascertainment of outcome	AIDs patients
Mary-Krause, 1999	Low: Adjusted for age at diagnosis with several categories (10 years) and other variables.	Low: hospital-based multicentre open cohort with inclusions ongoing since 1989: included all those eligible.	N/A (only for case control studies)	Low: exposure status defined prior to outcome: doesn't rely on patient recall	Low: exposure status defined before outcome	Low: exposure status defined before outcome	Unclear: definition of exposure not well defined.	Low: outcome diagnosis doesn't rely on patient recall	High: very likely that observer defining ADC will be aware of patients KS status.	High: ascertainment of dementia may be influenced by KS status	Unclear: diagnois not well described.	Low: ascertained similarly for exposed and unexposed.	Unclear: little information on exact definition or ascertainment of covariates	Unclear: numbers lost to follow-up not given.	Low: missing data for CD4 cell count, but unlikely to bias results.	Low: exposure recorded prior to ascertainment of outcome	AIDs patients
Polk, 2002	Moderate: Tightly matched on age and HIV disease characteristics. No other potential confounders investigated.	Unclear: not clear how patients were enrolled into cohort study (eg if random sampling was used).	Low: nested case-control study therefore controls sampled from the population in which the cases arose.	Low: exposure status defined through blood tests: doesn't rely on patient recall	Low: exposure status defined by observer blinded to outcome status.	Low: exposure status defined before dementia ascertained.	Low: defined using laboratory test.	Low: dementia diagnosis doesn't rely on patient recall	Unclear: not clear if observer defining dementia aware of exposure status.	Low: ascertainment of dementia unlikely to be influenced by herpesvirus status.	Low: diagnosed via complete diagnostic evaluation.	Low: collected at specific follow-ups for cases and controls	Low	Low: case control study	Unclear: missing data not reported	Low: exposure recorded prior to ascertainment of dementia.	Generlaisable to homosexual men

Observational Studies																	
	Confounding	Selection of participants		Misclassification of variables										Bias due to missing data		Reverse causation	Generalisability
	Age and other confounders	Participation bias	Selection of controls	Exposure				Outcome				Covariates		Differential loss to follow up	Exclusion of individuals with missing data		
				Differential			Non-differential	Differential			Non-differential	Differential	Non-differential				
				Recall bias	Observer bias	Ascertainment bias		Recall bias	Observer bias	Ascertainment bias							
Renwick, 2001	High: age not adjusted for in main analysis (although authors report adjusting for age had no effect). Adjusted for CD4 cell count and ART but no other potential confounders considered.	Unclear: not clear how final participants were selected -was it *all* those eligible?	N/A (only for case control studies)	Low: exposure status defined through blood tests: doesn't rely on patient recall	Low: exposure status defined before outcome status ascertained	Low: exposure status defined before dementia ascertained.	Low: defined using laboratory test.	Low: dementia diagnosis doesn't rely on patient recall	Unclear: not clear if observer defining dementia aware of exposure status.	Low: ascertainment of dementia unlikely to be influenced by herpesvirus status.	Low: adequate diagnosis with good rule out of other potential causes	Low: collected at specific follow-ups for exposed and unexposed	Unclear	Unclear: follow-up time not reported	Unclear: missing data not reported	Low: exposure recorded prior to ascertainment of dementia.	Generlaisable to homosexual men with HIV
Rezza, 1999	Moderate: age adjusted (but no detail) as well as other risk factors accounted for.	Moderate: subsample of cohort analysed represented 26% of overall sample. Differed only in HIV exposure category (e.g homosexual men over-represented).	N/A (only for case control studies)	Low: exposure status defined through blood tests: doesn't rely on patient recall	Low: exposure status defined before outcome status ascertained	Low: exposure status defined before dementia ascertained.	Low: defined using laboratory test.	Low: dementia diagnosis doesn't rely on patient recall	Unclear: not clear if observer defining dementia aware of exposure status.	Low: ascertainment of dementia unlikely to be influenced by herpesvirus status.	Low: adequate diagnosis with good rule out of other potential causes	Low: collected at specific follow-ups for exposed and unexposed	Low: good ascertainment variables.	Unclear: follow-up time not reported	Unclear: missing data not reported	Low: exposure recorded prior to ascertainment of dementia.	Generlaisable to those with HIV
Multiple herpesviruses																	
Agostini, 2016a	High: no confounders taken into account.	Unclear	N/A (only for case control studies)	Low: exposure status defined through blood tests: doesn't rely on patient recall	Low: unclear if exposure status defined by observer blinded to outcome status.	Low: exposure status defined before dementia ascertained.	Low: defined using laboratory test.	Low: AD diagnosis doesn't rely on patient recall	Unclear: not clear if observer defining dementia aware of exposure status.	Low: ascertainment of dementia unlikely to be influenced by herpesvirus status.	Unclear: little information given regarding dementia diagnosis	No covariates relevant as we are preseting crude OR only	No covariates relevant as we are preseting crude OR only	Unclear: numbers lost to follow-up not given.	Unclear: missing data not reported	Low: exposure recorded prior to ascertainment of dementia.	Generalisable to older inidividuals
Agostini, 2016b	High: no confounders taken into account.	High: No information on how cohort was sampled or how many refused to participate.	Moderate: controls not selected randomly, but did derive from same population as cases (spouses of cases).	Low: exposure status defined through blood tests: doesn't rely on patient recall	Low: unclear if exposure status defined by observer blinded to outcome status.	Low: all cases and controls had serum samples tested.	Low: defined using laboratory test.	Low: AD diagnosis doesn't rely on patient recall	Unclear: not clear if observer defining dementia aware of exposure status.	Low: ascertainment of dementia unlikely to be influenced by herpesvirus status.	Low: diagnosed using diagnostic criteria.	No covariates relevant as we are preseting crude OR only	No covariates relevant as we are preseting crude OR only	Low: case control study	Unclear: missing data not reported	High: samples tested following outcome diagnosis	Generalisable to older inidividuals
Aiello 2006	Moderate (age adjusted but no detail)	Moderate. Of the initial cohort, 67% had baseline blood and at least 2 years f/u so were included. Those included were younger, had lower proportion born in Mexico and had higher education and income than those not included	Low: nested case-control study therefore controls sampled from the population in which the cases arose.	Low: exposure status defined through blood tests: doesn't rely on patient recall	Low: exposure status defined by observer blinded to outcome status.	Low: exposure status defined before cognitive decline measured.	Low: tests for herpesviruses have high specificity and sensitivity.	Low: level of cognitive decline doesn't reply on patient recall	Unclear: not clear if observer defining level of cognition aware of exposure status.	Low: ascertainment of cognitive decline unlikely to be influenced by herpesvirus status.	Low: Validated methods used to deterine level of cognitive function.	Low: unlikely to be affected by exposure or outcome status	Low: There were 80 individuals (around 6.6%) with missing data on APOE4	Unclear but participants needed at least 2 years of f/u to be included	Low because used complete case analysis with respect to main variables	Low: exposure defined prior to ascertainment of cognitive decline	May not be that high as specific older Latino population, almost all of whom had antibodies to CMV and HSV1
Barnes, 2015	Moderate: age adjusted (but no detail) as well as other risk factors accounted for.	Moderate: random subsample of cohorts analysed - no comparison of sample with the original cohorts.	N/A (only for case control studies)	Low: exposure status defined through blood tests: doesn't rely on patient recall	Low: exposure status defined by observer blinded to all clinical characteristics.	Low: exposure status defined before dementia ascertained.	Low: defined using laboratory test.	Low: AD diagnosis doesn't rely on patient recall	Low: clinician diagnosing AD blinded to all previously collected information.	Low: ascertainment of AD unlikely to be influenced by herpesvirus status.	Low: diagnosed in hospital and validated.	Low: collected at specific follow-ups for exposed and unexposed	Low: good ascertainment variables.	Low: numbers lost to follow-up not given. However average follow-up was 5 years.	Unclear: missing data not reported	Low: exposure recorded prior to ascertainment of dementia.	Generalisable to older individuals
Bu, 2015	Moderate: age adjusted (but no detail) as well as other risk factors accounted for.	Moderate: non-random sampling (consecutive recruitment); good description of exclusion criteria but no reporting of numbers excluded.	Low: controls selected randomly, from same population as cases.	Low: exposure status defined through blood tests: doesn't rely on patient recall	Low: exposure status defined by observer blinded to outcome status.	Low: all cases and controls had serum samples tested.	Low: defined using laboratory test.	Low: AD diagnosis doesn't rely on patient recall	Unclear: not clear if observer defining dementia aware of exposure status.	Low: ascertainment of dementia unlikely to be influenced by herpesvirus status.	Low: diagnosed using diagnostic criteria.	Low: collected similarly for cases and controls	Unclear: little information on exact definition or ascertainment of covariates	Low: case control study	Unclear: missing data not reported	High: samples tested following AD diagnosis	Generalisable to older inidividuals
Carbone, 2014	High: no confounders taken into account.	High: of original cohort of (n=1016), with blood sample (n=985, 97%), not clear how current sample were selected, or how they differed.	Moderate: controls were selected from the same population from which cases arose (i.e. same cohort) but not clear how they were selected (eg were they matched or randomly selected?)	Low: exposure status defined through blood tests: doesn't rely on patient recall	Low: unclear if exposure status defined by observer blinded to outcome status.	Low: exposure status defined before dementia ascertained.	Low: defined using laboratory test.	Low: AD diagnosis doesn't rely on patient recall	Unclear: not clear if observer defining dementia aware of exposure status.	Low: ascertainment of dementia unlikely to be influenced by herpesvirus status.	Unclear: little information given regarding dementia diagnosis	No covariates relevant as we are preseting crude OR only	No covariates relevant as we are preseting crude OR only	Low: case control study	High: Number of cases and controls in methods doesn't tally with results, however no explanation as to why patients exposure status was missing.	High as blood sample taken at similar time to dementia diagnosis (except in additional analysis)	Generalisable to older individuals
Deng 2016	High. Although age was adjusted for, the analysis method was not appropriate to a case control study	High. Although clear sampling frame (employees of a large organisation) it is not clear how /whether those included (280 ish) included differ from the >100,000 excluded	Moderate - while it appears that all eligible to be controls were included the eligibility criteria meanthat there were systematic differences from cases e.g. cases needed a caregiver 4 days a week. Not so for controls…	Low: exposure status defined through blood tests: doesn't rely on patient recall	Unclear: not clear if observer blinded to case/control status	Low: all cases and controls had to have a blood test	Unclear: no information on tests used to define HSV-1	Low: case/control status defined prior to exposures defined	Low: case/control status defined prior to exposures defined	Low: ascertainment of VCIND unlikely to be influenced by herpesvirus status.	High: not clear if controls were required to be tested for cognitive impairment	No covariates relevant as we are preseting crude OR only	No covariates relevant as we are preseting crude OR only	Low: case control study	Unclear	High as blood sample taken at similar time to o/c diagnosis	Generalisable to older people in employment in China (note query about employment status?)
Hemling, 2003	Moderate: Controlled for age (but no detail of how age was categorised) and gender.	High: No information on how study sample was sampled.	High: no information on how controls were selected.	Low: exposure status defined through brain sample tests: doesn't rely on patient recall	Low: unclear if exposure status defined by observer blinded to outcome status.	Low: all cases and controls had to have a blood test	Low: defined using laboratory test.	Low: AD diagnosis doesn't rely on patient recall	Unclear: not clear if observer defining AD aware of exposure status.	Low: ascertainment of AD unlikely to be influenced by herpesvirus status.	Low: robust definition of AD	Low: age and gender only covariates	Low: age and gender only covariates	Low: case control study	Unclear: missing data not reported	High: as blood samples tested following AD diagnosis	Generalisable to older individuals
Itzhaki, 2005	High: no confounders taken into account.	High: No information on how cohort was sampled.	High: no information on how controls were selected.	Low: little information on exposure definition, but cannot rely on patient recall	Low: unclear if exposure status defined by observer blinded to outcome status.	Unclear: unclear if all cases and controls had brain samples tested.	Unclear: no information on tests	Low: AD diagnosis doesn't rely on patient recall	Unclear: not clear if observer defining AD aware of exposure status.	Low: ascertainment of AD unlikely to be influenced by herpesvirus status.	Unclear: no information on how AD was defined.	No covariates	No covariates	Low: case control study	Unclear: missing data not reported	High: as brain samples tested following AD diagnosis	Unclear

Observational Studies	Confounding: Age and other confounders	Selection of participants: Participation bias	Selection of participants: Selection of controls	Misclassification of variables: Exposure, Differential: Recall bias	Exposure, Differential: Observer bias	Exposure, Differential: Ascertainment bias	Exposure: Non-differential	Outcome, Differential: Recall bias	Outcome, Differential: Observer bias	Outcome, Differential: Ascertainment bias	Outcome: Non-differential	Covariates: Differential	Covariates: Non-differential	Bias due to missing data: Differential loss to follow up	Bias due to missing data: Exclusion of individuals with missing data	Reverse causation	Generalisability
Kittur, 1992	High: no confounders taken into account.	Moderate: No information on how cases were sampled from the dementia clinic.	Moderate: no information on how controls were selected from the Cohort study on Ageing or whether the cohort represented the population from which the cases arose.	Low: exposure status defined through blood tests: doesn't rely on patient recall	Low: unclear if exposure status defined by observer blinded to outcome status.	Low: all cases and controls had blood samples tested.	Low: defined using laboratory test.	Low: AD diagnosis doesn't rely on patient recall	Low: clinician diagnosing AD unlikely to be aware of exposure status	Low: ascertainment of AD unlikely to be influenced by herpesvirus status.	Low: diagnosed using diagnostic criteria.	No covariates	No covariates	Low: case control study	Unclear: missing data not reported	High: as blood samples tested following AD diagnosis	Unclear
Letenneur, 2008	Moderate: age adusted for (but not detail of how age categorised) and good adjustment for other key confounders	High: of original cohort, 15.6% (591/3777) had blood samples taken, but no explanation if this is a random/representive sample.	N/A (only for case control studies)	Low: exposure status defined through blood tests: doesn't rely on patient recall	Low: unclear if exposure status defined by observer blinded to outcome status.	Low: exposure status defined before dementia ascertained.	Low: defined using laboratory test.	Low: AD diagnosis doesn't rely on patient recall	Unclear: not clear if observer defining dementia aware of exposure status.	Low: ascertainment of dementia unlikely to be influenced by herpesvirus status.	Low: Rigorous definition of dementia applied and confirmed by psychologist and neurologist	Low: collected at baseline.	Low: covariates well-defined	Unclear: numbers lost to follow-up not given.	Unclear	Low: exposure recorded prior to ascertainment of dementia.	Generalisable to older individuals
Lin, 1996	High: no confounders taken into account.	High: No information on how study sample was sampled.	High: no information on how controls were selected.	Low: exposure status defined through brain sample tests: doesn't rely on patient recall	Low: exposure status defined by observer blinded to outcome status.	Low: all cases and controls had brain samples tested.	Low: defined using laboratory test.	Low: AD diagnosis doesn't rely on patient recall	Low: observer defining dementia unaware of exposure status.	Low: ascertainment of dementia unlikely to be influenced by herpesvirus status.	Unclear: little information given regarding dementia diagnosis	No covariates	No covariates	Low: case control study	Unclear: missing data not reported	High: samples tested following AD diagnosis	Unclear
Lin, 2002a	High: no confounders taken into account.	High: No information on how study sample was sampled.	High: no information on how controls were selected.	Low: exposure status defined through brain sample tests: doesn't rely on patient recall	Low: unclear if exposure status defined by observer blinded to outcome status.	Unclear: total number of cases and controls sampled not given. Not clear if all cases and controls had brain samples tested.	Low: defined using laboratory test.	High: Unclear how outcome defined	Unclear: not clear if observer defining dementia aware of exposure status.	Low: ascertainment of dementia unlikely to be influenced by herpesvirus status.	Unclear: little information given regarding dementia diagnosis	No covariates	No covariates	Low: case control study	High: issue of missing data raised, but no numbers reported	High: samples tested following AD diagnosis	Unclear
Lin, 2002b	High: no confounders taken into account.	High: No information on how study sample was sampled.	High: no information on how controls were selected.	Low: exposure status defined through brain sample tests: doesn't rely on patient recall	Low: unclear if exposure status defined by observer blinded to outcome status.	Low: all cases and controls had brain samples tested.	Low: defined using laboratory test.	High: Unclear how outcome defined	Unclear: not clear if observer defining VaD aware of exposure status.	Low: ascertainment of VaD unlikely to be influenced by herpesvirus status.	Unclear: little information given regarding VaD diagnosis	No covariates	No covariates	Low: case control study	Unclear: missing data not reported	High: samples tested following VaD diagnosis	Unclear
Lovheim, 2015b	Moderate: Tightly matched on age and gender. No other potential confounders investigated.	Unclear: not clear if *all* AD patients from original cohort included or if random/non-random sample.	Low: nested case-control study therefore controls sampled from the population in which the cases arose.	Low: exposure status defined through blood tests: doesn't rely on patient recall	Low: unclear if exposure status defined by observer blinded to outcome status.	Low: exposure status defined before dementia ascertained.	Low: defined using laboratory test.	Low: AD diagnosis doesn't rely on patient recall	Unclear: not clear if observer defining AD aware of exposure status.	Low: ascertainment of AD unlikely to be influenced by herpesvirus status.	Low: robust definition of AD	Low: age and gender only covariates	Low: age and gender only covariates	Low: case control study	Unclear	Low: exposure recorded prior to ascertainment of dementia.	Generalisable to older individuals
Lovheim, 2015c	Moderate: age adusted for (but not detail of how age categorised). Investigated other potential confounders.	Low: representative sample.	N/A (only for case control studies)	Low: exposure status defined through blood tests: doesn't rely on patient recall	Low: unclear if exposure status defined by observer blinded to outcome status.	Low: exposure status defined before dementia ascertained.	Low: defined using laboratory test. Also confirmed a subsample through an alternative immunofluoes cence assay.	Low: AD diagnosis doesn't rely on patient recall	Unclear: not clear if observer defining AD aware of exposure status.	Low: ascertainment of AD unlikely to be influenced by herpesvirus status.	Low: robust definition of AD	Low: collected at baseline.	Low: covariates well-defined	Low: 3.9% people lost to follow-up.	Unclear: APOE genotype	Low: exposure recorded prior to ascertainment of dementia.	Generalisable to older individuals
Lycke, 1974	High: no confounders taken into account.	High: No information on how study sample was sampled.	High: no information on how controls were selected.	Low: exposure status defined through blood sample tests: doesn't rely on patient recall	Low: unclear if exposure status defined by observer blinded to outcome status.	Low: all cases and controls had blood samples tested.	Low: defined using laboratory test.	High: Unclear how outcome defined	Unclear: not clear if observer defining outcome aware of exposure status.	Low: ascertainment of outcome unlikely to be influenced by herpesvirus status.	Unclear: little information given regarding outcome diagnosis	No covariates	No covariates	Low: case control study	Unclear: missing data not reported	High: samples tested following outcome diagnosis	Unclear
Mancuso, 2014b	High: no confounders taken into account. Possible correlations between Ab titers and age were analyzed but results were negative.	Unclear: not clear how patients were enrolled into study (eg if random sampling was used).	Moderate: controls selected according to well-defined criteria, but not clear same population from which the cases arose.	Low: exposure status defined through blood sample tests: doesn't rely on patient recall	Low: unclear if exposure status defined by observer blinded to outcome status.	Low: all cases and controls had blood samples tested.	Low: defined using laboratory test.	Low: AD diagnosis doesn't rely on patient recall	Unclear: not clear if observer defining AD aware of exposure status.	Low: ascertainment of AD unlikely to be influenced by herpesvirus status.	Low: robust definition of AD	No covariates	No covariates	Low: case control study	Unclear: missing data not reported	High: samples tested following outcome diagnosis	Unclear
Mann, 1981	High: no confounders taken into account.	High: No information on how study sample was sampled.	High: no information on how controls were selected.	Low: exposure status defined through brain sample tests: doesn't rely on patient recall	Low: unclear if exposure status defined by observer blinded to outcome status.	Low: all cases and controls had brain samples tested.	Low: defined using laboratory test.	Low: AD diagnosis doesn't rely on patient recall	Unclear: not clear if observer defining AD aware of exposure status.	Low: ascertainment of AD unlikely to be influenced by herpesvirus status.	Low: robust definition of AD	No covariates	No covariates	Low: case control study	Unclear: missing data not reported	High: samples tested following outcome diagnosis	Unclear
Marques, 2001	High: no confounders taken into account.	High: No information on how cohort was sampled.	High: no information on how controls were selected.	Low: exposure status defined through serum sample tests: doesn't rely on patient recall	Low: unclear if exposure status defined by observer blinded to outcome status.	Low: all cases and controls had serum samples tested.	Low: defined using laboratory test.	High: Unclear how outcomes (AD and MCI) defined)	High: Unclear how outcomes (AD and MCI) defined)	High: Unclear how outcomes (AD and MCI) defined)	High: Unclear how outcomes (AD and MCI) defined)	No covariates	No covariates	Low: case control study	Unclear: missing data not reported	High: samples tested following AD diagnosis	Unclear

Observational Studies																		
	Confounding	Selection of participants		Misclassification of variables										Bias due to missing data		Reverse causation	Generalisability
	Age and other confounders	Participation bias	Selection of controls	Exposure				Outcome				Covariates		Differential loss to follow up	Exclusion of individuals with missing data		
				Differential			Non-differential	Differential			Non-differential	Differential	Non-differential				
				Recall bias	Observer bias	Ascertainment bias		Recall bias	Observer bias	Ascertainment bias							
Ounanian, 1990	High: no confounders taken into account: design matched on age, but unclear how closely.	High: No information on how study sample was sampled.	High: no information on how controls were selected.	Low: exposure status defined through blood sample tests: doesn't rely on patient recall	Low: unclear if exposure status defined by observer blinded to outcome status.	Low: all cases and controls had brain samples tested.	Low: defined using laboratory test.	Low: AD diagnosis doesn't rely on patient recall	Unclear: not clear if observer defining AD aware of exposure status.	Low: ascertainment of AD unlikely to be influenced by herpesvirus status.	Low: robust definition of AD	No covariates	No covariates	Low: case control study	Unclear: missing data not reported	High: samples tested following outcome diagnosis	Unclear
Renvoize, 1987	High: no confounders taken into account.	High: No information on how study sample was sampled.	Unclear: not clear if cases were selected from the same hospital in which the controls had been admitted to.	Low: exposure status defined through laboratory tests: doesn't rely on patient recall	Low: unclear if exposure status defined by observer blinded to outcome status.	Low: all cases and controls had laboratory samples tested.	Low: defined using laboratory test.	Low: AD diagnosis doesn't rely on patient recall	Unclear: not clear if observer defining AD aware of exposure status.	Low: ascertainment of AD unlikely to be influenced by herpesvirus status.	Low: robust definition of AD	No covariates	No covariates	Low: case control study	Unclear: missing data not reported	High: samples tested following outcome diagnosis	Unclear
Strandberg, 2003	Moderate: only MMSE score adjusted for, although the authors report that MMSE score was already dictated by age and educational level	Low: random sample	N/A (only for case control studies)	Low: exposure status defined through blood tests: doesn't rely on patient recall	Low: exposure status defined by observer blinded to all clinical details of patients.	Low: exposure status defined before cognitive decline ascertained.	Low: defined using laboratory test.	Low: cognitive decline doesn't rely on patient recall	Low: observer defining cognitive decline not aware of exposure status.	Low: ascertainment of AD unlikely to be influenced by herpesvirus status.	Low: robust definition of cognitive decline	Low: only MMSE score at baseline.	Low: only MMSE score at baseline.	Low: 9% people lost to follow-up.	Unclear	Low: exposure recorded prior to ascertainment of cognitive decline	Generalisable to older individuals
Taylor, 1986	High: no confounders taken into account.	High: No information on how cohort was sampled from the individual brain banks	Moderate: no information on how controls were selected, however they were derived from the same brain banks as the cases.	Low: exposure status defined through brain sample tests: doesn't rely on patient recall	Low: unclear if exposure status defined by observer blinded to outcome status.	Low: all cases and controls had brain samples tested.	Low: defined using laboratory test.	Low: AD diagnosis doesn't rely on patient recall	Unclear: not clear if observer defining AD aware of exposure status.	Low: ascertainment of AD unlikely to be influenced by herpesvirus status.	Low: robust definition of AD	No covariates	No covariates	Low: case control study	Unclear: missing data not reported	High: as brain samples tested following AD diagnosis	Unclear
Westman, 2017	High: no confounders taken into account.	Unclear: not clear how patients were enrolled into study (eg if random sampling was used).	Moderate: no information on how controls were selected, however they were derived from the same brain banks as the cases.	Low: exposure status defined through blood tests: doesn't rely on patient recall	Low: exposure status defined by observer blinded to outcome of patients.	Low: all cases and controls had brain samples tested.	Low: defined using laboratory test.	Low: AD diagnosis doesn't rely on patient recall	Low: observer defining AD not aware of exposure status.	Low: ascertainment of AD unlikely to be influenced by herpesvirus status.	Low: robust definition of AD	No covariates	No covariates	Low: case control study	Unclear: missing data not reported	High: as blood samples tested following AD diagnosis	Unclear
Wozniak, 2005	High: although cases and controls were age matched, we are not told how closely. No other confounders taken into account.	Unclear: Although the four brain banks were clearly named, it is not clear how patients were enrolled into each bank (eg if random sampling was used).	Moderate: no information on how controls were selected, however some were derived from the same brain banks as the cases.	Low: exposure status defined through laboratory tests: doesn't rely on patient recall	Low: unclear if exposure status defined by observer blinded to outcome status.	Low: all cases and controls had samples tested.	Low: defined using laboratory test.	Low: AD diagnosis doesn't rely on patient recall	Unclear: not clear if observer defining AD aware of exposure status.	Low: ascertainment of AD unlikely to be influenced by herpesvirus status.	Low: robust definitions of AD	No covariates	No covariates	Low: case control study	Unclear: missing data not reported	High: as samples tested following AD diagnosis	Unclear

Randomised Controlled Trials						
	Selection bias		Performance bias	Detection bias	Attrition bias	Reporting bias
	Random sequence generation	Allocation concealment				
Criteria for risk of bias assessment	**Low: a**dequate generation of a randomised sequence **High:** inadequate generation of a randomised sequence.	**Low:** adequate concealment of allocations prior to assignment **High:** inadequate concealment of allocations prior to assignment	**Low:** participants and personnel effectively blinded to intervention arm **High:** Neither participants, nor personnel, blinded to intervention arm.	**Low:** outcome assessors effectively blinded to intervention a participant received. **High:** Outcome assessors not blinded to knowledge of intervention.	**Low:** very little incomplete outcome data, and well-reported (e.g. numbers by arm and reasons for attrition). **High:** large amount of incomplete outcome poorly reported.	**Low:** A low possibility of selective outcome reporting. **High:** a strong possibility of selective data, outcome reporting.
Treatment for herpesviruses (e.g. antivirals such as acyclovir)						
Gnann, 2000	Low: Patients randomised to treatments; allotments were computer-generated.	Low: randomization scheme kept confidential until after database closed.	Low: double blind study - identical placebo tablets used for control arm.	Unclear: double-blind study, therefore researchers were blinded, however it is not explicitly stated that the outcome assessors were blinded.	Low: 8/87 (9%) patients lost to follow-up: numbers were reported, though reasons for attrition not given.	Low: Primary and secondary endpoints well described in methods and results

NB For each domain a-priori criteria were set-out to assess whether the domain be classified as 'high risk' (if very inadequately addressed), 'moderate risk' (if somewhat inadequately addressed), 'low risk' (if adequately addressed) or 'unclear risk' (if information is insufficient to formulate a judgement).
